# Using Flipped Classroom Modules to Facilitate Higher Order Learning in Undergraduate Organic Chemistry

**DOI:** 10.1021/acs.jchemed.3c00907

**Published:** 2024-01-25

**Authors:** Lauren R. Holloway, Tabitha F. Miller, Bryce da Camara, Paul M. Bogie, Briana L. Hickey, Angie L. Lopez, Jiho Ahn, Eric Dao, Nicole Naibert, Jack Barbera, Richard J. Hooley, Jack F. Eichler

**Affiliations:** † Department of Chemistry, University of California − Riverside, Riverside, California 92521, United States; ‡ Portland Community College, Portland, Oregon 97201, United States; § Portland State University, Portland, Oregon 97280, United States

**Keywords:** Undergraduate, Organic, Active Learning, Flipped Classroom

## Abstract

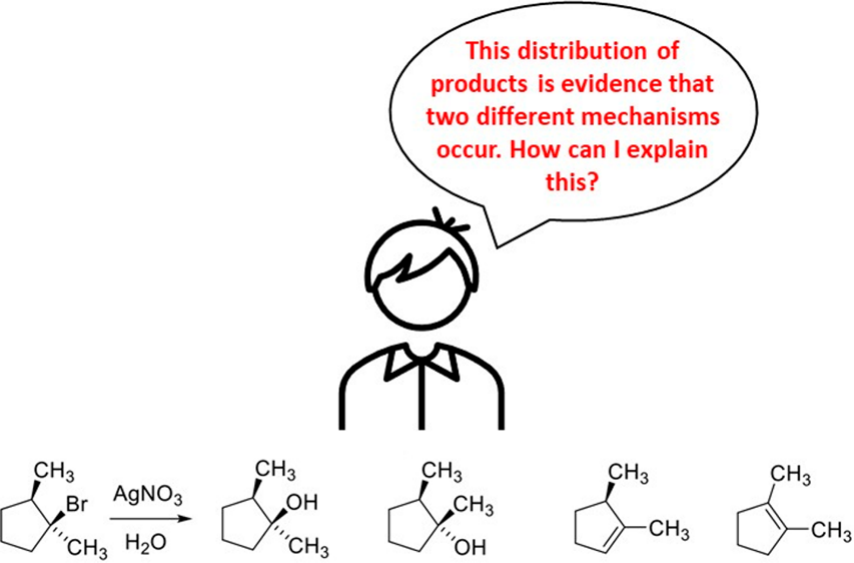

In an ongoing effort to incorporate active learning and promote higher order learning outcomes in undergraduate organic chemistry, a hybrid (“flipped”) classroom structure has been used to facilitate a series of collaborative activities in the first two courses of the lower division organic chemistry sequence. An observational study of seven classes over a five-year period reveals there is a strong correlation between performance on the in-class activities and performance on the final exam across all classes; however, a significant number of students in these courses continue to struggle on both the in-class activities and final exam. The Activity Engagement Survey (AcES) was administered in the most recent course offering included in this study, and these preliminary data suggest that students who achieved lower scores on the in-class activities had lower levels of emotional and behavioral/cognitive engagement and were less likely to work in collaborative groups. In total, these findings suggest that if students can be guided to engage more successfully with the in-class activities, they are likely to be more successful in carrying out the higher order learning required on the final exam. In addition to the analyses of student performance and engagement in the in-class activities, the implementation of the flipped classroom structure and suggestions for how student engagement in higher order learning might be improved in future iterations of the class are described herein.

## 1 Introduction

Calls for science education reform in the United States go back decades,^1,2^ yet achieving widespread change continues to be elusive. These broader reform efforts were reviewed in 2016, showing that there is a still-present need to improve STEM instruction in higher education, both by increasing active learning in the classroom and creating instructional practices that allow students to apply their knowledge in broader contexts.^3^ From a broader perspective, it has been pointed out that the lack of engaging classroom instruction is an ongoing barrier to student success improving equity gaps in higher education STEM.^4^ From a more specific chemical education research (CER) perspective, it was reported that a theme that arose from the 2022 BCCE symposium on the future of CER (“CER at a Crossroads: Where Do We Need to Go Now?”) was the need to accelerate the widespread adoption of scholarly teaching practices.^5^

In response, there is an initiative at the University of California, Riverside (UCR) to improve student success and retention in the large enrollment gateway courses. One area of focus has been the design and implementation of in-person-online hybrid course structures, known more commonly as flipped classrooms. The use of the flipped classroom structure continues to grow in higher education chemistry instruction^6^ and this can now be considered an evidence-based instructional practice.^7^ Though there is a general efficacy on student performance, most studies measure the impact of the flipped classroom using course grades and/or traditional end-of-course exams^8^ and much less attention has been devoted to determining how this might promote learning objectives that fall higher on Bloom’s taxonomy.^6^ A 2021 study described a flipped classroom that supported the implementation and assessment of concept development activities in a large enrollment general chemistry course,^9^ and it was reported in 2022 that PhET simulations can help students gain particulate-level understanding of equilibrium reactions. Importantly, this study used assessment items that required students to provide explanations of their thinking and not just “right” or “wrong” answers to traditional test items.^10^ However, continuing to explore how the flipped classroom might facilitate a deeper understanding of fundamental chemistry concepts is of interest to the chemistry education community.

It is within this context that this study is couched. To improve higher order thinking by students in a large-enrollment undergraduate lower-division organic chemistry class, in-class activities were developed in which students were challenged to perform detailed, multistep written questions in an active learning environment. These activities required the application of multiple different sophomore organic chemistry concepts, including arrow pushing, acid–base chemistry, reaction outcomes and mechanisms, and spectroscopic analysis. In each case, students were required to articulate their scientific reasoning as to why they chose their solution, not merely write down an answer. Flipped classroom modules were employed to streamline the integration of these activities into large enrollment classes that had previously relied mostly on didactic lecture.

### 1.1 Theoretical Frameworks of Learning

The theoretical frameworks of learning that guide the design of flipped classroom structures have been previously described.^11^ Cognitive load theory and constructivism were identified as the frameworks upon which flipped classrooms should be built. Cognitive load theory suggests that transferring some content to a preclass learning environment will allow students to intake new knowledge in a manageable manner and avoid overwhelming their memory capacity. If the time saved in the classroom is then used to create activities with a constructivist framework, students should actively generate new knowledge rather than passively absorbing it.^11^ As preclass learning activities often include video-based multimedia, this is also informed by Mayer’s cognitive theory of multimedia learning.^12^ Recently, existing online video content related to the general chemistry curriculum was evaluated using Mayer’s principles of multimedia design, which provided a detailed review of the cognitive theory of multimedia learning and noted that video content should aim to reduce extraneous load, manage essential processing, and foster generative processing.^13^ Flipped classrooms that incorporate online video content into the preclass phase of the course should carefully monitor these factors.

One of the goals of our study is to determine how higher order thinking could be integrated into a large enrollment gateway organic chemistry course. This necessitates the following question: what is the definition of higher order learning? Many educators use Bloom’s taxonomy to differentiate “lower order” vs “higher order” learning.^14^ For instance, one could classify the Remember and Understand categories as lower-level, whereas the Apply, Analyze, and Evaluate categories could be considered to be higher order processes. Because Bloom’s taxonomy is more of a way to classify types of learning objectives rather than an actual framework of learning, we propose that Ausubel’s theory of learning is more appropriate to describe the process of engaging in higher order learning,^15^ which others have argued.^16^ This commentary identifies three criteria of higher order learning to which Ausubel’s theory conforms: (1) the utilization of abstract structures for thinking; (2) the organization of information into an integrated system; and (3) the application of sound rules of judgment and logic.^16^ This proposes that Ausubel’s notion of meaningful learning is integral to higher order learning, since that requires the learner to possess a large existing mental framework of knowledge into which new ideas can be integrated in a sensible manner.^16^ Ausubel’s theory encapsulates the types of reasoning expected of students in organic chemistry courses, so it is highly relevant here.

Though the in-class activities described here were not directly influenced by the three-dimensional (3D) learning framework, it is acknowledged that this is also a viable framework through which to describe higher order learning. It has been proposed that this type of thinking should guide higher education STEM instruction,^3^ noting that even though active learning is generally associated with improved course grades and retention, active learning on its own may not imply higher order thinking. In fact, students who participated in courses with significant levels of active learning often retained misunderstandings of key conceptual ideas in different disciplines. The 3D learning framework addresses this shortcoming by explicitly having students develop knowledge around disciplinary core ideas, use their knowledge to carry out scientific practices, and link this knowledge to crosscutting concepts across different scientific disciplines.^3^ As this framework captures the essence of higher order learning, we will also identify which elements of the 3D learning framework are present in our study.

### 1.2 Observational Research Study Questions

Flipped classroom modules were implemented in seven courses across four different academic years, 2017–2023 (the years in which normal instruction was disrupted by COVID-19 policies were excluded). The primary goal was to leverage the flipped classroom structure to deploy detailed, handwritten learning activities in class, in a large enrollment course. Additionally, an important goal was to demonstrate that this type of high-effort teaching could be performed in a large gateway course, with minimal teaching assistant support and without resorting to automated questions. To gain insight into the impact of these activities and how they might be improved, the following research questions were explored:

1. What is the impact of the in-class module activities on the performance of final exams that require students to provide explanations of their reasoning (i.e., higher order thinking)?
2. What differences in behavioral/cognitive and/or emotional engagement exist during the in-class module activities when comparing the higher and lower performing students?

## 2 Methods

### 2.1 Flipped Classroom Implementation

**Figure 1:**
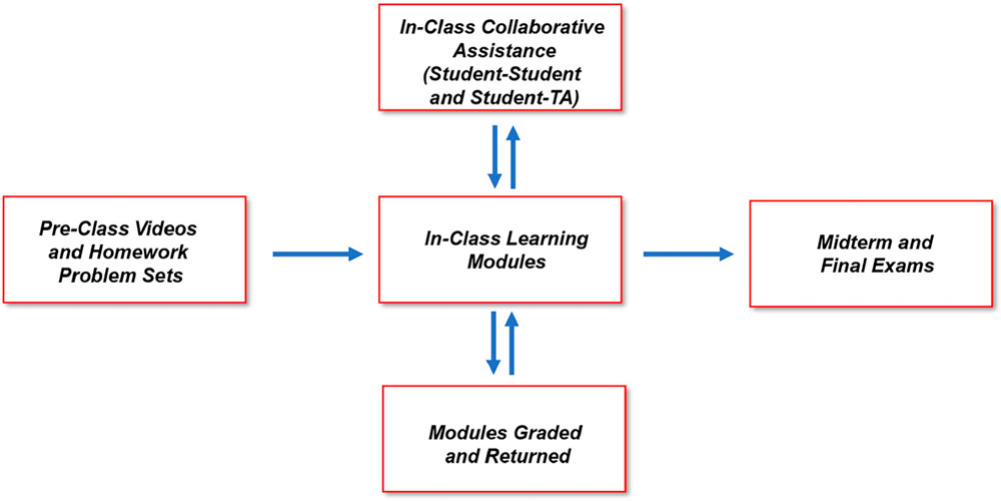
Overview of the flipped classroom implementation for CHEM 008A and 008B.

As part of a flipped classroom structure, the in-class module activities were administered in five (CHEM 008B) or six (CHEM 008A) classroom periods over the 10-week quarter (approximately three h of class time was set aside in each quarter for in-class learning activities). The students were given hard copies of the learning module activity (see Supporting Information (SI) Appendix 1 for examples) and were given 25–30 min to answer the questions. The students were encouraged to work in groups while completing the module, and facilitators were present throughout to answer questions. As these were large classes (>200 students) in an auditorium-style lecture theater, the students were allowed to walk around the class if they chose and cluster in groups to better enable collaborative learning. The modules were then graded by a single TA and returned to the students with a detailed answer key. The questions in the modules were derived from old exam questions in the class and had a layout and content similar (but not identical) to questions given to the students in the final exam, thus they could study the modules before relevant midterms and finals. The time used for the modules was supplemented by out-of-class learning videos, which were not directly related to the modules but covered core concepts in organic chemistry. An overview of the flipped classroom structure is depicted in Figure 1 and the detailed description of the class implementation is provided in SI Appendix 1A (including descriptions of the preclass video content and the preclass problem sets used to activate student prior knowledge).

### 2.2 Analysis of Final Exam Performance and AcES Survey

To address research question 1, the impact of the in-class module activities on final exam performance was evaluated by using the total module score median to create two study groups for each course (Table 1). Students who scored at or above the total module median score were labeled “High Module” and those who scored below the median were labeled “Low Module” (for the Fall 2018 008A, Fall 2019 008A, Winter 2018 008B, and Winter 2023 008B courses the median module scores were evenly distributed to the High and Low Module groups due to the large number of scores at the median value). Because no pretest was administered at the beginning of the courses, the variance in incoming student academic performance was accounted for by obtaining the overall university GPA for all students (GPA prior to the start of the course). It is noted that university GPA can vary between students based on the distribution of courses taken (i.e., the difficulty of courses can vary for different majors and the types of nonmajor courses taken, course loads can vary between students, etc.). However, because university GPA was significantly (and positively) correlated to in-class module activity performance in all of the cohorts included in this study and significantly (and positively) correlated to final exam performance for all the cohorts included in this study except for the Fall 2019 course (see SI Appendix 2B–H), it was prudent to include this as a covariate in the analysis. These data were obtained in a post hoc fashion under an exempt protocol approved by the UCR Institutional Review Board.

**Table 1:** Summary of Descriptive Statistics^a^

Class/Study Group	Final Exam Mean	University GPA Mean
Fall 2017 008A		
High Module (*n* = 106)	120 ± 33	3.05 ± 0.53
Low Module (*n* = 87)	77.4 ± 29.4	2.77 ± 0.52
Module Median = 16		
Fall 2018 008A		
High Module (*n* = 132)	116 ± 35	3.25 ± 0.49
Low Module (*n* = 132)	79.6 ± 31.4	3.05 ± 0.39
Module Median = 18		
Fall 2019 008A		
High Module (*n* = 132)	120 ± 31	3.11 ± 0.45
Low Module (*n* = 132)	86.7 ± 31	2.99 ± 0.52
Module Median = 16		
Fall 2022 008A		
High Module (*n* = 122)	122 ± 43	3.47 ± 0.41
Low Module (*n* = 130)	67.9 ± 29.1	2.84 ± 0.52
Module Median = 15		
Winter 2018 008B^b^		
High Module (*n* = 103)	121 ± 39	3.22 ± 0.45
Low Module (*n* = 103)	84.4 ± 33.1	3.04 ± 0.41
Module Median = 19		
Winter 2019 008B^b^		
High Module (*n* = 113)	127 ± 36	3.32 ± 0.43
Low Module (*n* = 107)	77.8 ± 32.7	3.18 ± 0.50
Module Median = 16		
Winter 2023 008B^b^		
High Module (*n* = 115)	107 ± 42	3.44 ± 0.47
Low Module (*n* = 115)	65.3 ± 29.2	3.06 ± 0.41
Module Median = 17		

a Low module refers to the group of students with total module activity scores < module median score; high module refers to students with total module activity scores ≥ module median score; for the Fall 2018, Fall 2019, Winter 2018, and Winter 2023 courses, students at the median score were evenly distributed between the low and high module groups.

b The CHEM 001B cohorts consisted of a mix of students from the preceding CHEM 008A course and students who had different instructors in the prior course (the Winter 2018 course had 94 students from the Fall 2017 008A course; the Winter 2019 008B course had 84 students from the Fall 2018 course; the Winter 2023 008B course had 98 students from the Fall 2022 008A course).

Analysis of covariance (ANCOVA) was used to compare the final exam scores between the Low Module and High Module groups while including university GPA as a covariate. The assumptions for ANCOVA were met for all the courses except for the Fall 2022 CHEM 008A and Winter 2023 CHEM 008B courses (see SI Appendix 2, sections B–D, F, and G for summaries of the tests of assumptions and the raw output tables from the ANCOVA). Estimated marginal means were used to compare the final exam performance between the Low Module and High Module groups (see Table 2). For the Fall 2022 CHEM 008A and Winter 2023 CHEM 008B courses, the assumptions for ANCOVA were not met (see SI Appendix 2, sections E and H, respectively). Because the relationship between final exam score and university GPA did not appear to be equivalent across the Low Module and High Module groups, a multiple linear regression analysis that included a GPA-Module Score interaction term was carried out for these two courses as previously described.^19^ The tests for assumptions, raw output tables, and model summaries for these analyses are provided in SI Appendix 2 (sections 2E and 2H). The regression coefficients and model summaries are provided in Tables 3 and 4. The ANCOVA and multiple linear regression analyses were carried out using the IBM SPSS software program, version 28.0.0.0.

**Table 2:** Summary of ANCOVA Analyses^a^

Class/Study Group	Est. Marginal Mean of Final Exam (Std. Error)	95% Confidence Interval (Lower–Upper Bound)	*F*	*p*	Partial η^2^	Cohen’s *f* Effect Size
Fall 2017 008A						
High Module	116 (2.84)	111–122	65.9 (1, 191)	<0.001	0.258	0.590
Low Module	81.4 (3.14)	75.2–87.6				
Fall 2018 008A						
High Module	115 (2.89)	109–120	66.1 (1, 262)	<0.001	0.202	0.503
Low Module	81.0 (2.89)	75.3–86.7				
Fall 2019 008A						
High Module	120 (2.71)	115–126	77.3 (1, 262)	<0.001	0.229	0.545
Low Module	86.4 (2.71)	81.1–91.7				
Winter 2018 008B						
High Module	120 (3.56)	113–127	46.7 (1, 204)	<0.001	0.187	0.480
Low Module	85.5 (3.56)	78.5–92.6				
Winter 2019 008B						
High Module	126 (3.24)	120–133	105 (1, 218)	<0.001	0.327	0.697
Low Module	78.2 (3.33)	71.6–84.8				

a Dependent variable = final exam total score; covariate = university GPA; Cohen’s *f* = (η^2^/1 – η^2^)^1/2^. The high module and low module study groups were created as described in Table 1.

**Table 3:** Summary of Multiple Linear Regression for the Fall 2022 CHEM 008A Course^a^

Variables in Model	Unstandardized *B*	Std. Error	Standardized β	*t*	*p*
(constant)	–79.3	10.1		–7.83	<0.001
Module Score	2.07	0.316	0.312	6.56	<0.001
GPA	43.3	3.78	0.543	11.4	<0.001
GPA*Module Score	2.88	0.489	0.489	5.90	<0.001

a Model summary (dependent variable = final exam score): *R* = 0.799; *R*^2^ = 0.639; adjusted *R*^2^ = 0.634.

**Table 4:** Summary of Multiple Linear Regression for the Winter 2023 CHEM 008B Course^a^

Variables in Model	Unstandardized *B*	Std. Error	Standardized β	*t*	*p*
(constant)	–105.6	12.9		–8.17	<0.001
Module Score	2.39	0.44	0.296	5.48	<0.001
GPA	46.3	4.6	0.534	10.0	<0.001
GPA*Module Score	1.49	0.80	0.086	1.88	0.062

a Model summary (dependent variable = final exam score): *R* = 0.734; *R*^2^ = 0.531; adjusted *R*^2^ = 0.527.

A preliminary data analysis conducted in the fall of 2022 showed that performance on the in-class module activities was strongly correlated to the final exam performance. Because students who scored lower on the module activities scored significantly lower on the final exam, the Activity Engagement Survey (AcES) was administered in the Winter 2023 CHEM 008B course to help answer research question 2.^20^ The AcES instrument was administered to students after each in-class activity under the approved IRB protocol HS-22-198 (a detailed overview of how the surveys were administered to students is provided in SI Appendix 2A). Students were given extra credit on the in-class module activities for each survey they completed, and this extra credit was provided even if students did not consent to have their survey and class performance data included in the postcourse analysis. The survey instrument was administered using an online Qualtric interface, and students had 48 h to submit the survey after each in-class module activity. Since students’ engagement is expected to be malleable (i.e., varies depending on the environment),^21,22^ the scores and survey responses for each module were treated as independent, even though students were invited to participate in the survey for each module. Differences in the number of students who reported working with others versus working by themselves based on module score were investigated using a chi-squared test, which was calculated using the stats package in R (version 4.2.2). The effect size of any differences was determined by calculating Cohen’s *w* using the rcompanion package (version 2.4.30) in R. Guidelines for Cohen’s *w* suggest 0.1, 0.3, and 0.5 represent small, medium, and large effects, respectively.^23^

Data collected for the BC and E scales were separately analyzed using single-factor confirmatory factor analysis (CFA) with maximum likelihood estimation with Satorra-Bentler adjustment and robust standard errors.^24^ CFAs were tested using the Lavaan package (version 0.6.15) in R. Fit statistics for each scale suggested a reasonable to good data-model fit, which provided evidence of internal structure validity (see SI Appendix 2, section 2I). Evidence of single-administration reliability was found through calculating omega (see SI Appendix 2, section 2I)^25^ using the userfriendlyscience package (version 0.7.2) in R. A correlated BC-E AcES model with a negative method factor was tested.^26^ Fit statistics met the recommended cutoffs for reasonable to good fit (see SI Appendix 2, section 2I). The aggregated data set was split into groups by module score to explore possible engagement differences between students who received a low score (module score = one) and students who received a high score (module score = five). Before comparisons were completed, measurement invariance testing was conducted to provide evidence of consequential validity.^27^ Unweighted mean scores for behavioral/cognitive and emotional engagement were conducted using analysis of variance (ANOVA) using the lessR package (version 4.2.6) in R. Effect size was calculated using Cohen’s *f*, where 0.10, 0.25, and 0.40 represent small, medium, and large effects, respectively.^23^

## 3 Results

### 3.1 Analysis of Final Exam Performance

The total median module activity score for the entire quarter was used to create “high module” and “low module” study groups for each course included in the study. The module activity median scores, the mean final exam scores, and the mean university GPAs for the low and high module groups are summarized in Table 1. The descriptive statistics reveal that the high module group appeared to have higher final exam scores and higher university GPAs than the low module group in all courses. The total module activity scores and final exam scores were significantly correlated for all courses (Pearson’s *r* ranged from 0.520 to 0.649; *p* < 0.05), the final exam scores and university GPAs were significantly correlated for all courses except for CHEM 008A Fall 2019 (Pearson’s *r* for all other courses ranged from 0.152 to 0.717; *p* < 0.05), and the total module activity scores and university GPAs were significantly correlated for all courses (Pearson’s *r* ranged from 0.140 to 0.524; *p* < 0.05, see SI Appendix 2, sections 2B–H for all bivariate correlations).

Because university GPA was significantly and positively correlated to final exam score for all but one of the courses, ANCOVA was used to compare the mean final exam scores between the high and low module groups while including GPA as a covariate. As stated above in the Methods, the assumptions for ANCOVA were met for all the courses except for CHEM 008A Fall 2022 and CHEM 008B Winter 2023 (see SI Appendix 2, sections 2B–H). The estimated marginal mean final exam scores for the high and low module groups and test statistics for the remaining courses are summarized in Table 2. When accounting for the impact of university GPA on final exam score variance, the high module group scored higher on the final exam than the low module group in every course. The difference in marginal mean final exam score ranged from 34 to 48 points (*p* < 0.001), resulting in moderate to large effect sizes.

The data for the Fall 2022 CHEM 008A and Winter 2023 CHEM 008B courses did not meet the assumptions for ANCOVA (see SI Appendix 2 sections 2E and 2H), therefore multiple linear regression analyses were carried out to determine if overall in-class module scores were a predictor of final exam score for these two cohorts. A hierarchical analysis was carried out in which total module score and university GPA were first included as independent variables, and then a subsequent model was created that included total module score, university GPA, and a GPA*module score interaction as independent variables. This analysis indicated that change in *R*^2^ was significant upon adding the GPA*module score interaction term, hence this model was retained in the analysis (see SI Appendix 2 sections 2E and 2H). The results of these analyses for Fall 2022 CHEM 008A and Winter 2023 CHEM 008B are summarized in Tables 3 and 4. Module score, university GPA, and the GPA*module score interaction term are all significant predictors of final exam score, and these models explained 63.4% and 52.7% of the variance in final score for the Fall 2022 and Winter 2023 courses, respectively. The unstandardized *B* coefficient provides insight about how the module score impacts final exam score, while holding constant the other independent variables—for Fall 2022 CHEM 008A, a one-point increase in total module activity score resulted on average in a 2.07 point increase in final exam score (*p* < 0.001) and for Winter 2023 CHEM 008B, a one-point increase in total module activity score resulted on average in a 2.39 point increase in final exam score (*p* < 0.001). To provide more context, consider that the module activity scores ranged from 0 to 25 points (008A) or 0–20 points (008B), and the final exam from 0 to 200 points. If a student were to improve from 10 to 20 points on the in-class module activities, this would likely result in an approximately 20-point or 24-point increase in final exam score, respectively for CHEM 008A and 008B.

The GPA*module score interaction term suggests that university GPA modulates the total module score; therefore, it was of interest to determine how university GPA impacts final exam score across three levels of total module score. The students were divided into three groups based on module score (high module ∼ top third of module scores; medium module ∼ middle third of module scores; low module ∼ lower third of module scores, see SI Appendix 1 Section E and H for number of students assigned to each group) for both classes. When best-fit linear plots of final exam score vs university GPA were created across the three levels of module score, it was apparent that university GPA was less correlated to final exam score for the low module group in both the Fall 2022 and Winter 2023 courses (Fall 2022: *R*^2^ for high module = 0.550 and *R*^2^ for low module = 0.158; Winter 2023: *R*^2^ for high module = 0.421 and *R*^2^ for low module = 0.408, see SI Appendix 2 sections 2E and 2H). Though this difference appears to be less striking for the Winter 2023 course, these results suggest overall that even if students had higher incoming university GPA, lower performance on the module activities generally resulted in lower final exam scores. The results from these multiple linear regression analyses appear to parallel those from the ANCOVA, in which it was observed higher performance on the in-class module activities appears to be significantly associated with higher final exam scores.

### 3.2 AcES Survey

**Figure 2:**
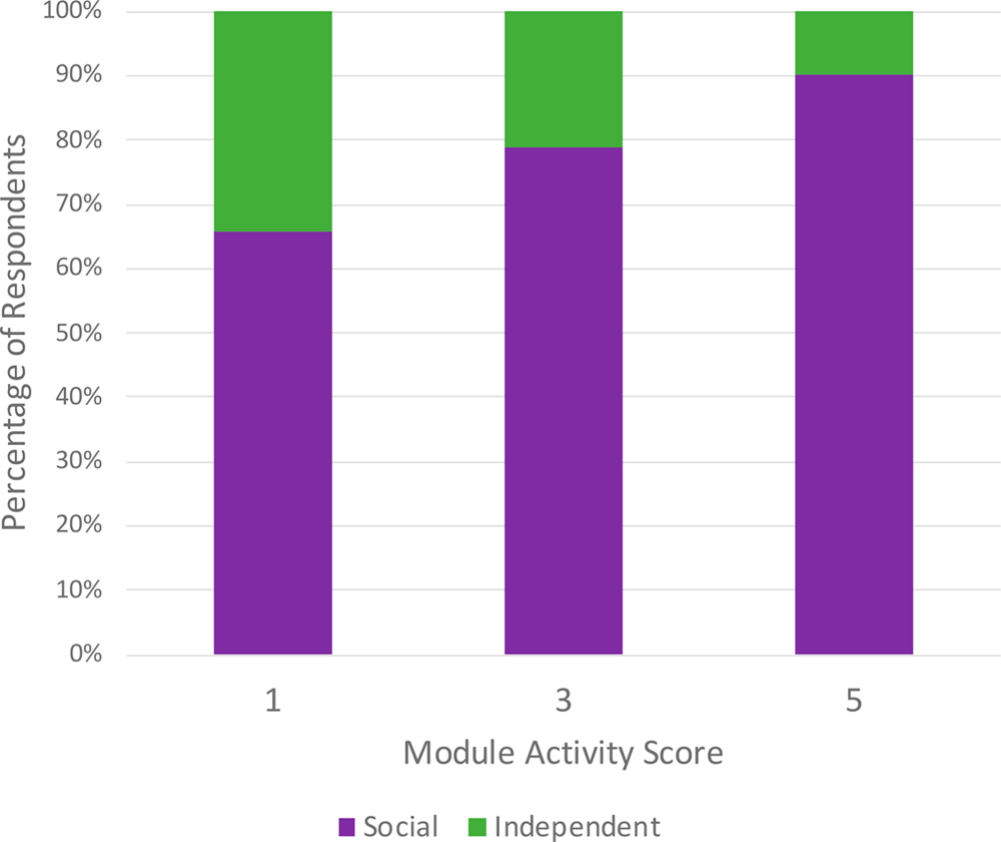
Percentage of respondents who indicated they worked with others (i.e., social) versus worked by themselves (i.e., independent) on an activity based on the module activity score of 1 (*n* = 111), 3 (*n* = 334), or 5 (*n* = 383).

A chi-squared test was used to determine if the proportion of students who chose to work with others (i.e., social) versus work by themselves (i.e., independent) was significantly different among students who scored one (*n* = 111), three (*n* = 334), or five (*n* = 383) on a module activity (Figure 2). Overall, a significant difference was found, χ^2^(2) = 39.287, *p* < 0.001. Students who scored higher on an activity were more likely to report working with others on that activity. Pairwise comparisons with a Bonferroni correction were conducted and all pairwise comparisons were found to be significant. The two pairwise comparisons between adjacent scores were found to represent small effect sizes (Cohen’s *w* = 0.13 and 0.15). The comparison between students who scored a one and students who scored a five was found to represent a medium effect (Cohen’s *w* = 0.28).

Students’ behavioral/cognitive (BC) and emotional (E) engagement were compared based on individual module activity scores using ANOVA. Only the responses from students who scored either one (“low score”) or five (“high score”) on a module were compared, as evidence was only found to support conservative invariance for this group comparison through measurement invariance testing (see SI Appendix 2, section 3I). Results showed that there was a significant difference in both the BC and E engagement between groups. Those who scored a five reported significantly higher BC and E engagement than those who scored a one, both with a medium effect size (Table 5).

**Table 5:** Observed Unweighted Mean Engagement Scores and ANOVA Comparison Results between a Low Score and a High Score on a Module Activity

	Low Score	High Score		
Scale	Mean (SD) (*n* = 111)	Mean (SD) (*n* = 383)	Mean Difference^a^	Effect Size (Cohen’s *f*)
BC	4.68 (0.72)	5.12 (0.66)	**0.44**	0.27
E	3.74 (0.98)	4.29 (0.90)	**0.55**	0.25

a Bold values indicate the difference was statistically significant at *p* < 0.001.

## 4 Discussion

### 4.1 Use of Flipped Classroom Structure to Facilitate In-Class Module Activities

The flipped aspect of the class was 2-fold: the active learning modules and the preclass videos. While the effect of the modules could be quantitatively assessed, the effect of the videos was more challenging to determine, as they did not cover material that was directly related to the learning modules but rather covered core “basic” topics in organic chemistry. However, the focus on basic, rewatchable topics had two major, obvious benefits—first, students could watch and rewatch the videos at their leisure throughout each quarter of the courses, to reinforce their basic understanding, which set the groundwork for tackling more complex problems. Instead of watching a video, answering questions on that video, and then performing a module about the video, these minilectures encompassed a far greater range of topics. There were many positive comments about this in the postclass student evaluations, as students found that multiple viewings made them more confident in their abilities. The second benefit was operational—the content of the learning modules could easily be altered and improved without having to refilm the corresponding videos.

In addition to the posted videos, premodule learning activities consisted of lectures on the relevant topic(s) (approximately 1–2 weeks before the module), and posted homework sets that mirrored the style of the modules and covered similar content. The problem sets were all posted at the start of the quarter, and detailed answers to the relevant problem sets were posted before the module date. The students were allowed to bring these answer keys into class during the modules so that they could refer to them.

It should be noted that watching the videos, while stated as required in the syllabus and in class, was not linked to class points, nor was policed in any way—the videos were provided to the students, but they chose whether to avail themselves of them. In the 2022–2023 year, viewing statistics for the videos were monitored, and approximately 60–80% of the class watched the videos all through at some point in the quarter. Interestingly, a significant portion of students who watched the videos watched them multiple times, but at least 20% of each class did not watch the videos at all. This level of student interaction with the preclass videos is roughly equivalent to the viewing statistics observed in a previous multicourse analysis of flipped classroom structures, though the percentage of students not viewing the content at all reported here appears to be higher than in this previous report (the percentage of students not viewing the content across the five courses in this previous study was less than 10%).^28^ This is possibly due to the fact that course credit was not directly awarded for completion of this preclass material (though students were informed that lack of compliance on the preclass assignments would likely negatively impact their performance on the in-class module activities).

The physical aspects of implementing the modules are important to discuss. The nature of the room posed some difficulties as there was minimal space to walk down the rows of seats. The room did have ample space to the sides of the seating, so congregations would form on the staircases, etc. The facilitators were instructed to ensure that the students asked questions and were guided through their thought process rather than simply giving the solution to the students. Overall, while the level of financially supported facilitators was low (one-half-time TA), it became quickly obvious during the pilot program (2016) that the modules required a facilitator presence and ran significantly more effectively with a larger volunteer facilitator cohort. A set of 6–8 facilitators (including the instructor) for a 250-person class was deemed the minimum level of staffing that allowed sufficiently rapid response to student questions. The facilitators were also personally selected by the instructor and were among the most accomplished graduate and undergraduate students at UCR: running these modules is not simple and does require a deal of attention from the Instructor.

### 4.2 Impact of Module Activities on Student Performance

The results from the ANCOVA and multiple linear regression analyses suggest that students who are successful at completing the in-class module activities have a significant advantage on the final exam, even after statistically accounting for the potential impact of incoming university GPA on course performance. For the Fall 2017–2019 CHEM 008A and Winter 2018–2019 CHEM 008B courses, the final exam estimated marginal means for the High Module group ranged from 33 to 47 points higher than the Low Module group (see Table 2), resulting in large Cohen’s *f* effect sizes for all these courses (Cohen’s f effect sizes: 0.10 = small; 0.25 = medium; 0.40 = large).^29^ For the Fall 2022 CHEM 008A and Winter 2023 CHEM 008B courses, the unstandardized *B* coefficients from the multiple linear regression analyses reveal that students’ overall module scores result in significant improvements in final exam performance while controlling for incoming university GPA (unstandardized *B* = 2.07 and 2.39 for Fall 2022 and Winter 2023, respectively, see Tables 4 and 5). In other words, for every point increase on the total module score, this results in a 2.07- or 2.39-point increase on the final exam for the Fall 2022 CHEM 008A and Winter 2023 CHEM 008B courses, respectively. Thus, if a student improved by 10 points on their total module score in these two courses, the model predicts this would result in a 20–24 point increase on their final exam (given the exam was worth 200 points total, a 20–24 point increase equates to an approximate increase of one letter grade; e.g., from a grade of C to B or from a grade of B to A).

**Figure 3:**
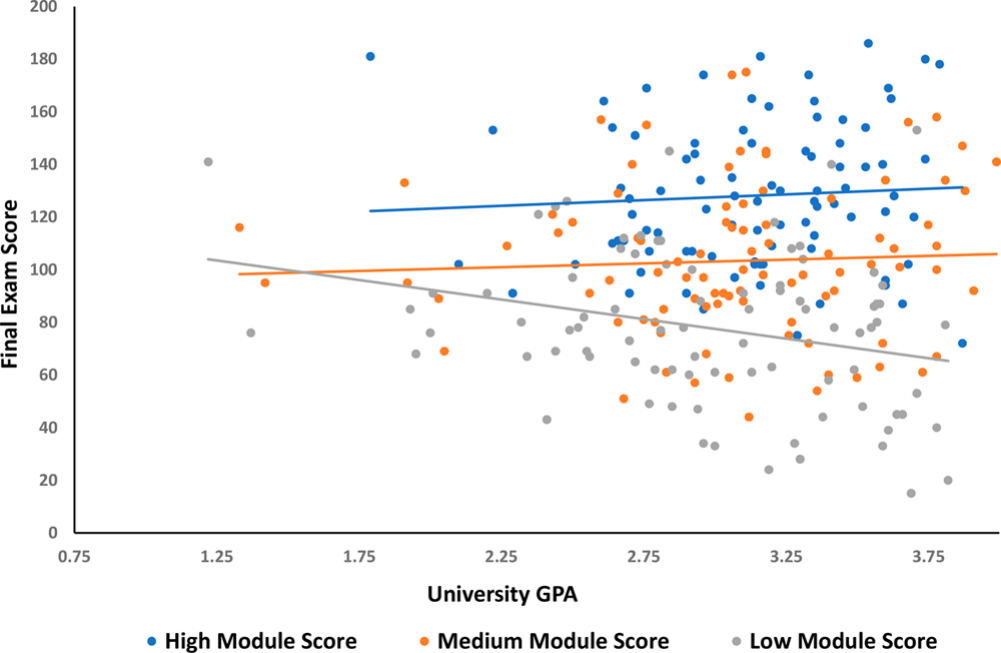
Example plot of final exam score vs university GPA across three levels of in-class module activity score (Fall 2019 CHEM 008A). The equivalent plots for all the courses included in the study can be found in SI Appendix 2 sections B–H.

To gain further insight about the potential impact of incoming university GPA as a potential confounding factor on final exam performance, final exam scores were plotted against university GPA across three levels of total module scores for all the courses included in this study (high module ∼ top third of module scores; medium module ∼ middle third of module scores; low module ∼ lower third of module scores; see Figure 3 for an example plot and see SI Appendix 1 Sections B–D, F–G for number of students assigned to each group and all plots of final exam score vs GPA). For all courses except Fall 2019 CHEM 008A and Winter 2018 CHEM 008B, university GPA was more weakly correlated to final exam score for the lowest tertile of students for total module score compared to the students in the highest tertile of total module scores. University GPA was more weakly correlated to final exam scores for the overall class populations for the Fall 2019 and Winter 2018 courses (see SI Appendix 1 Sections D and F); therefore, university GPA was also not strongly correlated with final exam score in the lowest tertile of module score in these two courses. Because students with higher university GPAs were observed to be in the lowest tertile of total module performance, these data suggest that module performance may have had an impact on final exam performance irrespective of incoming student academic performance. In other words, though university GPA was significantly correlated to final exam performance for the overall class populations (except for the Fall 2019 course), the fact GPA was less positively correlated to final exam performance for the lowest tertile of module performers suggests poor performance on the in-class activities may have been a significant contributor to poor exam performance.

These analyses provide insight about answering research question one, and there is a strong possibility that improved performance on the in-class module activities is linked to improved performance on the final exam. However, it is important to note that the post hoc observational nature of this study limits the ability to isolate the impact of the module activities outside the other cognitive and affective factors that may have impacted the final exam performance. For instance, students in the Low Module group may have had lower levels of overall engagement in the course (e.g., less time spent outside of class studying/engaging with course material), and students in the High Module group may have had higher levels of engagement and motivation. If this were the case, module performance may simply have been an artifact of broader course engagement and thus did not distinctly impact final exam performance (e.g., if broader course engagement and motivation were the variables that more directly impacted final exam performance, students in the High Module group may have performed better on the final exam even if the in-class activities were not included in the learning experience). Additionally, even if one assumes that incoming university GPA acts as a good proxy for overall engagement and motivation,^30^ the sophomore organic courses included in this study are considered by many students to be among the most difficult in the College of Natural and Agricultural Science (CNAS) at UCR. This negative reputation might result in a broader student population being less motivated, and therefore having lower engagement in these courses. Thus, it is possible that the university GPA may not accurately predict student engagement for the CHEM 008A and 008B courses included in this analysis.

To address this question further, there are some qualitative conclusions that can be drawn from discussions about the modules with students (undertaken by the instructor) and observations of their behavior in class (independent of the AcES survey discussed below). It was clear that student enthusiasm for the modules showed a strong link with both engagement in the class and performance—engaged students who performed well on the final were excited about the modules, whereas students who found the class more difficult regarded them as extra examinations, rather than additional opportunities for learning. Comments in the postclass evaluations contained examples of this, where some students felt “stressed” during the modules. There was some resistance to group learning, and many students preferred to work alone. Figure 2 reinforces this observation: more students who averaged lower scores on the modules preferred to work alone than those who were more successful. This may have been exacerbated by the room layout, which was not conducive to sitting together in groups.

There is another observation that is pertinent to the study. From the instructor’s point of view, the first module in each class provided an invaluable “shock” to the students and introduced them to the level of understanding required for the exams early in the class. While the scores in the first module were generally lower than those in the subsequent modules, the challenge presented by this module inspired more focus and dedication in many students, as evidenced by the statistical data described above. Organic chemistry is a challenge for many students, and providing a “wake-up call” early on in the class seems to be invaluable for focusing them on the class. The fact that there are minimal penalties for poor performance in the first module helps, in that the module can direct the students toward more focused learning without having a deleterious effect on their grade. The downside is that students may become discouraged with a poor score on the initial module (despite the fact that they are not grade-penalized for it), and this sets the tone for the class. However, the overall data showing that a majority of students were aided by the modules illustrate that this method provides an overall positive learning environment, as opposed to a traditional didactic lecture.

### 4.3 In-Class Module Activities and 3D Learning

Despite the limitations of this observational study, the observed relationship between the in-class activity module performance and final exam performance provides compelling evidence that instructors should strongly consider adopting these types of higher order learning activities. It is speculated here that the possible impact of the in-class module activities on final exam performance lies in the fact that these activities provide a low-stakes environment in which students can engage in the type of higher order thinking required on the final exam. As stated in the introduction, the in-class module activities were not created using the 3D learning framework, but a retrospective analysis reveals that there are indeed several elements of 3D learning present in these activities. There is some variance across the activities, but all of them connect to the *core ideas* of molecular structure and properties, include the *scientific practices* developing and using models and constructing explanations, and incorporate the *crosscutting concepts* cause and effect, structure and function, and stability and change (see SI Appendix 1 for the full in-class module activities and identification of 3D learning elements present therein).^3^ Our in-class module activities demonstrate how instructors can use a hybrid learning classroom to implement reform-minded instruction.

If the assumption is made that the performance on the in-class module activities has some impact on the final exam scores, the data reported here might corroborate recent findings that found students who were routinely required to construct explanations (and not just provide “right” or “wrong” answers) were more likely to demonstrate correct reasoning in summative 3D assessments.^31^ Because our in-class module activities required the same type of reasoning that was required in the final exams, students were routinely given opportunities to engage in this type of thinking and likely recognized that this form of higher order learning was valued by the instructor. It appears that at least for the students who were more engaged with the in-class module activities (i.e., the High Module students) this correlated to improved performance on final exam questions that required higher order thinking.

### 4.4 AcES Survey and Student Engagement

Given the apparent differences in final exam performance between the High Module and Low Module groups and the qualitative instructor reflections on the student response to these activities, it was important to gain more insight into the nature of the student engagement with the in-class module activities. As such, the AcES survey was administered as part of the Winter 2023 CHEM 008B course. To answer research question two, the analysis of the survey focused on comparing the behavioral/cognitive, and emotional domains of student engagement between students who scored higher (i.e., five) and students who scored lower (i.e., one) on a module activity. The survey also provided information about the proportion of students who worked in collaborative groups during the in-class module activities based on their module score.

A significant difference was found between the number of students who worked on the activity with others (i.e., social) and those who worked by themselves (i.e., independent) with respect to the module score achieved,. Overall, 90% of students who scored a five chose to work with other students, compared to 79% and 66% for students who scored three and one, respectively. This suggests that students who chose to work in groups were more likely to understand the material and perform well in the activity.

Overall, students who scored higher on a module activity (i.e., five) were found to report significantly higher behavioral/cognitive and emotional engagement in the activity than students who scored a one. A prior study using the AcES to investigate the activity-level engagement of general chemistry students found that students who reported higher behavioral/cognitive engagement scored higher on subsequent related exam questions, but emotional engagement was not found to be related to student exam scores.^20^ It is possible that this difference is due to the performance measure used; i.e., students who are more emotionally engaged may do better on the assignment in question (i.e., a module), whereas the activity-specific level of emotional engagement may not affect performance on an exam given weeks later.

### 4.5 How to Improve Student Engagement with In-Class Module Activities?

Given the possible connection between in-class module activity performance and final exam performance, the question becomes what changes can be made to the overall flipped classroom structure to increase student engagement in the in-class activities? As discussed above, one of the limitations of the in-class module activities implementation was the physical infrastructure of the classroom. Because the classes were held in a traditional auditorium lecture hall with fixed desks, it was difficult for students to work in collaborative groups. Conducting the in-class module activities in a room better structured to facilitate small group learning could help improve student social engagement.^32^ However, if this is not possible, then there are other mechanisms by which student engagement can possibly be improved.

Though scaffolding was built into the activities via use of the optional out-of-class homework problem sets, these assignments can be emphasized more by the instructor, and explicit callouts to the specific problem sets can be included at the beginning of the modules activities. Because the activities push the students to engage in more demanding conceptual understanding, helping students clearly recognize what pre-existing knowledge is required to complete the in-class modules might make them less overwhelming.

From a logistical standpoint, adjusting the point structure and nature of the in-class collaborative group work might also improve performance. The low grade point totals of the module activities helped strike a balance between incentivizing students to complete the activities while not inducing too much anxiety with respect to the fact that incorrect answers might adversely impact grades. Increasing the point values of the activities might help ensure students take them more seriously and improve compliance, although care needs to be taken to not create an exam-like atmosphere in which students simply focus on finding the “right answers” at the expense of engaging in meaningful learning. If the point values of the in-class module activities are adjusted, this might include awarding a small fraction of points for participating in collaborative groups (e.g., students might receive 1–2 points for having their group members sign the activity sheet and confirm they indeed worked in a group). As the AcES survey showed that students who worked individually received lower module scores, incentivizing students to work in groups and communicating to students the benefits of working with peers may have a positive impact.

Finally, finding ways to improve intrinsic motivation may be important. As stated above, these lower division organic chemistry courses are likely some of the most difficult courses in undergraduate STEM pathways. Moreover, many of these students are taking these courses to fulfill requirements for their major and/or for prehealth postbaccalaureate programs, which foster more *extrinsic* motivation and/or goal-oriented outcomes. One way to increase intrinsic motivation might be to incorporate issues of social importance and/or include applications to biological systems (e.g., environmental sustainability, green energy, environmental justice, disease and therapeutic mechanisms, etc.). If students clearly see how this type of higher order learning is required to address these real-world problems and/or problems relevant to their field of study (e.g., life sciences), they may cease to view this type of work as a hurdle to their success. For instance, using the United Nations Sustainable Development Goals to frame general chemistry learning outcomes is an example of improving affective outcomes.^33^ This type of thematic framework might improve student buy-in for a more cognitively demanding curriculum. Future work will involve using the AcES instrument to track changes in student engagement after these adjustments. This work might also involve formally tracking student buy-in using the exposure–persuasion–identification–commitment (EPIC) model, which has been used to analyze the connections between student buy-in, self-regulated learning, and course performance.^34^

### 4.6 Limitations and Conclusions

As described above, the primary limitation of this study lies in the observational nature of the analysis. Not only was it impossible to disaggregate student performance on the in-class module activities from broader course engagement, but it was also possible that other peripheral confounding factors may have contributed to the disparate outcomes for the low- and high-module groups. These include disparities in out-of-class cocurricular/extracurricular commitments, variance in the difficulty of overall course schedules, and/or differences in the perception of the value of organic chemistry in the students’ broader educational pathway. If there were indeed differences between the High and Low Module groups in any of these other characteristics, this could have contributed to the observed differences in the final exam performance.

Despite these limitations, the correlation between student performance on the in-class module activities and final exam performance is striking. After statistically controlling for university GPA, a likely proxy for general student engagement, students who scored above the median on the total in-class module activities scored 30–40 points higher on the final exam in five of the seven classes included in this study, and in the other two classes, the multiple linear regression models predicted a 10-point increase on the total in-class module activities that resulted on average in a 20–24 point increase on the final exam. Additionally, the observation that the Low Module groups were populated in part by students who had higher overall university GPAs suggests that there is an opportunity to improve course outcomes for a broader population of students. These results suggest that a flipped classroom structure can promote higher order learning outcomes in large enrollment undergraduate organic chemistry courses and broadening the impact of the in-class module activities might be achieved by improving student emotional and/or social engagement during the in-class activities. Finally, instructors wishing to implement instructional interventions similar to these are encouraged to provide opportunities for their students to practice higher order thinking and match their assessments accordingly, because if students cannot clearly see that this type of complex reasoning is valued by the instructor, they are likely to perform worse on summative assessments that probe three-dimensional learning.^31^ Though the in-class module activities show promise in helping students navigate more 3D learning, the findings from this study also suggest that many students may not successfully engage in this type of higher order learning. Classroom practitioners therefore need to make efforts to generate student buy-in and ultimately improve student outcomes.

## Supporting Information Available

Appendix 1: All in-class module activities and answer keys (PDF)
Appendix 2: Detailed descriptions of the tests of assumptions for the ANCOVA and multiple linear regression analyses, and complete data summaries for these analyses and the AcES survey analysis (PDF)
Example final exam for CHEM 008A (PDF)
Example final exam for CHEM 008B (PDF)
