# Supplementary material for: Using Flipped Classroom Modules to Facilitate Higher Order Learning in Undergraduate Organic Chemistry
Source: J Chem Educ. 2024 Jan 25;101(2):490–500. doi: 10.1021/acs.jchemed.3c00907 (PMC10867829; doi:10.1021/acs.jchemed.3c00907)
Supplement: Supplementary file 1 — ed3c00907_si_001.pdf [file ed3c00907_si_001.pdf]

Supporting Information – Appendix 1

Using Flipped Classroom Modules to Facilitate Higher Order Learning in Undergraduate Organic Chemistry

Lauren R. Holloway[a], Tabitha F. Miller[a], Bryce da Camara[a], Paul M. Bogie[a], Briana L. Hickey[a], Angie L. Lopez[a], Jiho Ahn, Eric Dao[a], Nicole Naibert[b], Jack Barbera[c], Richard J. Hooley[a]*, Jack F. Eichler[a]*

[a]Department of Chemistry, University of California-Riverside; Riverside, CA, 92521, USA.
[b]Portland Community College; Portland, OR, 97201
[c]Portland State University; Portland, OR, 97280

*E-mail: richard.hooley@ucr.edu; jack.eichler@ucr.edu

Table of Contents

A. Detailed Implementation Notes…………………………………………………………*S-2*

B. 008A In-class Module Activity 1……………………………………………………….*S-4*

C. 008A In-class Module Activity 2……………………………………………………….*S-5*

D. 008A In-class Module Activity 3……………………………………………………….*S-6*

E. 008A In-class Module Activity 4……………………………………………………….*S-7*

F. 008A In-class Module Activity 5……………………………………………………….*S-8*

G. 008A In-class Module Activity 6……………………………………………………….*S-9*

H. 008B In-class Module Activity 1……………………………………………………….*S-10*

I. 008B In-class Module Activity 2……………………………………………………….*S-12*

J. 008B In-class Module Activity 3……………………………………………………….*S-13*

K. 008B In-class Module Activity 4……………………………………………………….*S-14*

L. 008B In-class Module Activity 5……………………………………………………….*S-15*

A. Detailed Classroom Implementation

The modules were applied in the first two courses of the sophomore organic chemistry series at UC Riverside in the Fall and Winter quarters (these classes will be identified henceforth as Chem 008A and Chem 008B – the three-quarter Chem 008A/B/C series is equivalent to a standard two-semester organic chemistry series). The modules were piloted in 2016, and collection of student performance data was initiated in the fall of 2017. Seven quarters were assessed (based on when RJH was assigned to teach): first quarter Chem 008A was assessed in Fall 2017, 2018, 2019 and 2022, and second quarter Chem 008B was assessed in Winter 2018, 2019 and 2023 (the classes in 2020 and 2021 were interrupted by the COVID shutdown and therefore not included in this analysis). Because there are typically 3-5 sections of CHEM 008A and CHEM 008B offered each quarter, students do not progress from 008A to 008B as a cohort; the percentage of students who took CHEM 008A and 008B consecutively in the flipped format with instructor RJH ranged from 38-46% (see Table 1). The topic coverage spanned two-thirds of a standard organic chemistry curriculum (based in large part on the Solomons, Fryhle and Snyder textbook, 12th Ed)[17] and included structure and bonding (including alkane/cycloalkane conformations); stereochemistry; reactions and structure of alkenes and alkynes; nucleophilic substitution and elimination reactions; radical reactions; spectroscopy; conjugated dienes; aromaticity and reactions of aromatic compounds; reactions of alcohols, reactions and structure of carbonyl groups; retrosynthetic analysis.

The class enrollments ranged from 210-275 students, and each class was taught in an identical room layout, consisting of 288 seats in a standard auditorium setup (15 x 20 rows, with some blocks assigned for A/V equipment). The student population was predominantly life science majors (~70%), with the rest consisting of chemistry majors (~10%) and other engineering/physical science disciplines. The demographics of the student population were ~50% underrepresented minorities, and ~50% female, in line with the enrollment demographics in the UCR College of Natural and Agricultural Sciences (underrepresented minorities are classified at UCR as students who do not identify as white or Asian).[18]

Outside the in-class module activities, each class was taught as a standard lecture, with three 50-minute classes per week for ten weeks. Two midterms (100 points each) and a final exam (200 points) were assigned in all quarters. Between seven and nine homework worksheets were assigned, consisting of 10-18 multipart questions similar to the midterm/final questions (and some shorter introductory questions): these were not handed in or graded, but detailed multipage answer keys were provided after a cutoff date.

The in-class module activities were implemented in tandem with pre-class activities, which typically consisted of five 12-15 minute videos assigned for mandatory viewing by the class. These did not directly relate to the material on the module activities, but were designed to cover basic concepts in organic chemistry (stereochemistry, S_N2 mechanisms, NMR spectroscopy, electrophilic aromatic substitution, etc.) that students could rewatch at their leisure to reinforce those core concepts. The class time saved by the assignment of these videos was used for the in-class active learning modules. Either six (Chem 008A) or five (Chem 008B) module activities were implemented in each quarter. The single-sheet activities were handed out to the class, and the students had 25 minutes to perform the module. The module activity sheets were handed in at the end of class, scanned and graded.

During the module activities, students were encouraged to work in ad-hoc collaborative groups. A rotating array of volunteer teaching assistants (undergraduate supplementary instruction leaders, and volunteer graduate/undergraduate student TAs), the assigned head teaching assistant (TA) for

the class, and the course instructor (author RJH) were available in the room to answer any questions. On average, six facilitators were present for each module. The explicit answers were not given, but the facilitators were encouraged to help the students with their thought processes throughout. The students were allowed to use any materials, including notes/textbook or any online resources.

The module activities were graded out of five points each, and were assigned on a "count five, drop one" basis, so the lowest module score was removed from the grade. The total module activity points were 25 in Chem 008A and 20 in Chem 008B, out of 425 or 420 total points for the course, respectively. Final course grades were assigned via class ranking, with the mean point score being assigned a B-, and the D cutoff being one standard deviation below the mean. As such, the modules counted for ~6% of the total class score. It is also noted that from 2017 – 2019, the modules were graded from 0-5 points with each sub-question being assigned a specific point value. In 2022-2023, because of a reduction in TA support, the grading was simplified, and scores were assigned as: five points = broadly correct (defined as getting the majority of the concepts correct, with some leeway for small errors), three points = partially correct (some correct answers, but at least one large conceptual error), or one point = mainly incorrect (more than one large conceptual error). All the module activities were graded by a single TA. The assigned TA varied from quarter to quarter, but in each quarter, all ~1300 modules were graded by the same person to remove any variability in grading.

The final exams were graded by a team of TAs, and where possible each question in the midterm/final was graded by one TA. In some cases, the question was split between two TAs, but all grading was supervised in person by author RJH (who also graded multiple questions). The course syllabi, in-class module activity sheets, detailed keys and scoring rubrics for the module activities, final exams, and final exam keys and rubrics are provided in the SI. Approximately 50% of the final exam questions probe some combination of 3D learning aspects (e.g., structure-function relationships, constructing explanations, evaluating information, etc.). The exams are all written response/short answer questions with no multiple-choice questions. Some questions require a choice between four or five possibilities but are always paired with a follow-up "explain why" question. In addition, while retrosynthetic analysis, unknown chemical identification and mechanism questions do not match up exactly with the definition of 3D learning, these are the most challenging questions in the class that require the deepest thought and puzzle-solving attributes: if these are included as 3D assessment, over 80% of the questions in the final match with the type of question given in the modules.

B: 008A Learning Module 1: Acidity and Resonance

Question 1. In this module, we will focus on N,N-dimethylguanidine. Draw any missing ***lone pairs*** in the structure below and identify the ***hybridization*** of each of the 4 atoms denoted by an arrow.

Core Ideas: molecular structure & properties; change & stability in chemical systems

Scientific Practices: developing & using models; constructing explanations; engaging in argument from evidence

Crosscutting Concepts: cause & effect (mechanism & explanation); energy & matter; structure & function; stability & change

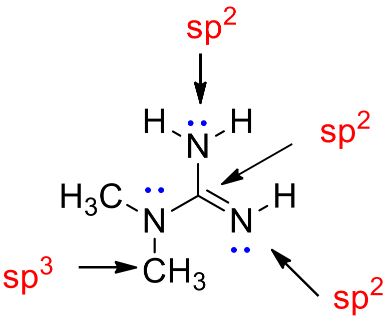

Question 2. Consider the reaction of N,N-dimethylguanidine with NaOH. There are two possible hydrogens that can be removed by NaOH, giving either anions ***A*** or ***B***. Draw ***arrow-pushing mechanisms*** for the reactions. For each reaction, label the ACID, and label the BASE.

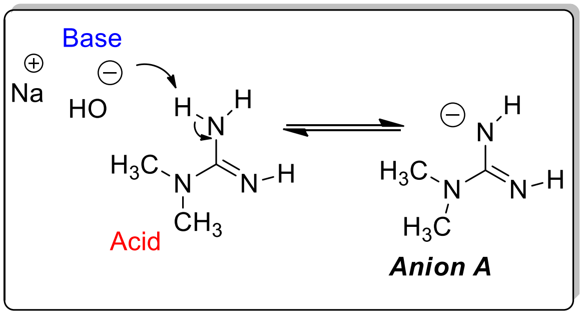

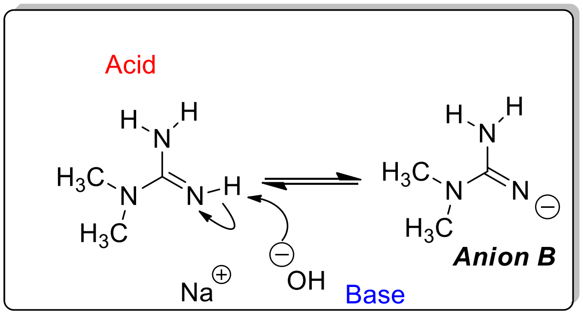

Question 3. Anion **A** is the most stable, and is the only product formed in the reaction. Draw any other *stable* resonance structure of anion **A**, and include arrows to show electron movement. Use this to ***explain why*** anion **A** is more stable than anion **B.**

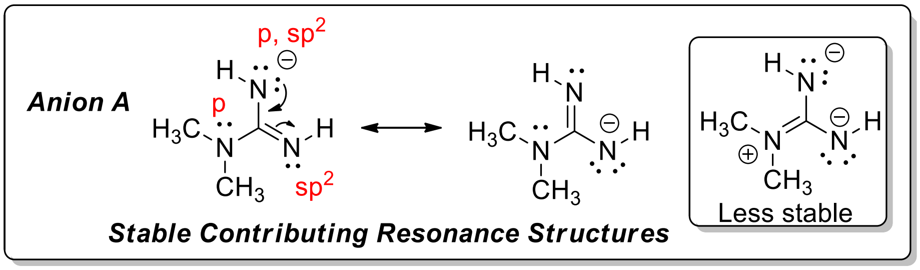

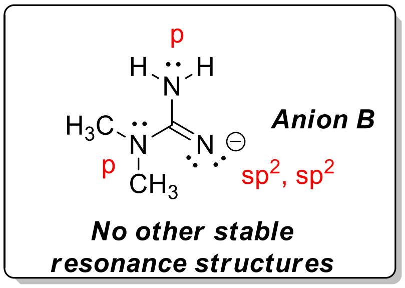

The negative charged is delocalized across two atoms in anion A. Anion B has no stable resonance structures, and so is less stable.

C: 008A Learning Module 2: Alkane Conformations

Question 1. In this module, we will look at the conformation of 2-fluorobutane. Draw the structure of three different conformations A-C in the energy diagram ***as NEWMAN projections.***

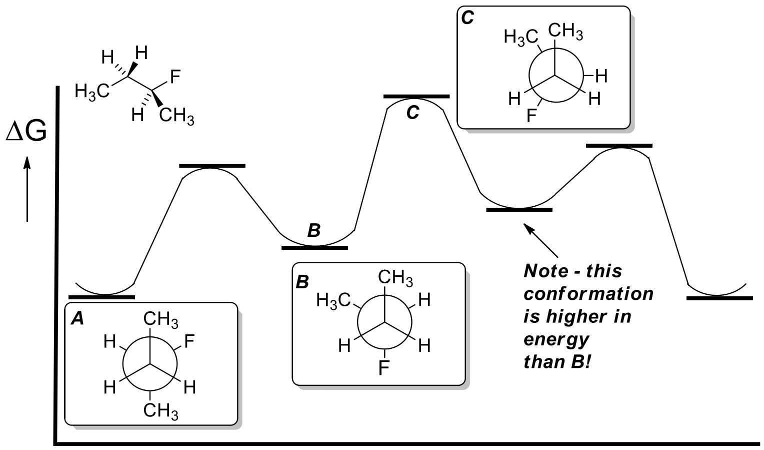

Core Ideas: molecular structure & properties; change & stability in chemical systems; energy

Scientific Practices: developing & using models; constructing explanations; engaging in argument from evidence

Crosscutting Concepts: cause & effect (mechanism & explanation); energy & matter; structure & function; stability & change

Question 2. (1 point) Which conformation (A, B or C) is ***LOWEST*** in energy? ***Explain why.***

A is lowest. The largest two functional groups (CH_3 and CH_3) are positioned anti to each other, and the molecule is in the staggered conformation. All eclipsed conformations are higher in energy, and the other two staggered conformations (with the CH_3 groups gauche, i.e. B, and with all three non-H groups gauche to each other) have greater torsional strain, as the larger groups are gauche to each other.

Question 3. (2 points) Draw the two possible chair conformations of molecule **1**, ***including ALL hydrogens.***

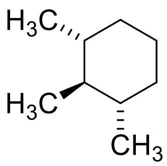

1

Chair Conformation 1:

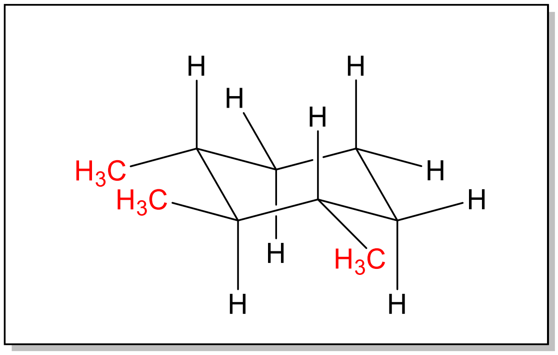

Chair Conformation 2:

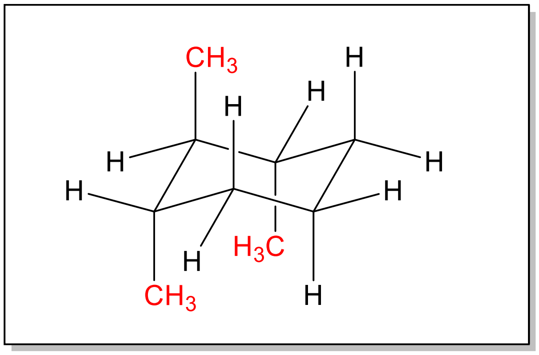

Which conformation is ***LOWEST*** in energy? ***Explain why.***

Conformation 1 is more stable as all the CH_3 groups are equatorial: in conformation 2, there are three axial CH_3 groups which have torsional strain with the cyclohexane ring. In addition, there is a 1,3 diaxial interaction between two of the CH_3 groups in 2, which is unfavorable.

D: 008A Learning Module 3: Stereochemistry

In this module, we will look at stereochemistry and isomerism. We will focus molecule **A**, below.

Question 1. How many stereocenters are present in molecule **A**?

Three (indicated by the stars to the right)

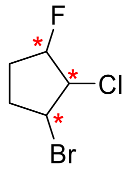

molecule A (no stereochemistry assigned)

Question 2. Draw two isomers of molecule **A** that have an ***enantiomeric*** relationship to each other. Draw two additional, different isomers of molecule **A** that have a ***diastereomeric*** relationship to each other. Make sure you label the relationships between the isomers.

You have a number of options here – enantiomers must be mirror images, i.e. ALL of the stereocenters must have different configurations (i.e. R or S). Diastereomers must NOT be mirror images, and NOT identical, so having one or two different configurations, but not all. There are lots of isomers here (2^3 = 8), so lots of possibilities. *Note - I haven't shown all of them!*

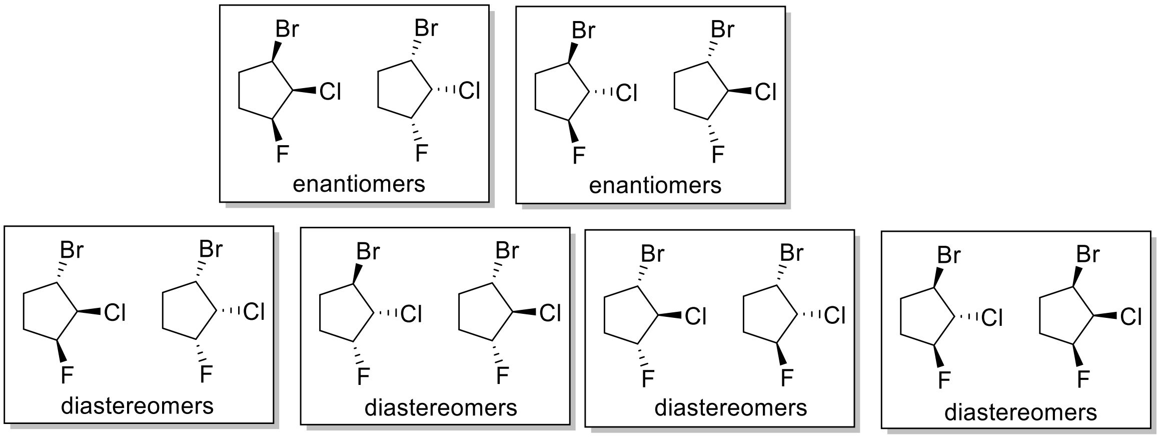

Question 3. One of the enantiomers of molecule **A** is shown. Identify the configuration (***R*** or ***S***) of each stereocenter in this molecule, as drawn.

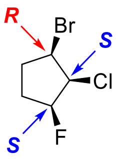

Question 4. Draw the isomer of molecule **A** where each stereocenter is in the ***R*** configuration.
Remember, there are only two possible configurations – if it's not R, it's S!

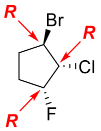

Core Ideas: molecular structure & properties<br><br>Scientific Practices: developing & using models<br><br>Crosscutting Concepts: patterns; structure & function

E: 008A Learning Module 4: Alkene Reactivity

In this module, we will look at alkene reactivity and its effects on stereochemistry. When molecule **A** reacts with HBr, two products are formed, **C** and **D.**

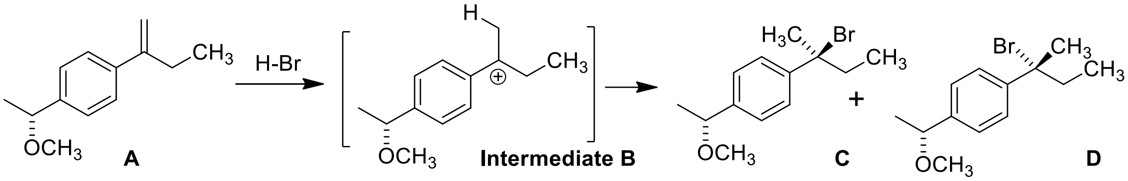

Question 1. Draw the arrow pushing mechanism for the synthesis of **C**. The intermediates and reactants are drawn below - just fill in the arrows. In each step, identify the ***nucleophile (Nu)*** and ***electrophile (E)***.

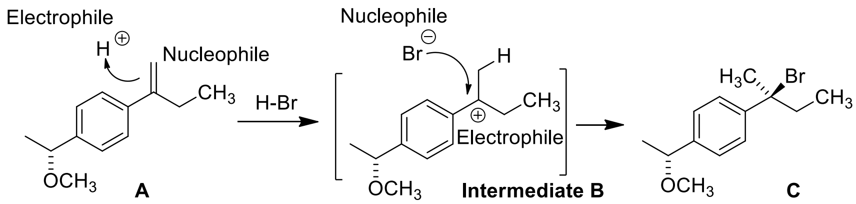

*The intermediate **B** cation is formed because that cation is stabilized by resonance. If the H+ added at the other carbon, there would be an sp^3 carbon between the cation and the benzene ring, preventing resonance and raising the energy of the intermediate.*

Question 2. Draw three additional stable resonance structures of intermediate **B**. Make sure you show the arrow pushing mechanisms to get to each resonance structure. Use this to explain why intermediate **B** is the product of the H$^+$ addition to **A**? (i.e. why does the H$^+$ not add at the other carbon in the alkene?)

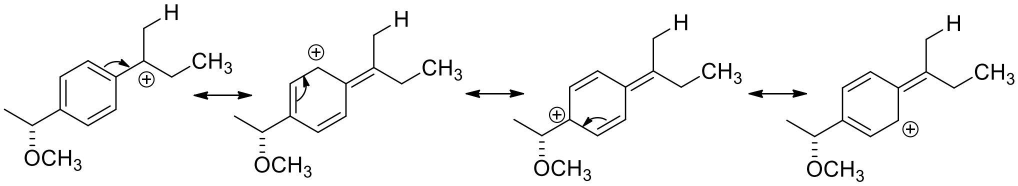

The intermediate B cation is formed because that cation is stabilized by resonance. If the H+ added at the other carbon, there would be an sp^3 carbon between the cation and the benzene ring, preventing resonance and raising the energy of the intermediate.

Question 3. What is the stereochemical relationship between **C** and **D?** ***Explain why*** both products are formed is almost equal amounts in this reaction.

***C** and **D** are **diastereomers.** The two compounds are non-superimposable, but they are not mirror images of one another. Both products are formed in equal amounts because the carbocation intermediate is planar, therefore the Br$^-$ can attack from either side.*

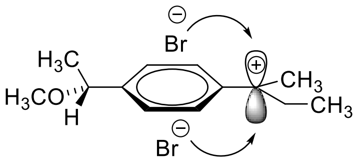

Core Ideas: molecular structure & properties; change & stability in chemical systems; energy

Scientific Practices: developing & using models; constructing explanations; engaging in argument from evidence

Crosscutting Concepts: cause & effect (mechanism & explanation; energy & matter; structure & function; stability & change

F: 008A Learning Module 5: Nucleophilic Substitutions

Question 1. In this module, we will look at controlling the outcome of substitution reactions. When molecule **A** is reacted with NaCN, only one product is formed. Draw the structure of the **TRANSITION STATE** of the reaction, and state the prevailing mechanism. ***Explain why*** this mechanism is favored.

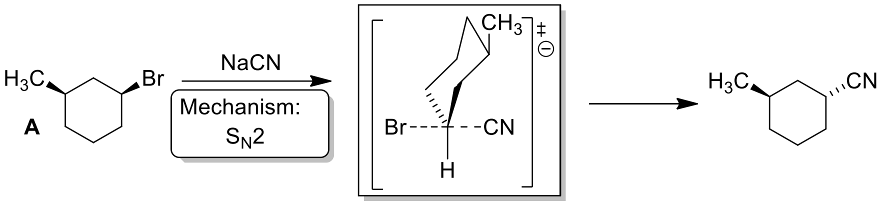

*$^-$CN is a **strong nucleophile and a weak base** which will react quickly with the electrophile (even though the electrophile is secondary and can, in theory, react via either mechanism). No cationic intermediate will be formed, so the pathway will be S_N2.*

Question 2. When **A** is reacted with $AgNO_3$ in water, two products are formed. Draw the structure of the **INTERMEDIATES** of the reaction, and state the prevailing mechanism. Draw an arrow-pushing mechanism for the process.

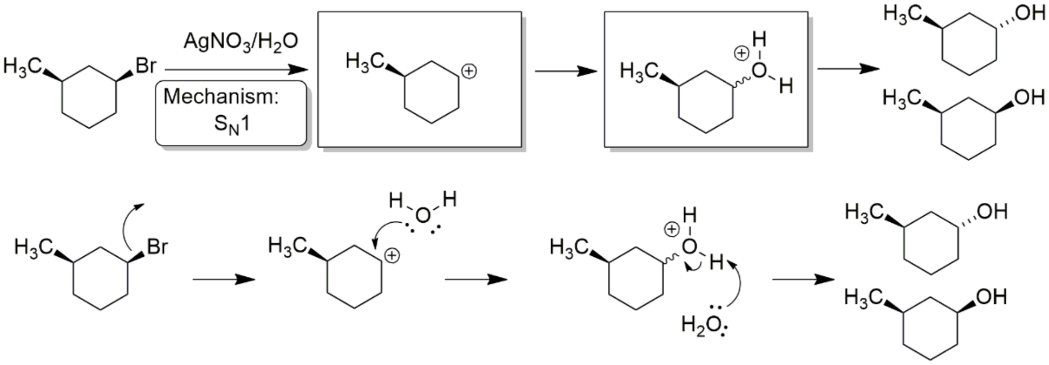

Question 3. In contrast, when **B** is reacted with $AgNO_3$ in water, FOUR products are formed. ***Explain why***. You will need to draw arrow-pushing mechanisms for the formation of ALL FOUR products.

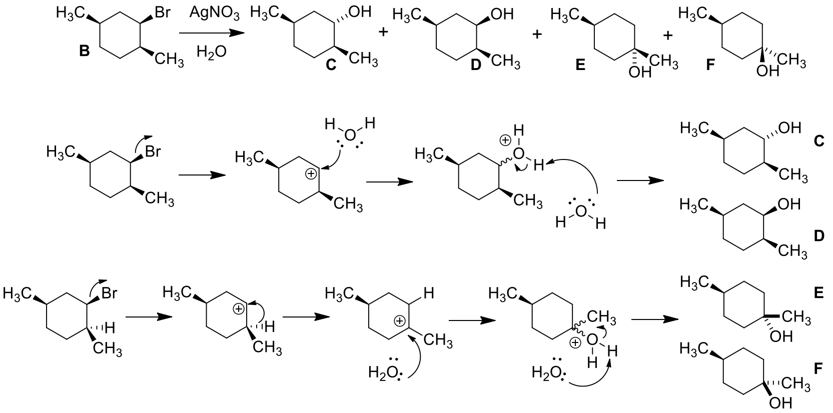

Core Ideas: molecular structure & properties; change & stability in chemical systems

Scientific Practices: developing & using models; constructing explanations; engaging in argument from evidence

Crosscutting Concepts: cause & effect (mechanism & explanation; structure & function; stability & change

G: 008A Learning Module 6: Substitution vs Elimination

In this module, we will look at controlling substitution vs elimination when both outcomes are possible. Reactant **A** has a variety of different reaction outcomes under different conditions. When reacted with aqueous silver nitrate, the reaction is, shall we say, ineffective, and 4 different products are formed.

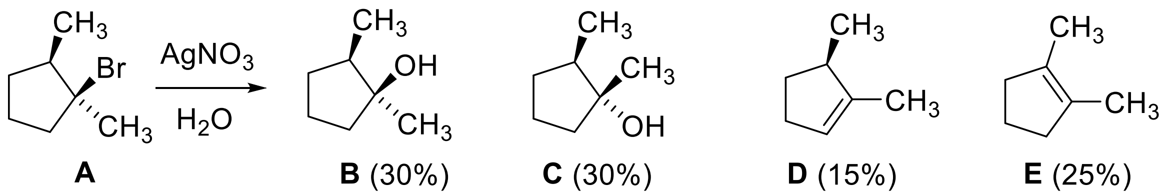

Question 1. Two mechanisms are occurring above - what are they? Explain WHY those mechanisms occur.

A combination of S_n1 and E_1 mechanisms are occurring (silver salt forces the formation of the carbocation and water is a weak base/poor nucleophile so no S_n2 or E_2 occurs). B and C are formed through the simple S_n1 mechanism and form an equal distribution of the two possible products. D and E are formed through the simple E_1 mechanism – mix of favored, less favored alkenes are formed (more substituted alkene formed in greatest yield).

Question 2. Draw the arrow-pushing mechanism for the formation of ***BOTH*** **C** and **E**.

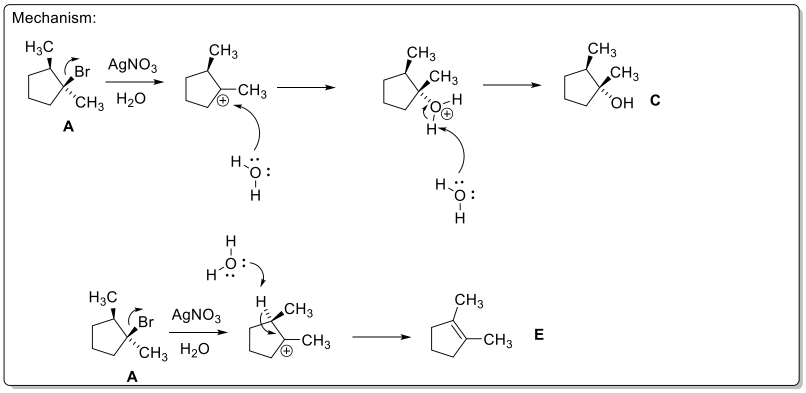

Question 3. Explain why **D** and **E** are formed in different yields, and explain why **E** is formed in greater yield than **D.**

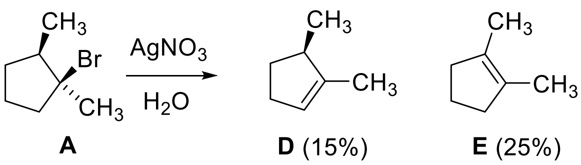

E is the most substituted alkene, so is formed in greatest yield. BUT – there's not much difference in energy between them, so you get both products.

Core Ideas: molecular structure & properties; change & stability in chemical systems; energy

Scientific Practices: developing & using models; constructing explanations; engaging in argument from evidence

Crosscutting Concepts: cause & effect (mechanism & explanation; structure & function; stability & change

<u>H: 008B Learning Module 1: Spectroscopy Key</u>

Consider the four isomeric molecules **A-D.**

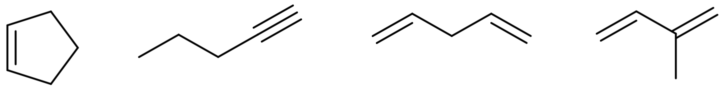

A **B** **C** **D**

Question 1. Could you distinguish between the four molecules by mass spectrometry? Give a one sentence reason for your answer.

No - they all have the same formula. Fragmentation patterns will be different, but not sufficient for an unambiguous assignment.

Question 2. Could you distinguish between the four molecules by IR spectroscopy? If so, what differences would you look for?

Yes - B will display C≡C and sp C-H stretches. D will display low frequency C=C stretches due to resonance. A and C will be difficult to distinguish.

Question 3. Could you distinguish between the four molecules by ^{13}C NMR spectroscopy? If so, what differences would you look for?

Yes - A will show 3 peaks due to symmetry, C will show 3 (with 2 peaks in the C=C region), D will show 5, and B will show C≡C peaks.

Question 4. Molecule **D** can be reacted with one molar equivalent of H-Br, to give an unknown molecule **E**. Use the spectroscopic information and your knowledge of alkene reactions to determine the structure. For full points, ***explain why*** you chose your answer from other isomeric possibilities.

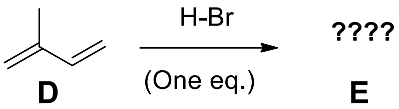

Spectral data for E:

MS (EI): *m/z* 150 (98%), 148 (100%), 69 (37%).

IR (cm^{-1}): 3112 (m), 2958 (s), 1645 (m)

^{1}H NMR (400 MHz, $CDCl_3$): δ 5.99 (1H, dd, J = 16, 10 Hz), 5.33 (1H, dd, J = 10, 2 Hz), 5.16 (1H, dd, J = 16, 2 Hz), 1.94 (6H, s).

^{13}C NMR (100 MHz, $CDCl_3$): δ 142.0, 112.6, 43.3, 38.2

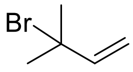

E

No symmetry in the other possibilities

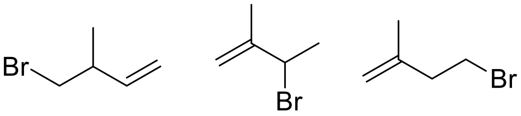

The key here is to rule out all the other possibilities, and look for the simplest answer. There are only 4 peaks in the ^{13}C spectrum, so there must be symmetry. Also, there is a 6H singlet in the ^{1}H spectrum - this can only happen if there are 2 CH_3 groups. All the other possibilities don't fit - there are too few alkene peaks, too many alkyl peaks, etc. E has a monosubstituted double bond and 2 CH_3. That's it.

IMPORTANT - focus on exactly what the question is asking. You don't need to fully analyze the spectra (which is hard), just rule out impossibilities.

Core Ideas: molecular structure & properties; change & stability in chemical systems

Scientific Practices: developing & using models; constructing explanations; engaging in argument from evidence; analyzing & interpreting data

Crosscutting Concepts: cause & effect (mechanism & explanation; structure & function

I: 008B Learning Module 2: Dienes and the Diels-Alder Reaction

Question 1. (2 points) Draw the arrow-pushing mechanism for the Diels-Alder cycloaddition reaction between cyclopentadiene and maleic anhydride. There are two possible isomeric products, the *exo* and *endo* isomers. Draw (in 3D!) the structures of these two products.

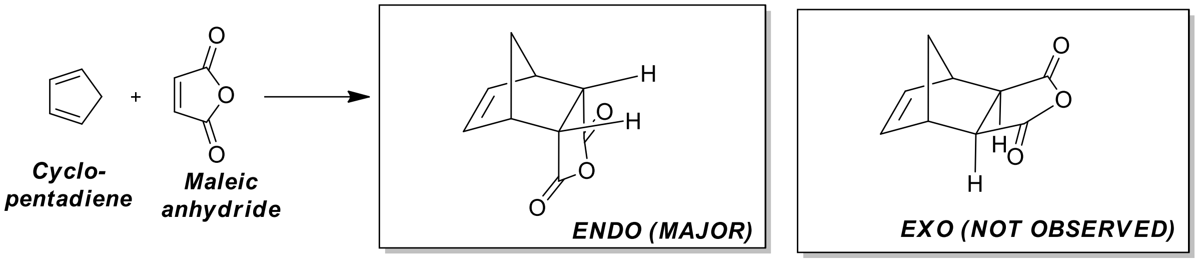

Question 2. (1 point) The endo *product* of the above reaction is actually HIGHER in energy (i.e. less stable!) than the exo product: however, the endo isomer is the only product observed in the reaction. Explain why this occurs.

Secondary orbital overlap (below) stabilizes the transition state of the endo reaction. The reaction is kinetically controlled, and does not equilibrate, so only the kinetic product (endo) is seen.

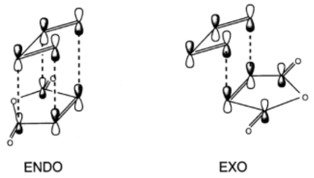

Core Ideas: molecular structure & properties; change & stability in chemical systems; energy

Scientific Practices: developing & using models; constructing explanations; engaging in argument from evidence

Crosscutting Concepts: cause & effect (mechanism & explanation; energy & matter; structure & function; stability & change

Question 3. (2 points). If the process is repeated with furan, the outcome is different. The reaction occurs at room temperature. After 10 mins, the ratio of endo: exo products is 80:20 (i.e. more ***endo***). After 24 mins reaction, the ratio of endo:exo is 50:50. After 50 mins, only the ***exo*** product is observed. Explain why. (HINT - furan is "aromatic" (see Ch 14), and is therefore significantly more stable than cyclopentadiene).

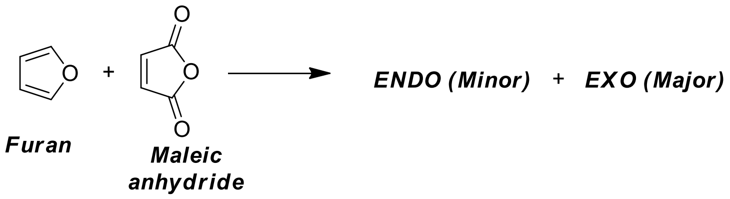

*If furan is more stable than cyclopentadiene, then the reverse Diels-Alder reaction can happen more quickly. After 10 mins, you get the **kinetic** product (endo). As the time increases, **equilibration** occurs, and the product is the **thermodynamic** one (exo).*

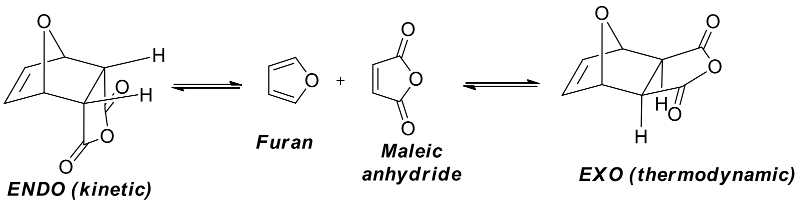

J: 008B Learning Module 3: Aromatic Reactivity

Question 1. Draw the arrow-pushing mechanism for the following reaction. Two products are formed, but you only need to draw the mechanism for the formation of the PARA product.

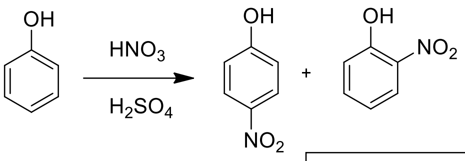

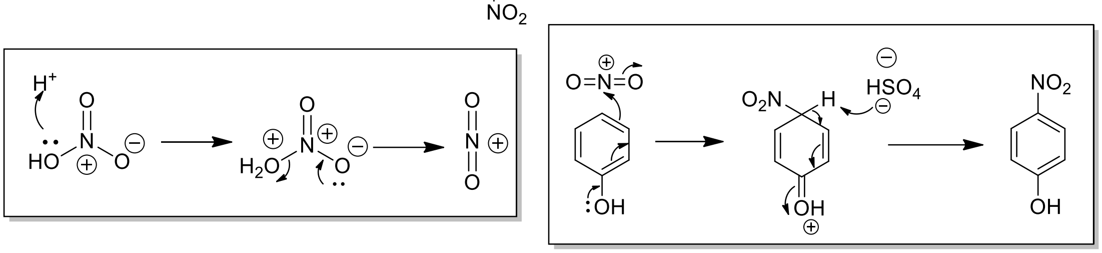

Question 2. Explain why the ortho and para products are formed, but not the meta (draw resonance structures!).

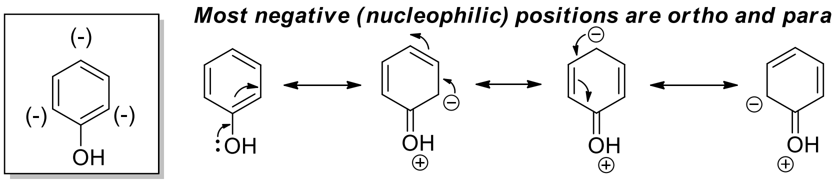

Question 3. If the same reaction conditions are applied to aniline, rather than anisole, the reaction is very slow and gives the meta product. Explain why, using structures and resonance. (HINT – think about the conditions used, and the nature of the two substituents).

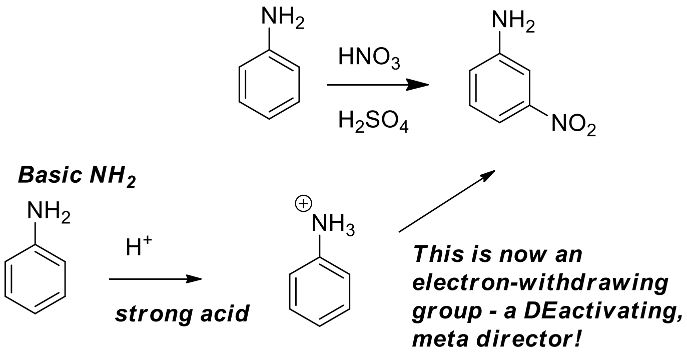

Core Ideas: molecular structure & properties; change & stability in chemical systems; energy

Scientific Practices: developing & using models; constructing explanations; engaging in argument from evidence

Crosscutting Concepts: cause & effect (mechanism & explanation; energy & matter; structure & function; stability & change

As the reaction is strongly acidic, the NH_2 gets protonated - the NH_3^+ is an electron-withdrawing group. The nitration is slower (worse nucleophile), and occurs meta.

K: 008B Learning Module 4: Alcohols

Question 1. Draw the arrow-pushing mechanism for the transformation shown below.

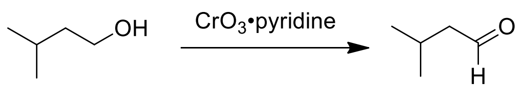

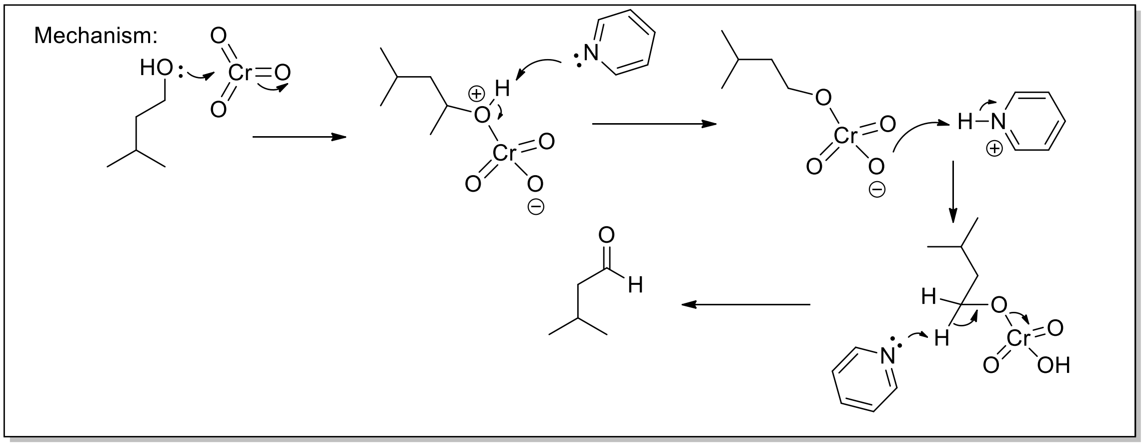

Question 2. If you try and do two oxidations on butane-1,4-diol, you don't make the dialdehyde, but make γ-butyrolactone instead. Explain why this happens, and draw the arrow-pushing mechanism.

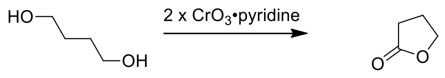

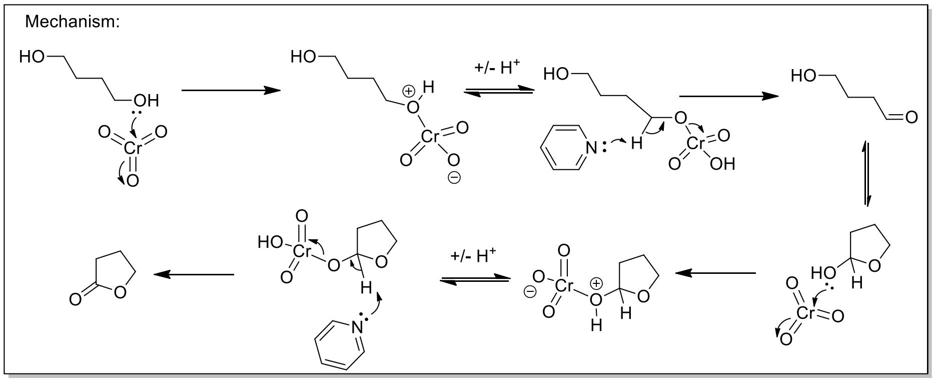

Core Ideas: molecular structure & properties; change & stability in chemical systems; energy

Scientific Practices: developing & using models; constructing explanations; engaging in argument from evidence

Crosscutting Concepts: cause & effect (mechanism & explanation; energy & matter; structure & function;

Explanation: ***The alcohol in this case is a good nucleophile, so adds to the C=O before the second oxidation. The 5-membered ring formed is stable, and aids the intramolecular reaction.***

L: 008B Learning Module 5: Aldehydes and Ketones

Question 1. Draw the arrow-pushing mechanism for the transformation shown below, which gives a mixture of two enamine products. Explain why the major isomer of product is formed preferentially.

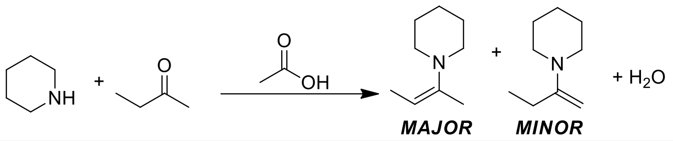

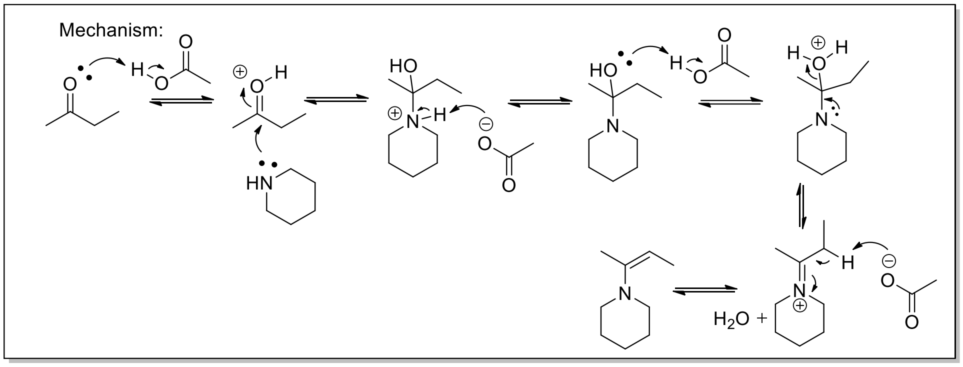

The most substituted alkene is the most favorable, which is the "major" product. The difference in energy is small, though, so you get a mixture.

Question 2. Why is acetic acid used as reagent? What would happen if you used HCl instead?

HCl is a very strong acid, and would make the ammonium salt below, which cannot participate in the reaction. Acetic acid is a weaker acid, so the protonation is reversible.

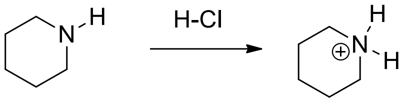

not a nucleophile

Question 3. Draw the product you would get from the reaction below. This reaction has multiple isomer possibilities, bit ***only one*** is formed: explain why you chose the isomer of product that you did.

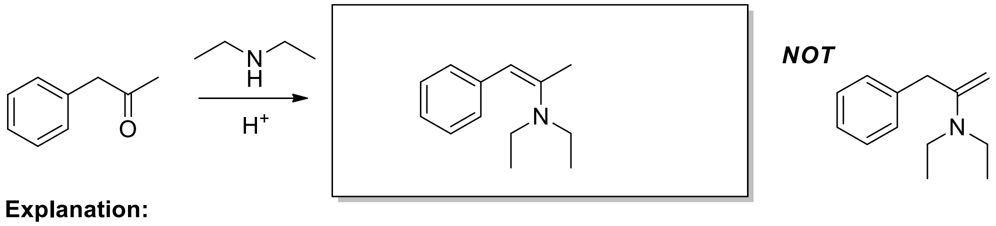

Explanation:

The molecule above has its double bond in conjugation with the phenyl group. The other isomer doesn't, so will be less stable - as this is an equilibrium process, the more thermodynamically favored product will be formed.

Core Ideas: molecular structure & properties; change & stability in chemical systems; energy

Scientific Practices: developing & using models; constructing explanations; engaging in argument from evidence

Crosscutting Concepts: cause & effect (mechanism & explanation; energy & matter; structure & function; stability & change
