# Supplementary material for: Using Flipped Classroom Modules to Facilitate Higher Order Learning in Undergraduate Organic Chemistry
Source: J Chem Educ. 2024 Jan 25;101(2):490–500. doi: 10.1021/acs.jchemed.3c00907 (PMC10867829; doi:10.1021/acs.jchemed.3c00907)
Supplement: Supplementary file 2 — ed3c00907_si_002.pdf [file ed3c00907_si_002.pdf]

Supporting Information – Appendix 2

Using Flipped Classroom Modules to Facilitate Higher Order Learning in Undergraduate Organic Chemistry

Lauren R. Holloway[a], Tabitha F. Miller[a], Bryce da Camara[a], Paul M. Bogie[a], Briana L. Hickey[a], Angie L. Lopez[a], Jiho Ahn, Eric Dao[a], Nicole Naibert[b], Jack Barbera[c], Richard J. Hooley[a]*, Jack F. Eichler[a]*

[a]Department of Chemistry, University of California-Riverside; Riverside, CA, 92521, USA.
[b]Portland Community College; Portland, OR, 97201
[c]Portland State University; Portland, OR, 97280

*E-mail: richard.hooley@ucr.edu; jack.eichler@ucr.edu

Supporting Information – Appendix 2

Table of Contents

A. General Information ……………………………………………………………………..*S-2*

B. Statistical Analyses – CHEM 008A Fall 2017……………………………………………..*S-4*

C. Statistical Analyses – CHEM 008A Fall 2018……………………………………………..*S-7*

D. Statistical Analyses – CHEM 008A Fall 2019……………………………………………...*S-10*

E. Statistical Analyses – CHEM 008A Fall 2022……………………………………………….*S-13*

F. Statistical Analyses – CHEM 008B Winter 2018..……………………………………….*S-18*

G. Statistical Analyses – CHEM 008B Winter 2019..……………………………………….*S-21*

H. Statistical Analyses – CHEM 008B Winter 2023..……………………………………….*S-24*

I. Statistical Analyses – CHEM 008B Winter 2023 AcES Survey……………………….*S-29*

A. General Information and Additional Notes

This appendix provides summaries of all statistical tests for the seven courses included in this study. The descriptive statistics and statistical analyses included in the study are summarized below.

1. Pearson's correlations for the set of dependent and independent variables are provided for each course analyzed in the study.

2. The raw SPSS output tables and graphs for the ANCOVAs are provided for each course analyzed in the study (except for CHEM 008A Fall 2022 and CHEM 008B Winter 2023). The tests of assumptions for the ANCOVA's are also provided for each class analyzed in the study and include:

> a) the normality of the final exam dependent variable across both levels of module score performance was evaluated using skewness and kurtosis data (normality is assumed if the absolute value of skewness is < 1.0 and the absolute value is less than three times the standard error; and the absolute value of kurtosis is < 2.0 and the absolute value is less than three times the standard error);
>
> b) the equality of variance of the dependent variable between groups was evaluated using Levene's test (a p value > 0.05 indicates the null hypothesis stating the variance of the dependent variable is equal across study groups cannot be rejected);
>
> c) the linearity of the dependent variable and covariate, and the homogeneity of regression slopes were evaluated by visual inspection (if the homogeneity of regression slopes was not clearly evident based on visual inspection, a model including the "study group*GPA" interaction was included; if this variable was statistically significant in the ANCOVA model, the homogeneity of regression slopes assumption was not met).

3. All of the data sets met the assumptions for ANCOVA except for CHEM 008A Fall 2022 (the assumption for multi-collinearity of the independent variables was not met) and CHEM 008B Winter 2023 (the assumption for homogeneity of dependent variable across study groups was not met). Therefore a multiple linear regression analysis that included a "module score*GPA" interaction term was carried out for these two courses. The raw data output for these analyses, the regression plots of Final Exam vs. GPA across three levels of module score performance, and the tests for assumptions are provided for these two courses.

Section A: CHEM 008A Fall 2017
Section B: CHEM 008A Fall 2018
Section C: CHEM 008A Fall 2019
Section D: CHEM 008A Fall 2022
Section E: CHEM 008B Winter 2018
Section F: CHEM 008B Winter 2019
Section G: CHEM 008B Winter 2023 *S-3*

4. Additional notes on the data analysis:

a) It was noted in Table 1 of the manuscript that 38-46% of students in the CHEM 008B courses had taken CHEM 008A in the flipped format, and completion of the 008A flipped course might have impacted student performance in the 008B course. However, since comparisons of student final exam performance between the different years/cohorts were not carried out, a separate analysis of the impact of in-class module activities on final exam performance for students who took both courses in the flipped format was not included.

b) The multiple regression models described below looked at the impact of module score on final exam score, while including university GPA and the GPA-Module Score interaction term as additional independent variables. Because GPA appeared to moderate module score performance, regression plots of final exam score versus university GPA were created across three levels of module score (low, moderate, and high module score).

c) At the beginning of each AcES survey, students were first asked if they had worked with others on the module or only worked by themselves, then asked to select the topic of the module. This was used as a check item to remove responses from students who may not have participated in the activity. A second check was included within the AcES items and asked students to select a specific response (i.e., *somewhat agree*). This item was used to remove responses from students who may not have been reading the survey items clearly while responding. All students were asked to respond to behavioral/cognitive (BC) and emotional (E) engagement AcES items, which were administered on a Likert-type scale from *strongly disagree* (1) to *strongly agree* (6). All negatively worded items were reverse coded before structural analysis. Module scores and AcES survey responses from consenting students for all modules were combined into an aggregated data set. Only respondents who received a score on a module activity were included in the data set. Thus, each student may be represented up to once for each module, for a maximum of five times in the aggregated data set. Descriptive statistics for the aggregated data set are included in the Supporting Information (SI Appendix 2, section 1H).

B. Statistical Analyses – CHEM 008A Fall 2017

1. Pearson's correlation (* denotes significance at 0.05 level; ** denotes significance at 0.01 level).

	Module Score	Final Exam Score	GPA
**Module Score**	-	0.649**	0.315**
**Final Exam Score**	-	-	0.486**

2. Assumptions for ANCOVA

a. Normality (skewness and kurtosis for Final Exam Dependent Variable)

	Skewness (Std. Error)	Kurtosis (Std. Error)
**Above Median Module Score**	-0.101 (0.258)	-0.093 (0.511)
**Below Median Module Score**	0.067 (0.235)	-0.773 (0.465)

b. Test of Equality of Variances Between Groups

Levene's Test of Equality of Error Variances[a]

Dependent Variable: finan_exam

F	df1	df2	Sig.
3.141	1	191	.078

Tests the null hypothesis that the error variance of the dependent variable is equal across groups.

a. Design: Intercept + gpa + median_split_module_groups

c. Linearity of Dependent Variable/Covariate

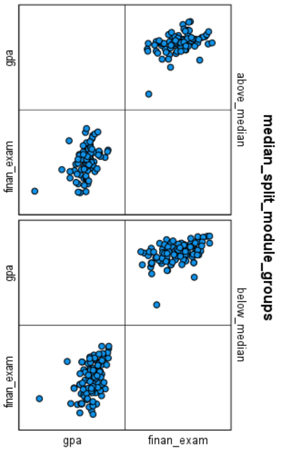

d. Test of Homogeneity of Regression Slopes

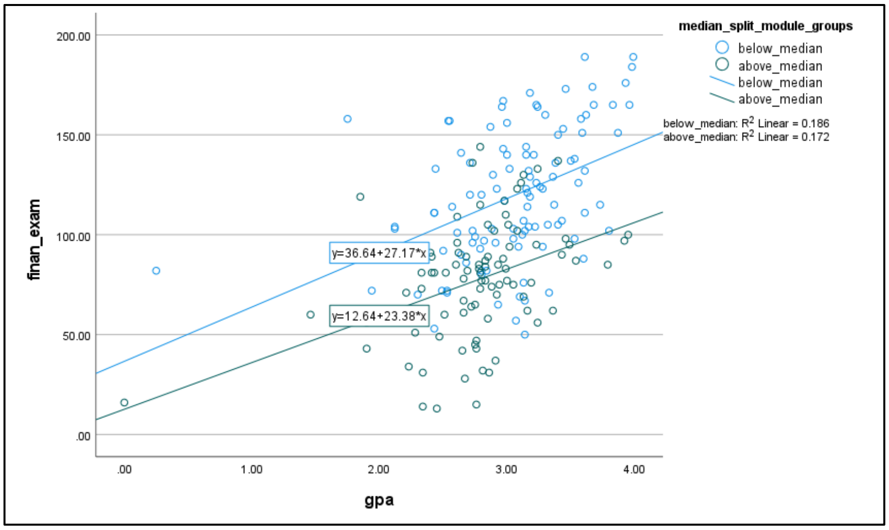

3. Raw ANCOVA Output Tables

Tests of Between-Subjects Effects

Dependent Variable: finan_exam

Source	Type III Sum of Squares	df	Mean Square	F	Sig.	Partial Eta Squared	Noncent. Parameter	Observed Power[b]
Corrected Model	119869.176[a]	2	59934.588	72.620	<.001	.433	145.240	1.000
Intercept	3557.370	1	3557.370	4.310	.039	.022	4.310	.542
gpa	34326.427	1	34326.427	41.592	<.001	.180	41.592	1.000
median_split_module_groups	54398.153	1	54398.153	65.912	<.001	.258	65.912	1.000
Error	156810.897	190	825.321					
Total	2230151.000	193						
Corrected Total	276680.073	192						

a. R Squared = .433 (Adjusted R Squared = .427)

b. Computed using alpha = .05

Estimates

Dependent Variable: finan_exam

median_split_module_groups	Mean	Std. Error	95% Confidence Interval Lower Bound	95% Confidence Interval Upper Bound
below_median	116.375[a]	2.837	110.778	121.971
above_median	81.394[a]	3.143	75.195	87.593

a. Covariates appearing in the model are evaluated at the following values: gpa = 2.9269.

4. Plot of final exam score vs. GPA across three levels (low, moderate, high) of total module scores; low = 0-13 module score (*n* = 60); medium = 14-18 module score (*n* = 68); high = 19-25 module score (*n* = 65):

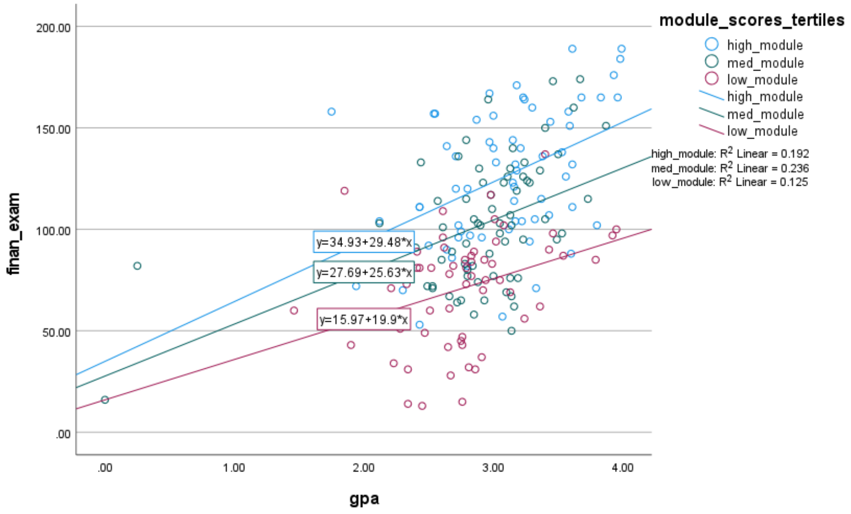

C. Statistical Analyses – CHEM 008A Fall 2018

1: Pearson's correlation (* denotes significance at 0.05 level; ** denotes significance at 0.01 level).

	Module Score	Final Exam Score	GPA
**Module Score**	-	0.531**	0.239**
**Final Exam Score**	-	-	0.256**

2. Assumptions for ANCOVA

a. Normality (skewness and kurtosis for Final Exam Dependent Variable)

	Skewness (Std. Error)	Kurtosis (Std. Error)
**Above Median Module Score**	0.095 (0.211)	-1.092 (0.419)
**Below Median Module Score**	0.233 (0.211)	-0.506 (0.419)

b. Test of Equality of Variances Between Groups

Levene's Test of Equality of Error Variances[a]

Dependent Variable: final_exam

F	df1	df2	Sig.
3.240	1	262	.073

Tests the null hypothesis that the error variance of the dependent variable is equal across groups.

a. Design: Intercept + gpa + median_split_module_groups

c. Linearity of Dependent Variable/Covariate

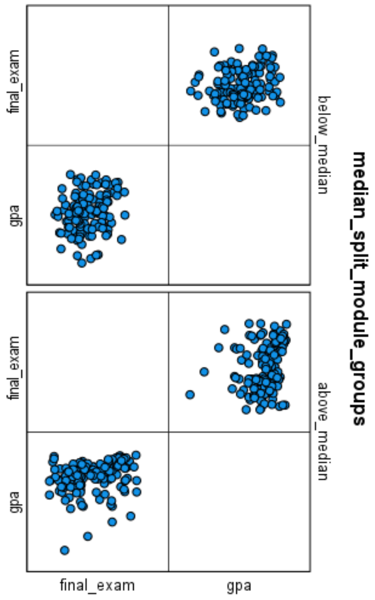

d. Test of Homogeneity of Regression Slopes

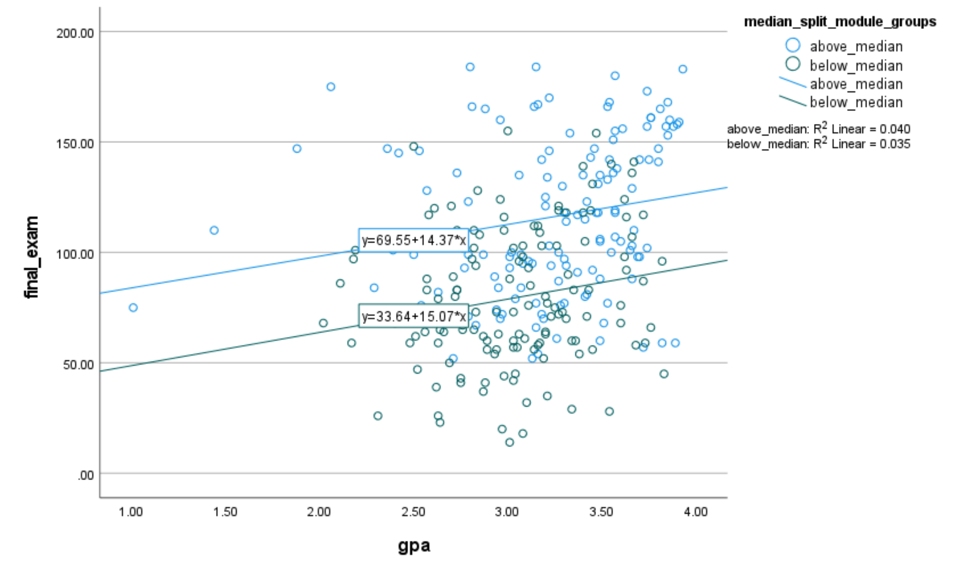

3. Raw ANCOVA Output Tables

Tests of Between-Subjects Effects

Dependent Variable: final_exam

Source	Type III Sum of Squares	df	Mean Square	F	Sig.	Partial Eta Squared	Noncent. Parameter	Observed Power[b]
Corrected Model	99736.775[a]	2	49868.388	46.239	<.001	.262	92.477	1.000
Intercept	13731.593	1	13731.593	12.732	<.001	.047	12.732	.945
gpa	11076.760	1	11076.760	10.270	.002	.038	10.270	.891
median_split_module_groups	71321.679	1	71321.679	66.130	<.001	.202	66.130	1.000
Error	281489.346	261	1078.503					
Total	2910414.000	264						
Corrected Total	381226.121	263						

a. R Squared = .262 (Adjusted R Squared = .256)

b. Computed using alpha = .05

Estimates

Dependent Variable: final_exam

median_split_module_groups	Mean	Std. Error	95% Confidence Interval Lower Bound	95% Confidence Interval Upper Bound
above_median	114.734[a]	2.895	109.033	120.434
below_median	81.024[a]	2.895	75.323	86.724

a. Covariates appearing in the model are evaluated at the following values: gpa = 3.1467.

4. Plot of final exam score vs. GPA across three levels (low, moderate, high) of total module scores; low = 0-15 module score (*n* = 84); medium = 16-19 module score (*n* = 86); high = 20-25 module score (*n* = 94):

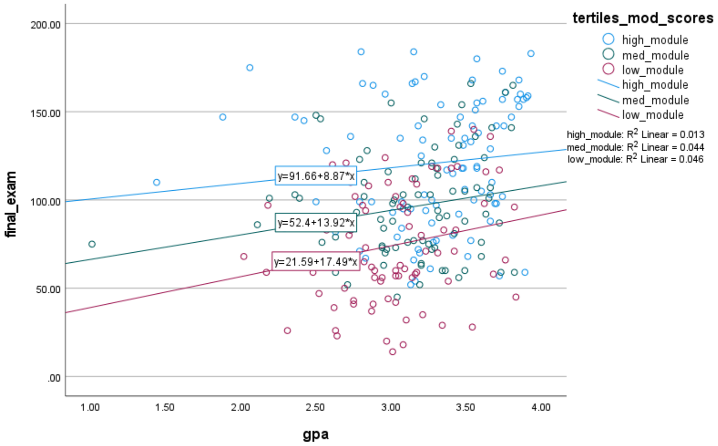

D. Statistical Analyses – CHEM 008A Fall 2019

1. Pearson's correlation (* denotes significance at 0.05 level; ** denotes significance at 0.01 level).

	Module Score	Final Exam Score	GPA
**Module Score**	-	0.520**	0.140*
**Final Exam Score**	-	-	0.020

2. Assumptions for ANCOVA

a. Normality (skewness and kurtosis for Final Exam Dependent Variable)

	Skewness (Std. Error)	Kurtosis (Std. Error)
**Above Median Module Score**	0.075 (0.211)	-0.525 (0.419)
**Below Median Module Score**	0.042 (0.211)	-0.314 (0.419)

b. Test of Equality of Variances Between Groups

Levene's Test of Equality of Error Variances[a]

Dependent Variable: final_exam

F	df1	df2	Sig.
.299	1	262	.585

Tests the null hypothesis that the error variance of the dependent variable is equal across groups.

a. Design: Intercept + gpa + median_split_groups

c. Linearity of Dependent Variable/Covariate

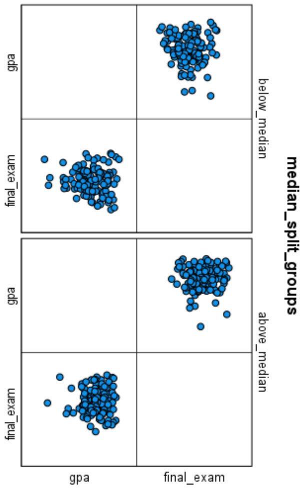

d. Test of Homogeneity of Regression Slopes

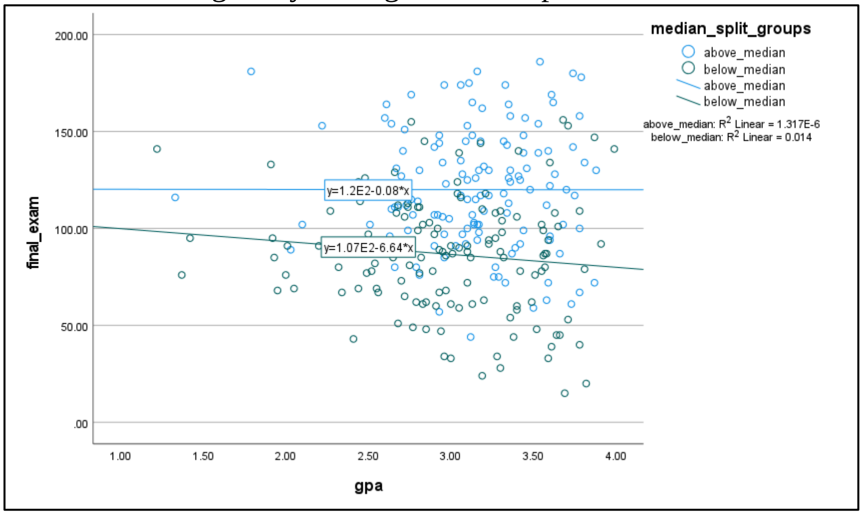

Tests of Between-Subjects Effects

Dependent Variable: final_exam

Source	Type III Sum of Squares	df	Mean Square	F	Sig.	Partial Eta Squared	Noncent. Parameter	Observed Power[b]
Corrected Model	74845.321[a]	3	24948.440	26.019	<.001	.231	78.057	1.000
Intercept	78697.760	1	78697.760	82.075	<.001	.240	82.075	1.000
median_split_groups	289.385	1	289.385	.302	.583	.001	.302	.085
gpa	672.156	1	672.156	.701	.403	.003	.701	.133
median_split_groups * gpa	639.300	1	639.300	.667	.415	.003	.667	.129
Error	249300.800	260	958.849					
Total	3145560.000	264						
Corrected Total	324146.121	263						

a. R Squared = .231 (Adjusted R Squared = .222)

b. Computed using alpha = .05

*Since visual inspection of the regression slopes indicates the impact of GPA on Final Exam score may not be equivalent between the groups, a module including the study group*GPA interaction was included; since this variable is not statistically significant in the ANCOVA model, the homogeneity of regression slopes can be assumed.

3.Raw ANCOVA Output Tables

Tests of Between-Subjects Effects

Dependent Variable: final_exam

Source	Type III Sum of Squares	df	Mean Square	F	Sig.	Partial Eta Squared	Noncent. Parameter	Observed Power[b]
Corrected Model	74206.021[a]	2	37103.011	38.745	<.001	.229	77.490	1.000
Intercept	88117.887	1	88117.887	92.017	<.001	.261	92.017	1.000
gpa	1072.551	1	1072.551	1.120	.291	.004	1.120	.184
median_split_groups	74070.421	1	74070.421	77.348	<.001	.229	77.348	1.000
Error	249940.100	261	957.625					
Total	3145560.000	264						
Corrected Total	324146.121	263						

a. R Squared = .229 (Adjusted R Squared = .223)

b. Computed using alpha = .05

Estimates

Dependent Variable: final_exam

median_split_groups	Mean	Std. Error	95% Confidence Interval	
			Lower Bound	Upper Bound
above_median	120.355[a]	2.712	115.015	125.694
below_median	86.403[a]	2.712	81.063	91.742

a. Covariates appearing in the model are evaluated at the following values: gpa = 3.0612.

4. Plot of final exam score vs. GPA across three levels (low, moderate, high) of total module scores; low = 2-13 module score (n = 75); medium = 14-18 module score (n = 99); high = 19-25 module score (n = 90):

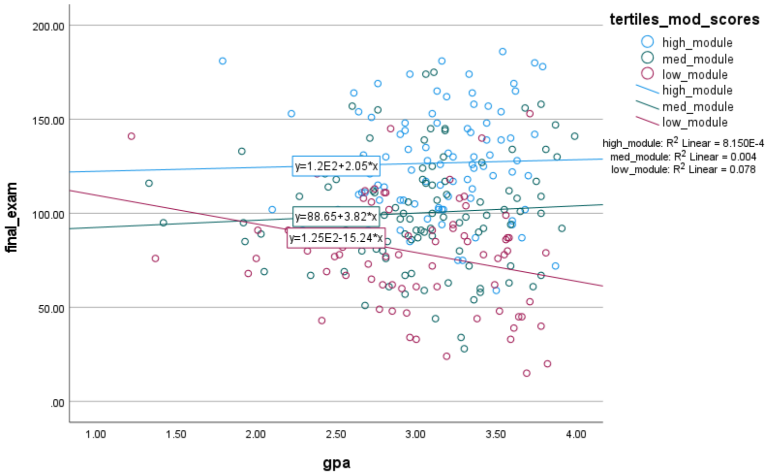

E. Statistical Analyses – CHEM 008A Fall 2022

1. Pearson's correlation (* denotes significance at 0.05 level; ** denotes significance at 0.01 level).

	**Module Score**	**Final Exam Score**	**GPA**
**Module Score**	-	0.643**	0.591**
**Final Exam Score**	-	-	0.717**

2. Assumptions for ANCOVA

a. Normality (skewness and kurtosis for Final Exam Dependent Variable)

	**Skewness (Std. Error)**	**Kurtosis (Std. Error)**
**Above Median Module Score**	-0.308 (0.219)	-0.950 (0.435)
**Below Median Module Score**	0.638 (0.212)	0.220 (0.422)

b. Test of Equality of Variances Between Groups

Levene's Test of Equality of Error Variances[a]

Dependent Variable: final_score

F	df1	df2	Sig.
3.376	1	250	.067

Tests the null hypothesis that the error variance of the dependent variable is equal across groups.

a. Design: Intercept + gpa + module_groups_median_split

c. Linearity of Dependent Variable/Covariate

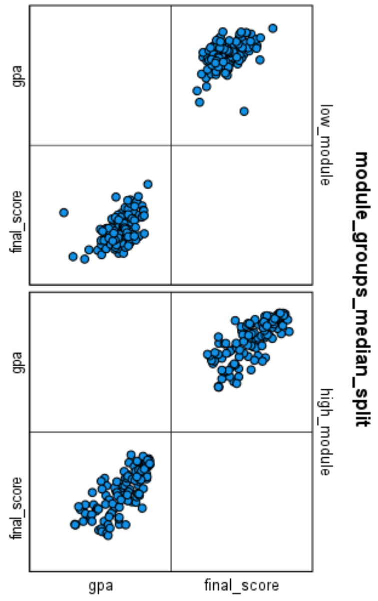

d. Test of Homogeneity of Regression Slopes

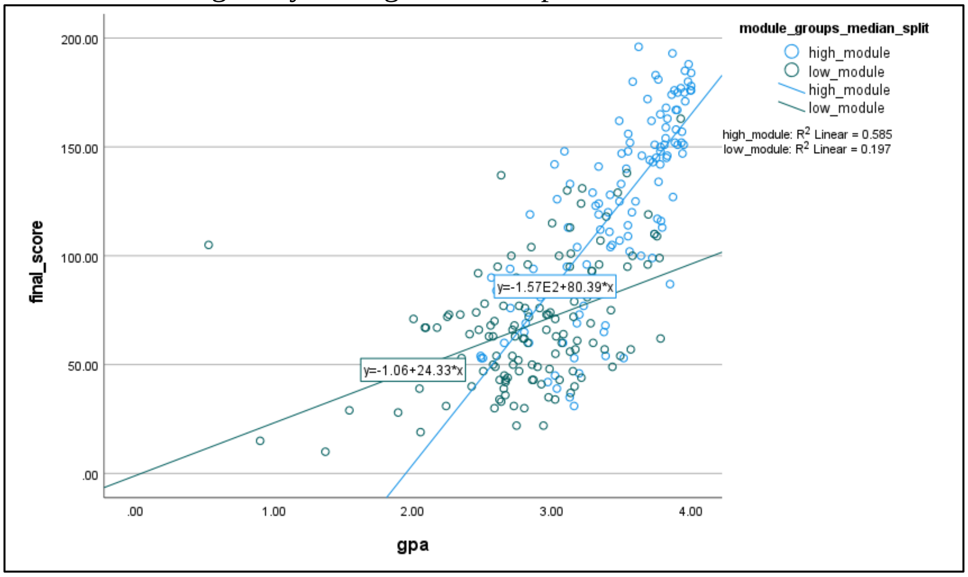

Tests of Between-Subjects Effects

Dependent Variable: final_score

Source	Type III Sum of Squares	df	Mean Square	F	Sig.
Corrected Model	335257.694[a]	3	111752.565	154.341	<.001
Intercept	29665.318	1	29665.318	40.971	<.001
module_groups_median_split	28869.629	1	28869.629	39.872	<.001
gpa	141580.360	1	141580.360	195.536	<.001
module_groups_median_split * gpa	40578.538	1	40578.538	56.043	<.001
Error	179567.874	248	724.064		
Total	2748459.000	252			
Corrected Total	514825.567	251			

a. R Squared = .651 (Adjusted R Squared = .647)

*Since visual inspection of the regression slopes indicates the impact of GPA on Final Exam score may not be equivalent between the groups, a module including the study group*GPA interaction was included; since this variable is statistically significant in the ANCOVA model, the homogeneity of regression slopes assumption is not met for this data set.

3. Multiple Linear Regression

Because the assumption of homogeneity of regression slopes was not met for the ANCOVA, , a multiple regression model was created to evaluate the impact of module score and GPA on final exam score.

a. model with only module score and GPA independent variables:

Model Summary

Model	R	R Square	Adjusted R Square	Std. Error of the Estimate
1	.767[a]	.588	.584	29.19513

a. Predictors: (Constant), gpa, module_score

Coefficients[a]

Model		Unstandardized Coefficients		Standardized Coefficients		
		B	Std. Error	Beta	t	Sig.
1	(Constant)	-68.760	10.622		-6.473	<.001
	module_score	2.243	.335	.337	6.689	<.001
	gpa	41.186	4.015	.517	10.258	<.001

a. Dependent Variable: final_score

b. model that includes Module Score*GPA interaction term with module score and GPA independent variables:

Model Summary

Model	R	R Square	Adjusted R Square	Std. Error of the Estimate
1	.799[a]	.639	.634	27.39342

a. Predictors: (Constant), gpa_module_interaction, module_score, gpa

Coefficients[a]

Model		Unstandardized Coefficients		Standardized Coefficients		
		B	Std. Error	Beta	t	Sig.
1	(Constant)	-79.272	10.125		-7.829	<.001
	module_score	2.072	.316	.312	6.558	<.001
	gpa	43.252	3.784	.543	11.432	<.001
	gpa_module_interaction	2.884	.489	.227	5.902	<.001

a. Dependent Variable: final_score

Model Summary

Model	R	R Square	Adjusted R Square	Std. Error of the Estimate	Change Statistics				
					R Square Change	F Change	df1	df2	Sig. F Change
1	.767[a]	.588	.584	29.19513	.588	177.502	2	249	<.001
2	.799[b]	.639	.634	27.39342	.051	34.831	1	248	<.001

a. Predictors: (Constant), gpa, module_score

b. Predictors: (Constant), gpa, module_score, gpa_module_interaction

c. Plot of final exam score vs. GPA across three levels (low, moderate, high) of total module scores; low = 0-11 module score (n = 88); medium = 12-18 module score (n = 67); high = 19-25 module score (n = 97):

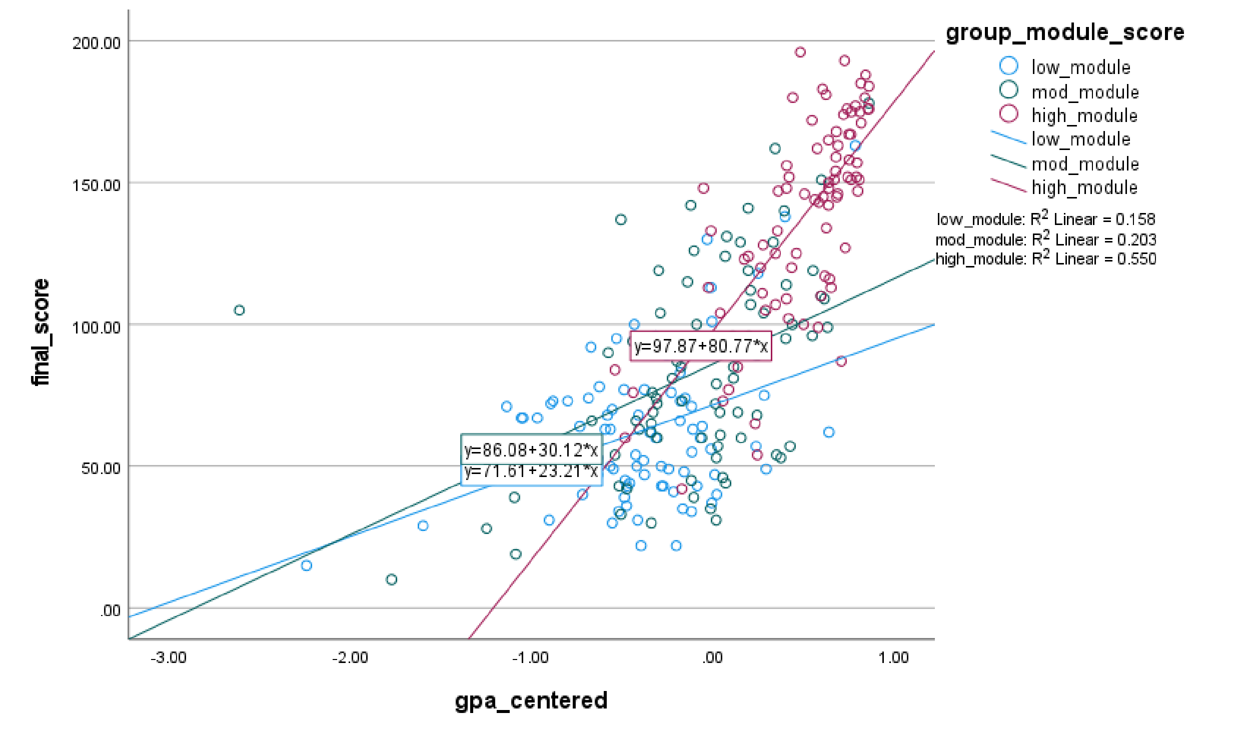

4. Multiple Linear Regression Assumptions:

a. linearity between dependent variable and independent variables (visual inspection indicates an approximate linear relationship between the DV and IVs):

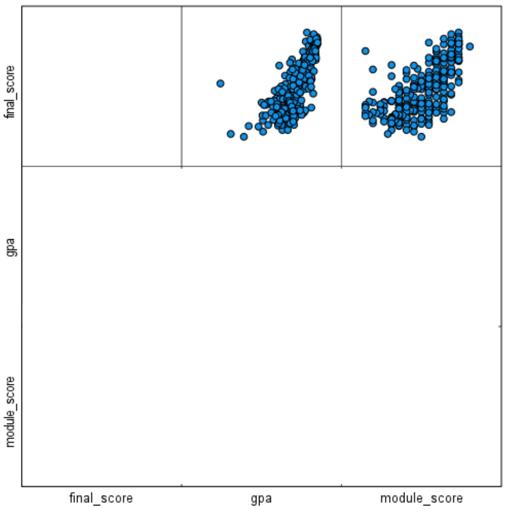

b. normality of residuals (visual inspection indicates the residuals are approximately normally distributed: evidenced by an approximately linear fit of the normal P-P plot of regression standardized residuals):

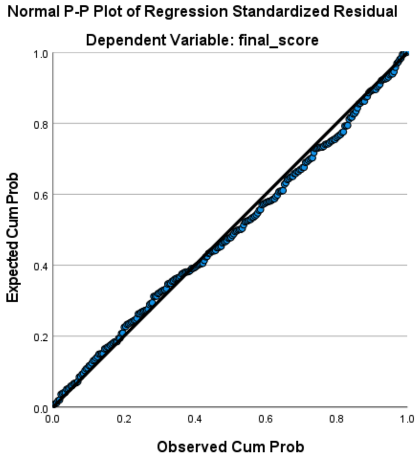

c. constant variance of the residuals (visual inspection indicates the standardized residuals are homoscedastic; evidenced by an approximately random distribution of observed vs. predicted standardized residuals between -3 and +3):

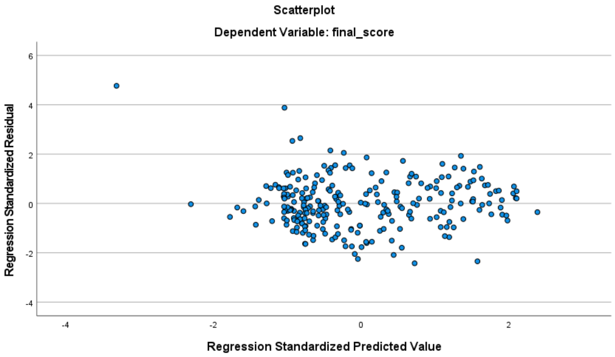

F. Statistical Analyses – CHEM 008B Winter 2018

1. Pearson's correlations (* denotes significance at 0.05 level; ** denotes significance at 0.01 level).

	Module Score	Final Exam Score	GPA
**Module Score**	-	0.534**	0.208**
**Final Exam Score**	-	-	0.223**

2. Assumptions for ANCOVA

a. Skewness and Kurtosis (Final Exam Score Dependent Variable)

	Skewness (Std. Error)	Kurtosis (Std. Error)
**Above Median Module Score**	-0.54 (0.238)	-0.975 (0.472)
**Below Median Module Score**	0.598 (0.238)	-0.291 (0.472)

b. Test for Equality of Variances Between Groups

Levene's Test of Equality of Error Variances[a]

Dependent Variable: final_exam

F	df1	df2	Sig.
3.320	1	204	.070

Tests the null hypothesis that the error variance of the dependent variable is equal across groups.

a. Design: Intercept + gpa + Median_split_module_groups

c. Linearity of Dependent Variable/Covariate

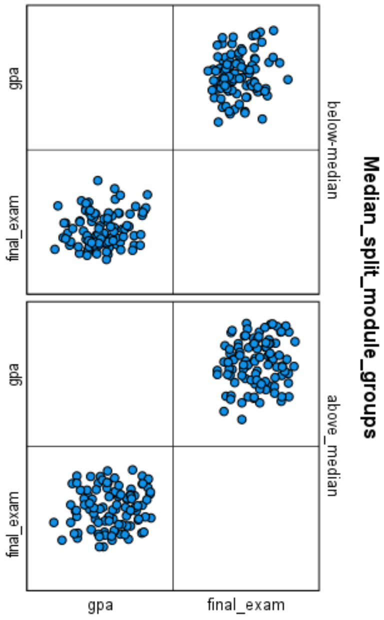

d. Test of Homogeneity of Regression Slopes

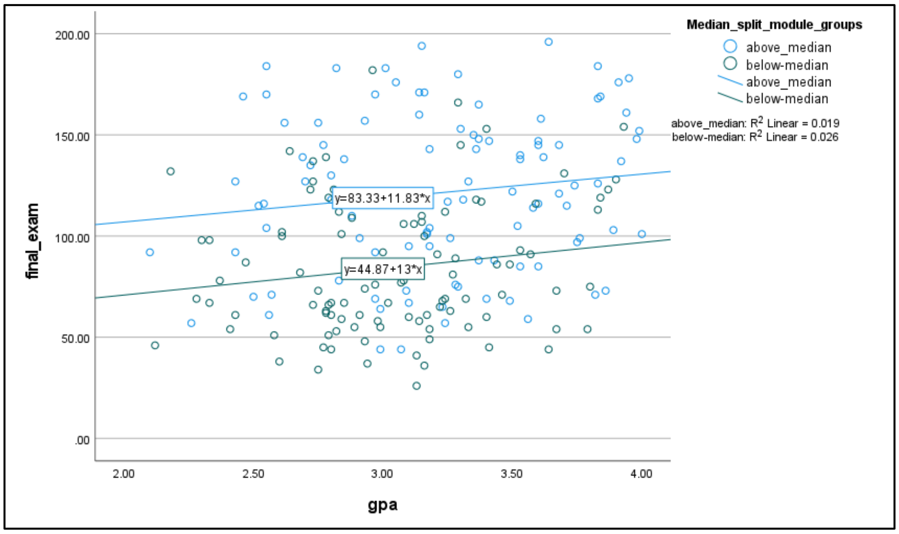

3.Raw Output ANCOVA Tables

Tests of Between-Subjects Effects

Dependent Variable: final_exam

Source	Type III Sum of Squares	df	Mean Square	F	Sig.	Partial Eta Squared	Noncent. Parameter	Observed Power[b]
Corrected Model	76452.532[a]	2	38226.266	29.900	<.001	.228	59.800	1.000
Intercept	15704.950	1	15704.950	12.284	<.001	.057	12.284	.937
gpa	5800.955	1	5800.955	4.537	.034	.022	4.537	.564
Median_split_module_groups	59751.445	1	59751.445	46.737	<.001	.187	46.737	1.000
Error	259529.647	203	1278.471					
Total	2518759.000	206						
Corrected Total	335982.180	205						

a. R Squared = .228 (Adjusted R Squared = .220)

b. Computed using alpha = .05

Estimates

Dependent Variable: final_exam

Median_split_module_groups	Mean	Std. Error	95% Confidence Interval Lower Bound	95% Confidence Interval Upper Bound
above_median	120.340[a]	3.562	113.317	127.363
below-median	85.533[a]	3.562	78.510	92.556

a. Covariates appearing in the model are evaluated at the following values: gpa = 3.1336.

4. Plot of final exam score vs. GPA across three levels (low, moderate, high) of total module scores; low = 3-16 module score (*n* = 60); medium = 17-20 module score (*n* = 75); high = 21-25 module score (*n* = 71):

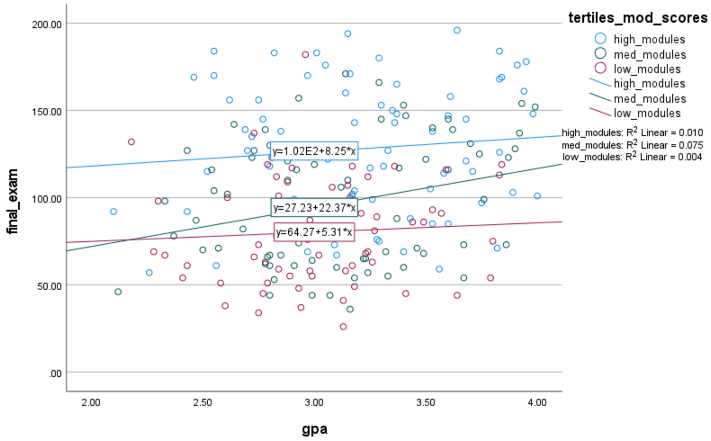

G. Statistical Analyses – CHEM 008B Winter 2019

1. Pearson's correlation (* denotes significance at 0.05 level; ** denotes significance at 0.01 level).

	Module Score	Final Exam Score	GPA
**Module Score**	-	0.606**	0.199**
**Final Exam Score**	-	-	0.152*

2. Assumptions for ANCOVA

a. Skewness and Kurtosis (Final Exam Score Dependent Variable)

	Skewness (Std. Error)	Kurtosis (Std. Error)
**Above Median Module Score**	-0.54 (0.238)	-0.975 (0.472)
**Below Median Module Score**	0.598 (0.238)	-0.291 (0.472)

b. Test for Equality of Variances Between Groups

Levene's Test of Equality of Error Variances[a]

Dependent Variable: final_exam

F	df1	df2	Sig.
.910	1	218	.341

Tests the null hypothesis that the error variance of the dependent variable is equal across groups.

a. Design: Intercept + gpa + median_split_module_groups

c. Linearity of Dependent Variable/Covariate

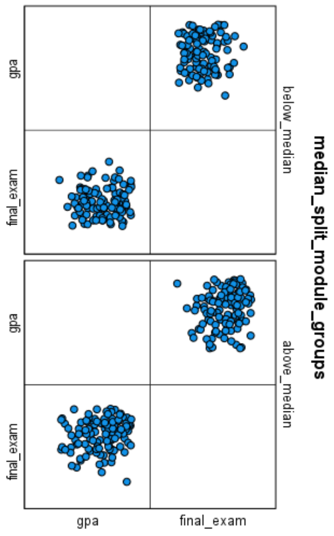

d. Test of Homogeneity of Regression Slopes

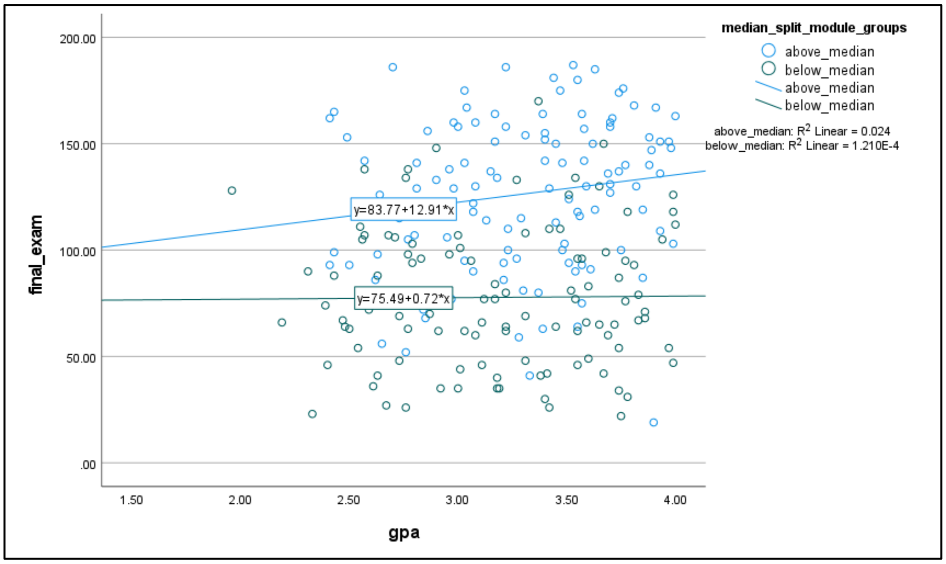

*Since visual inspection of the regression slopes indicates the impact of GPA on Final Exam score may not be equivalent between the groups, a module including the study group*GPA interaction was included; since this variable is not statistically significant in the ANCOVA model, the homogeneity of regression slopes can be assumed.

Tests of Between-Subjects Effects

Dependent Variable: final_exam

Source	Type III Sum of Squares	df	Mean Square	F	Sig.
Corrected Model	134224.542[a]	3	44741.514	38.174	<.001
Intercept	26930.670	1	26930.670	22.978	<.001
median_split_module_groups	72.848	1	72.848	.062	.803
gpa	2131.423	1	2131.423	1.819	.179
median_split_module_groups * gpa	1704.941	1	1704.941	1.455	.229
Error	253159.567	216	1172.035		
Total	2713954.000	220			
Corrected Total	387384.109	219			

a. R Squared = .346 (Adjusted R Squared = .337)

3.Raw Output ANCOVA Tables

Tests of Between-Subjects Effects

Dependent Variable: final_exam

Source	Type III Sum of Squares	df	Mean Square	F	Sig.	Partial Eta Squared	Noncent. Parameter	Observed Power[b]
Corrected Model	132519.601[a]	2	66259.800	56.416	<.001	.342	112.832	1.000
Intercept	29697.744	1	29697.744	25.286	<.001	.104	25.286	.999
gpa	1687.861	1	1687.861	1.437	.232	.007	1.437	.223
median_split_module_groups	123608.983	1	123608.983	105.245	<.001	.327	105.245	1.000
Error	254864.508	217	1174.491					
Total	2713954.000	220						
Corrected Total	387384.109	219						

a. R Squared = .342 (Adjusted R Squared = .336)

b. Computed using alpha = .05

Estimates

Dependent Variable: final_exam

median_split_module_groups	Mean	Std. Error	95% Confidence Interval	
			Lower Bound	Upper Bound
above_median	126.161[a]	3.242	119.772	132.550
below_median	78.204[a]	3.332	71.636	84.771

a. Covariates appearing in the model are evaluated at the following values: gpa = 3.2477.

4. Plot of final exam score vs. GPA across three levels (low, moderate, high) of total module scores; low = 1-13 module score (n = 68); medium = 14-17 module score (n = 90); high = 18-25 module score (n = 62):

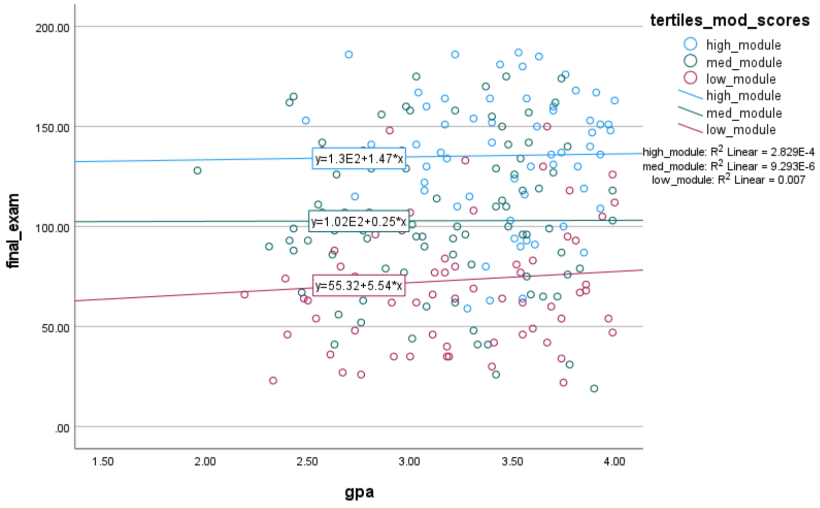

H. Statistical Analyses – CHEM 008B Winter 2023

1: Pearson's correlation (* denotes significance at 0.05 level; ** denotes significance at 0.01 level).

	Module Score	Final Exam Score	GPA
**Module Score**	-	0.563**	0.524**
**Final Exam Score**	-	-	0.690*

2. Assumptions for ANCOVA

a. Skewness and Kurtosis (Final Exam Score Dependent Variable)

	Skewness (Std. Error)	Kurtosis (Std. Error)
**Above Median Module Score**	0.162 (0.226)	-1.157 (0.447)
**Below Median Module Score**	0.662 (0.226)	0.337 (0.447)

b. Test for Equality of Variances Between Groups

Levene's Test of Equality of Error Variances[a]

Dependent Variable: Final_exam

F	df1	df2	Sig.
20.671	1	228	<.001

Tests the null hypothesis that the error variance of the dependent variable is equal across groups.

a. Design: Intercept + GPA + Median_split_module_scores

Since Levene's test yielded a statistically significant result, the assumption of equal variance for the dependent variable across the two study groups is not met [final exam mean for Above Median Module group = 107.5 +/- 41.5; final exam median for Below Median Module group = 65.3 +/- 29.2].

c. Linearity of Dependent Variable/Covariate

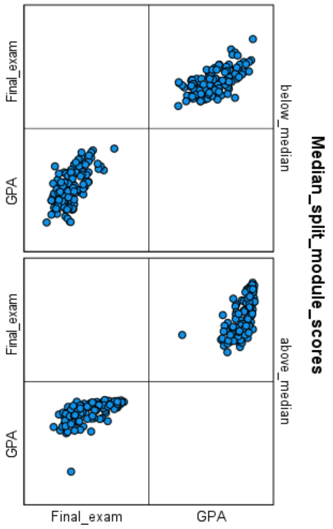

d. Test of Homogeneity of Regression Slopes

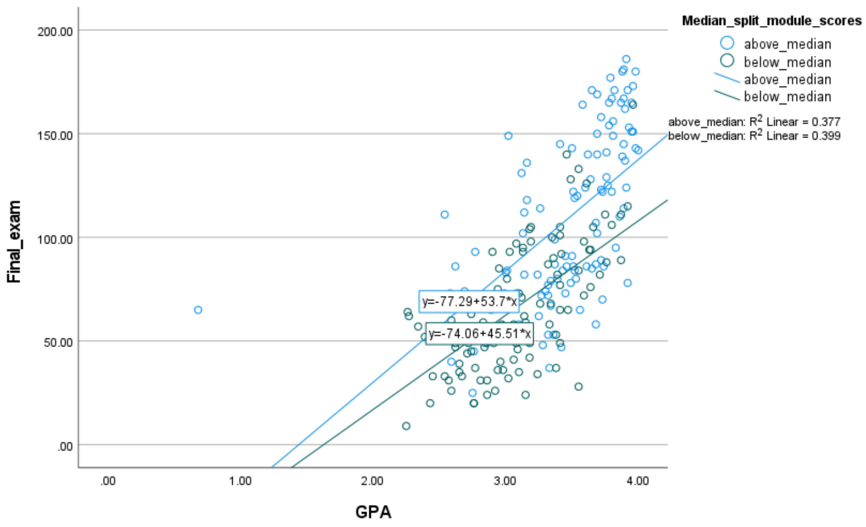

*Since visual inspection of the regression slopes indicates the impact of GPA on Final Exam score may not be equivalent between the groups, a module including the study group*GPA interaction was included; since this variable is not statistically significant in the ANCOVA model, the homogeneity of regression slopes can be assumed.

Tests of Between-Subjects Effects

Dependent Variable: Final_exam

Source	Type III Sum of Squares	df	Mean Square	F	Sig.
Corrected Model	214813.980[a]	3	71604.660	89.596	<.001
Intercept	23366.164	1	23366.164	29.237	<.001
Median_split_module_scores	10.659	1	10.659	.013	.908
GPA	106466.086	1	106466.086	133.216	<.001
Median_split_module_scores * GPA	726.466	1	726.466	.909	.341
Error	180618.581	226	799.197		
Total	2111855.000	230			
Corrected Total	395432.561	229			

a. R Squared = .543 (Adjusted R Squared = .537)

3. Multiple Linear Regression

Because the assumption of equal variance in the dependent variable across the two study groups was not met for the ANCOVA, a multiple regression model was created to evaluate the impact of module score and GPA on final exam score.

a. hierarchical model with only module score and GPA independent variables, and model including module score, GPA, and the GPA*Module Score interaction term:

Coefficients[a]

Model		Unstandardized Coefficients B	Unstandardized Coefficients Std. Error	Standardized Coefficients Beta	t	Sig.
1	(Constant)	-104.207	12.977		-8.030	<.001
	Module_scores	2.240	.431	.277	5.196	<.001
	GPA	47.232	4.627	.544	10.207	<.001
2	(Constant)	-105.593	12.927		-8.169	<.001
	Module_scores	2.393	.436	.296	5.483	<.001
	GPA	46.294	4.629	.534	10.001	<.001
	module_score_GPA	1.492	.795	.086	1.876	.062

a. Dependent Variable: Final_exam

Model Summary[c]

Model	R	R Square	Adjusted R Square	Std. Error of the Estimate	Change Statistics: R Square Change	F Change	df1	df2	Sig. F Change
1	.729[a]	.531	.527	28.57524	.531	128.638	2	227	<.001
2	.734[b]	.538	.532	28.41807	.007	3.518	1	226	.062

a. Predictors: (Constant), GPA, Module_scores

b. Predictors: (Constant), GPA, Module_scores, module_score_GPA

c. Dependent Variable: Final_exam

b. Plot of final exam score vs. GPA across three levels (low, moderate, high) of total module scores; low = 5-14 module score (n = 78); medium = 15-18 module score (n = 76); high = 19-25 module score (n = 76):

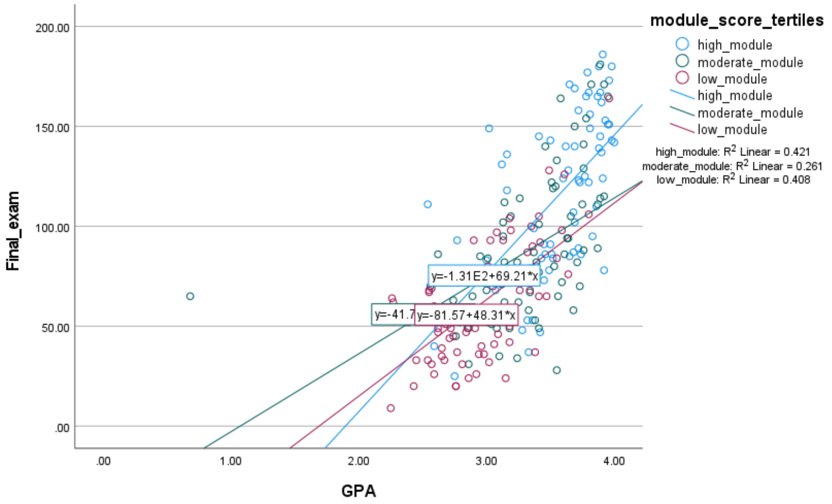

4. Multiple Linear Regression Assumptions:
a. linearity between dependent variable and independent variables (visual inspection indicates an approximate linear relationship between the DV and IVs):

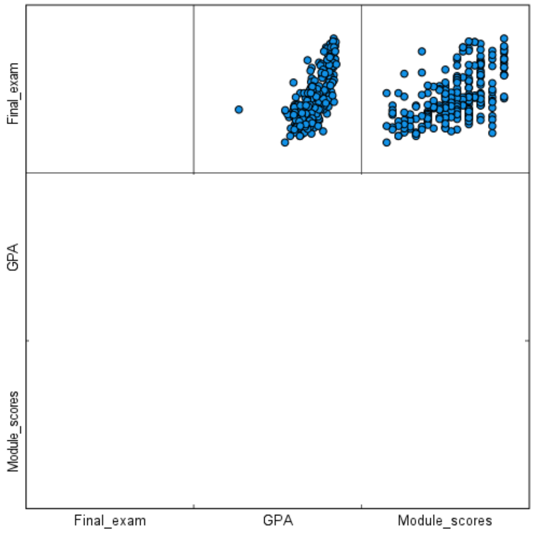

b. normality of residuals (visual inspection indicates the residuals are approximately normally distributed; evidenced by an approximately linear fit of the normal P-P plot of regression standardized residuals):

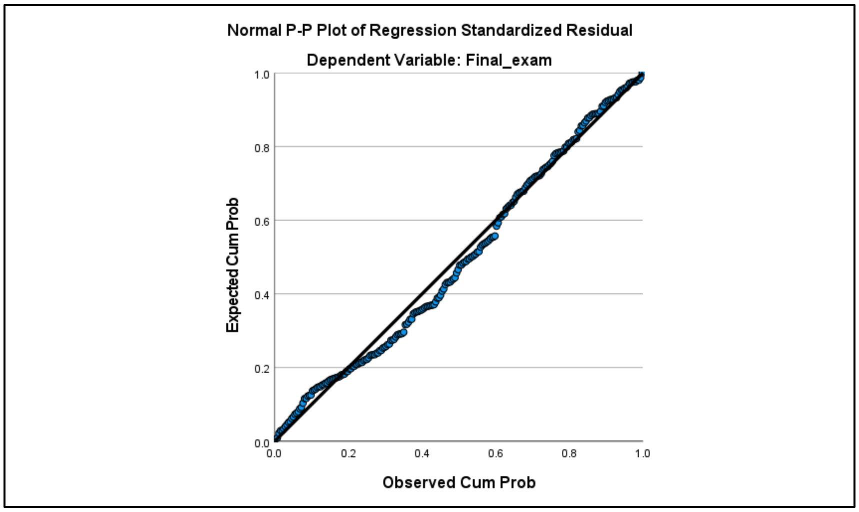

c. constant variance of the residuals (visual inspection indicates the standardized residuals are homoscedastic; evidenced by an approximately random distribution of observed vs. predicted standardized residuals between -3 and +3):

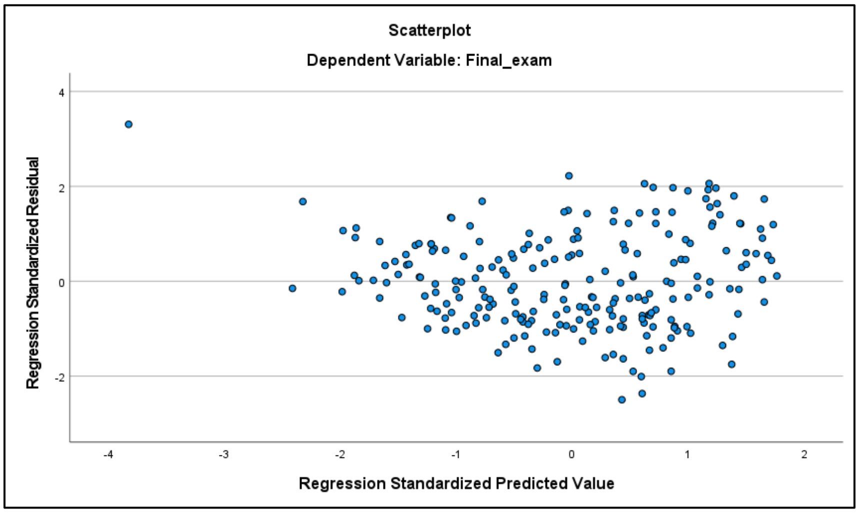

I. Data tables for AcES survey analysis:

1. AcES item means and standard deviations (SD) for the aggregated data set (n = 828).

Item Number	Item	Mean (SD)
BC1	I stayed focused during today's Learning Module.	5.02 (0.88)
BC2	I put effort into doing today's Learning Module.	5.15 (0.83)
BC3	I kept working on today's activity even if something was hard.	5.22 (0.77)
BC4	I didn't do much work on today's Learning Module.	2.03 (1.03)
BC5	I attempted to answer most of the items on today's Learning Module.	5.33 (0.80)
BC6	I made sure I understood my work on today's Learning Module.	4.79 (1.00)
BC7	I tried to connect what I was learning during today's Learning Module to concepts I have learned before.	4.98 (0.89)
BC8	I tried to understand my mistakes when I got something wrong during today's Learning Module.	4.95 (0.88)
BC9	I wrote down the answers to today's Learning Module without trying to understand them.	2.22 (1.15)
BC10	I didn't think very hard when I came across a challenging problem on today's Learning Module.	2.21 (1.08)
E1	I looked forward to today's Learning Module.	3.54 (1.32)
E2	I enjoyed learning the class material during today's Learning Module.	4.23 (1.14)
E3	I didn't want to do today's Learning Module.	2.70 (1.16)
E4	I didn't want to do today's Learning Module.	3.29 (1.38)
E5	I didn't care about doing today's Learning Module.	2.19 (1.10)

2. Data-model fit with aggregated data set (n = 828) for BC and E scales and a BC-E model (see Naibert & Barbera, 2022). Bolded values indicate results met the suggested criteria for good fit based on the recommendations from Hu and Bentler (1999); CFI ≥ 0.95, RMSEA ≤ 0.06, SRMR ≤ 0.08. Italicized values suggest results indicate reasonable fit; CFI ≥ 0.90, RMSEA ≤ 0.10 (Brown, 2005).

Scale/Model[a]	χ^2 (df)	p-value	CFI	RMSEA [90% CI]	SRMR	omega
BC	119.343 (32)	<0.001	**0.965**	*0.070* [0.057 – 0.083]	**0.032**	0.89
E	24.661 (2)	<0.001	**0.974**	0.147 [0.099 – 0.202]	**0.027**	0.82
BC-E Correlated	372.979 (83)	<0.001	*0.932*	*0.075* [0.068 – 0.083]	**0.068**	--

[a]Each scale/model includes a negative method factor to account for possible response bias for negatively-worded items (Ye, & Wallace, 2013).

3. Fit indices and change statistics for measurement invariance testing between low scoring (module score = 1, n = 111) and high scoring (module score = 5, n = 383) student respondents. Bolded values indicate the change meets the suggested recommendations from Chen (2007) for supporting invariance at each step.

Model	χ^2 (df)	p-value	CFI	RMSEA	SRMR	$\Delta\chi^2$ (Δdf)	ΔCFI	ΔRMSEA	ΔSRMR
Configural	351.343 (166)	<0.001	0.928	0.077	0.066	--	--	--	--
Metric	327.238 (184)	<0.001	0.925	0.075	0.074	-24.105 (18)	**-0.003**	**-0.002**	**0.008**
Scalar	414.470 (196)	<0.001	0.917	0.077	0.076	87.232 (12)	**-0.008**	**0.002**	**0.002**
Conservative	360.261 (211)	<0.001	0.923	0.071	0.080	-54.209 (15)	**0.006**	**-0.006**	**0.004**
