# Supplementary material for: Using Flipped Classroom Modules to Facilitate Higher Order Learning in Undergraduate Organic Chemistry
Source: J Chem Educ. 2024 Jan 25;101(2):490–500. doi: 10.1021/acs.jchemed.3c00907 (PMC10867829; doi:10.1021/acs.jchemed.3c00907)
Supplement: Supplementary file 3 — ed3c00907_si_003.pdf [file ed3c00907_si_003.pdf]

Chem 008A Final Exam, Dec 7th 2022

Name:____

Circle the FIRST LETTER of your LAST NAME:
A B C D E F G H I J K L M N O P Q R S T U V W X Y Z

STUDENT ID:__________________________

SEAT #:_______________________________

LAB DAY / TIME: ______________________

Lab TA's Name: ________________________

READ AND SIGN

"I understand that any observed or suspected improper behavior on my part will be reported to Student Judicial Affairs."

Signature:____________________

Unsigned exams will not be graded

Time: 3.00 h
Write your name on all pieces of paper used for this exam. Write your answers on the exam sheet. There are 10 questions in this exam; you have THREE hours to do them. You may use your four sheets of notes, but NO OTHER pieces of paper.

Q1 ________	(21)	Q6 ________	(20)
Q2 ________	(21)	Q7 ________	(18)
Q3 ________	(20)	Q8 ________	(20)
Q4 ________	(18)	Q9 ________	(20)
Q5 ________	(20)	Q10 _______	(22)

BONUS____ (5)

Total _______ (200)

Nucleophiles/Bases

Excellent Nucleophiles (S_N2)	Ambiguous (Good Base and Nucleophile – Both S_N2 and E2)	Good Bases, Poor Nucleophiles (E2)	Weak Bases/ Nucleophiles (S_N1)
-CN	-OH	$(CH_3)_3CO$-	H_2O
RS-	-OR	$-NH_2$	ROH
I-		H-	
N_3-		$-NR_2$	

Reaction Outcomes

	Poor Nu (e.g. H_2O, ROH	Good Nu, weak base (e.g. I-, RS-, -CN)	Good Nu, strong unhindered base (e.g. HO-, RO-)	Strong, hindered base $(CH_3)_3CO$-, R_2N- and H-	Silver cation
**methyl** 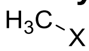	no rxn	S_N2	S_N2	S_N2	no rxn
**primary** 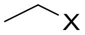	no rxn	S_N2	S_N2	E2	no rxn
**secondary** 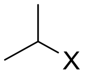	S_N1/E1	S_N2	E2	E2	S_N1/E1
**tertiary** 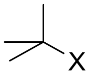	S_N1/E1	S_N1/E1	E2	E2	S_N1/E1
**allyl/benzyl** 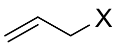 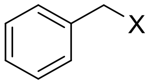	S_N1/E1	S_N2	S_N2	S_N2/E2	S_N1/E1

IR Stretching Frequencies (cm^{-1})

Alkenes
=C-H 3020-3150 Medium
C=C 1640-1680 Medium
Alkynes
C≡C-H 3300 Strong
-C≡C- 2100-2260 Medium
Alcohols
O-H 3400-3650 Strong, Broad
Amines
N-H 3200-3400 Medium, Broad
Aromatics
3030 Weak; 1660-2000 Weak
1450-1600 Medium

Carbonyls
C=O 1670-1780 Strong
Carboxylic Acids
O-H 2500-3100 Strong, Broad
Nitriles
CN 2210-2260 Medium

Fingerprint Region (< 1500 cm^{-1})

Single bonds not to H

C-C, C-O, C-F, C-Cl, C-N

Other vibrations - wags, bends, rocks.

Question 1 (21 points). I'm going to give up any idea of being cool, young and relevant – all these quotes are really old at this point. But fun! First up: *"Mom, look, I found something more fun than complaining!"*

Fill in the boxes below. Remember to consider ALL stereochemistry.

a)

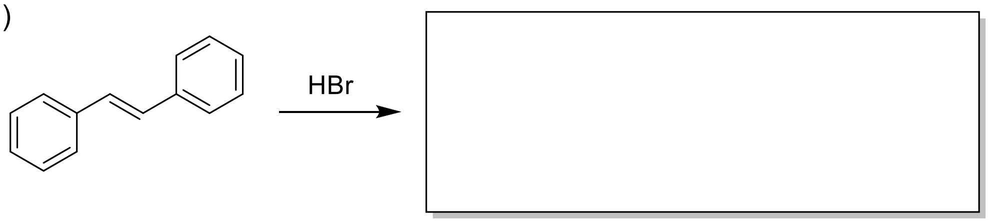

b)

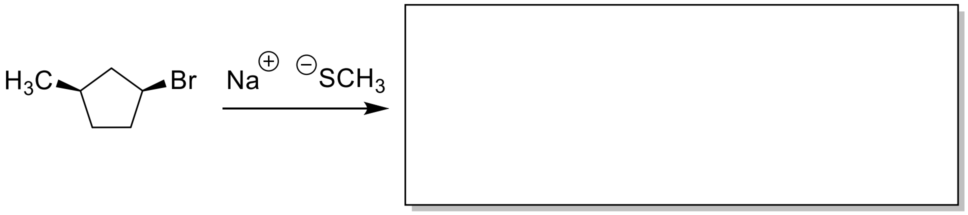

c)

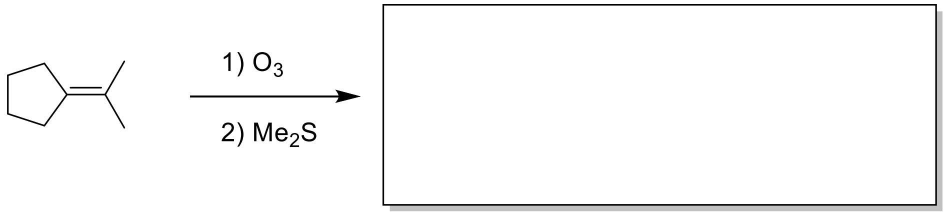

d)

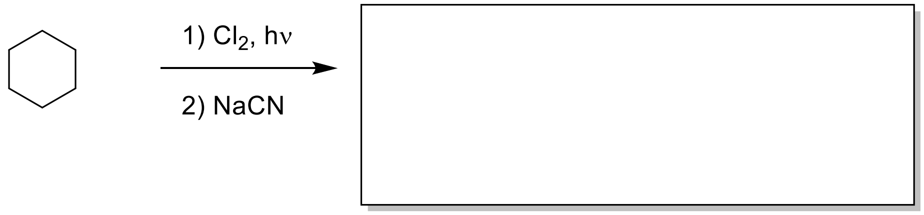

e)

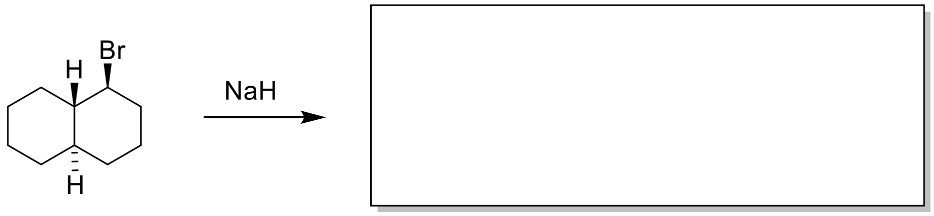

f)

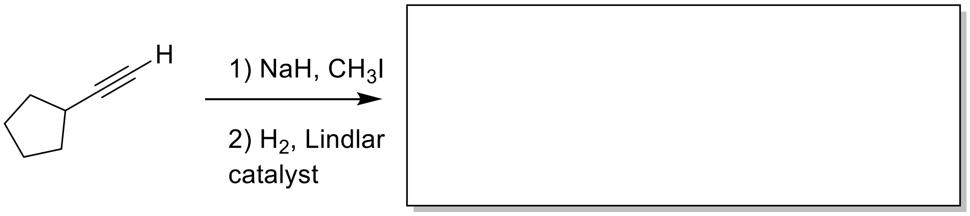

g)

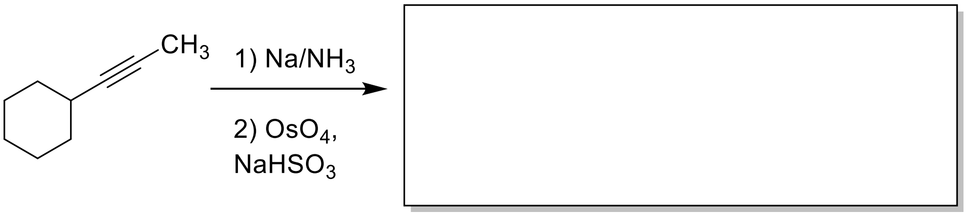

Question 2 (21 points). *"Call me Delta Airlines, because I can't handle all your extra baggage."* Fill in the boxes below. Remember to consider ALL stereochemistry, and make sure you pick the reactant that will give the ***greatest yield*** of product.

a)

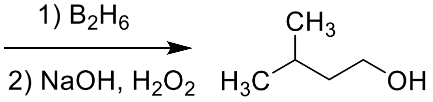

b)

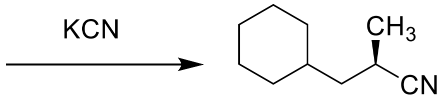

c)

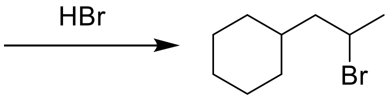

d)

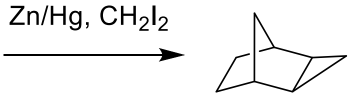

e)

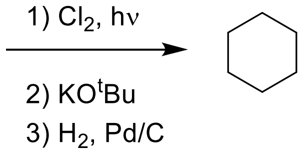

f)

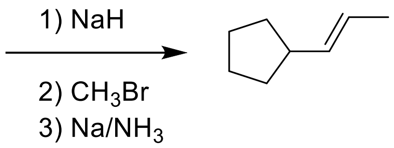

g)

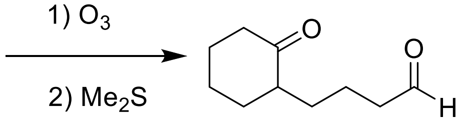

Question 3 (20 points). *"There are some things we don't want to know. Important things."*

a) (4 points) Why does reaction of molecule **A** with sodium cyanide give one product, whereas reaction with aqueous silver oxide gives two? ***Explain.***

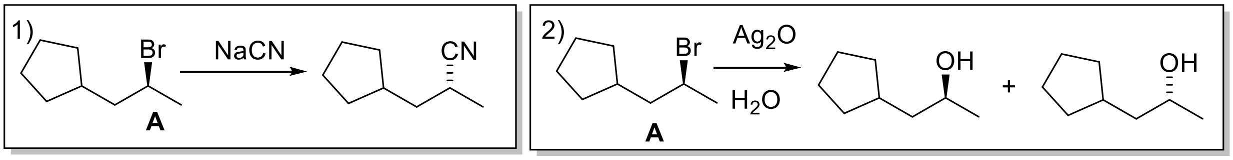

Explanation:

b) (4 points) Which reaction occurs FASTER, reaction 1 or reaction 2? ***Explain why.***

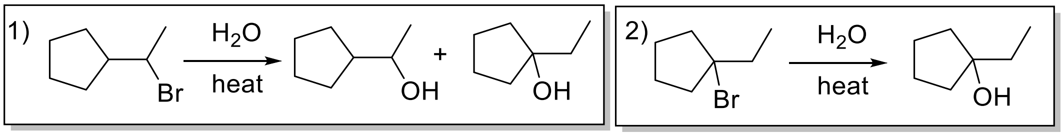

Explanation:

c) (4 points) Which reaction occurs FASTER, reaction 1 or reaction 2? ***Explain why.***

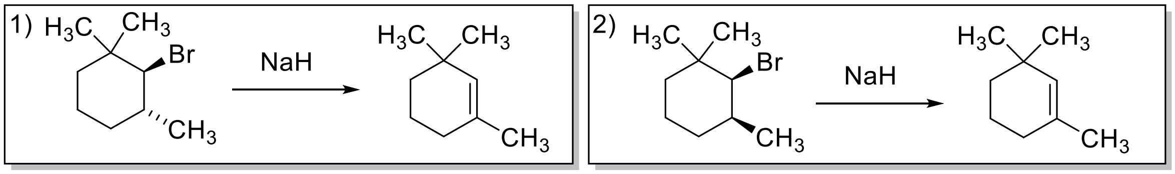

Explanation:

d) (4 points) Which reaction occurs FASTER, reaction 1 or reaction 2? ***Explain why.***

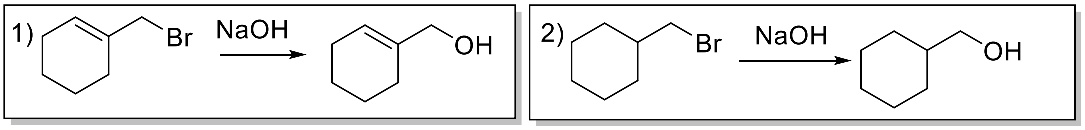

Explanation:

e) (4 points) Which reaction occurs FASTER, reaction 1 or reaction 2? ***Explain why.***

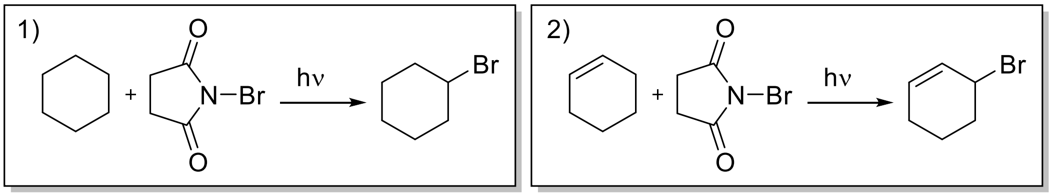

Explanation:

Question 4 (20 points). *"All the years I've wanted to be treated like an adult have blown up in my face."*
a) (4 points) Circle the SOLVENT which will cause the reaction below to occur FASTEST. Give a one sentence explanation for your answer.

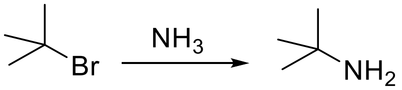

Solvents: Hexane 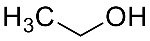 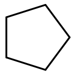

Explanation:

b) (4 points) Circle the SOLVENT which will cause the reaction below to occur FASTEST. Explain your choice.

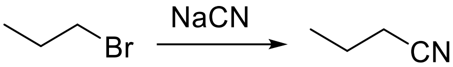

Solvents: water 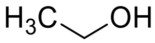 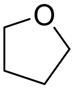

Explanation:

c) (3 points) Circle the molecule which has the HIGHEST boiling point. Give a one sentence explanation for your answer.

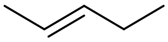 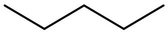 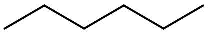 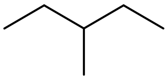 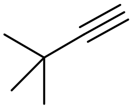

Explanation:

d) (3 points) Circle the molecule which has the HIGHEST boiling point. Give a one sentence explanation for your answer.

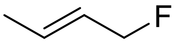 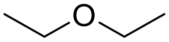 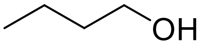 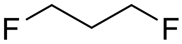 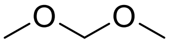

Explanation:

e) (4 points) Here is an IR spectrum. It can only be from one of the molecules to the right - circle that molecule, and ***explain why*** you made your choice.

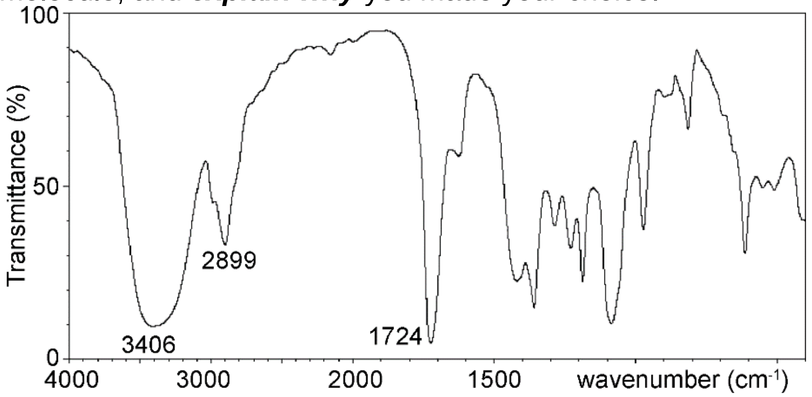

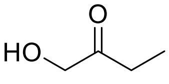 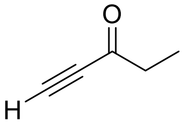

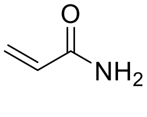 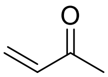

Explanation:

Question 5 (20 points). *"I can't promise I'll try, but I'll try to try".* In each case, explain why the proposed reaction FAILED, and draw the ACTUAL product.

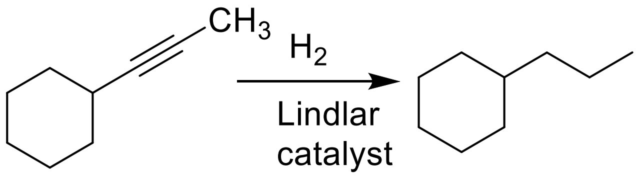

ACTUAL PRODUCT

Explanation:

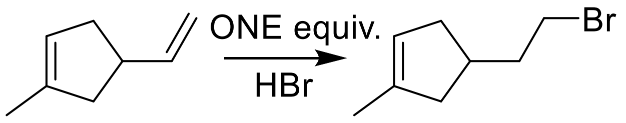

ACTUAL PRODUCT

Explanation:

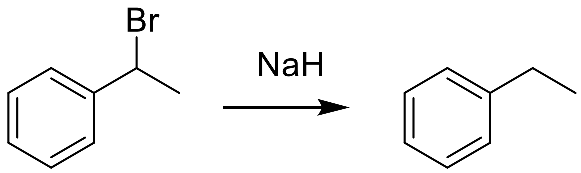

ACTUAL PRODUCT

Explanation:

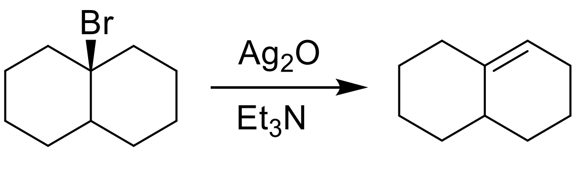

ACTUAL PRODUCT

Explanation:

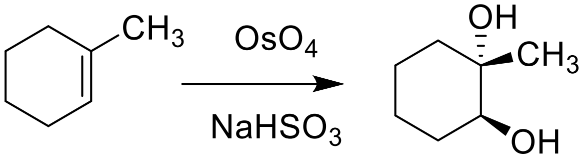

ACTUAL PRODUCT

Explanation:

Question 6. (20 points). *"People can come up with statistics to prove anything, Kent. 40% of all people know that."*

a) (12 points) Consider the following pairs of molecules. Determine the relationship between the molecules in each pair, i.e. are they ***enantiomers, diastereomers, constitutional isomers, conformational isomers, identical or unrelated***?

i)
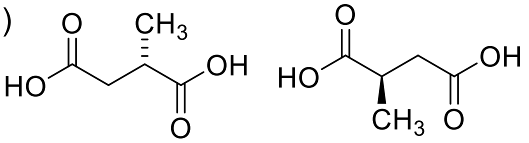
RELATIONSHIP:

v)
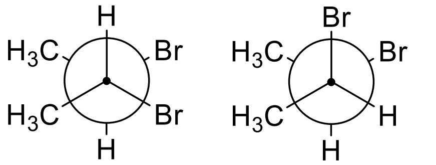
RELATIONSHIP:

ii)
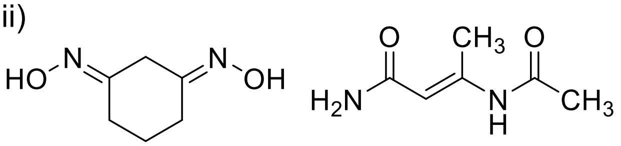
RELATIONSHIP:

vi)
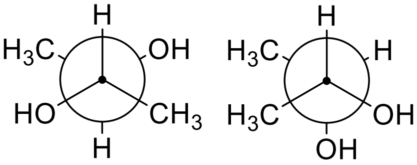
RELATIONSHIP:

iii)
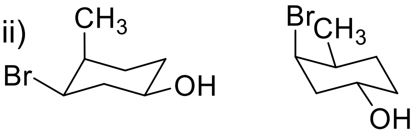
RELATIONSHIP:

vii)
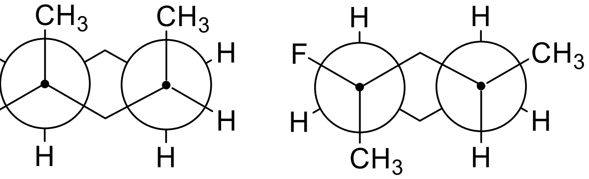
RELATIONSHIP:

iv)
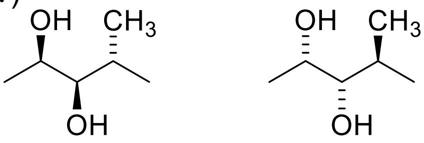
RELATIONSHIP:

vii)
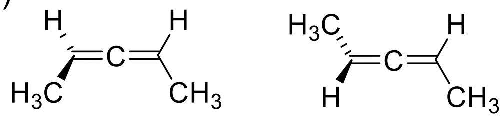
RELATIONSHIP:

b) (6 points) Circle the MOST favorable molecule/ion/transition state in each of the pairs below.

i)
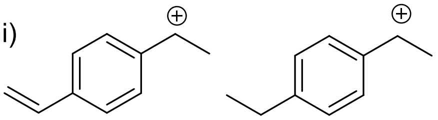

iv)
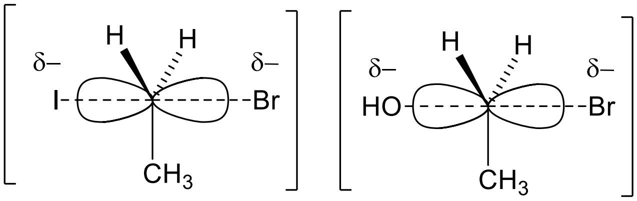

ii)
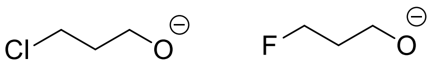

v)
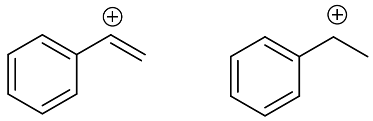

iii)
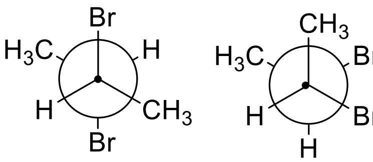

vi)
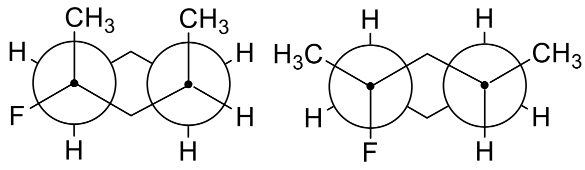

Question 7 (18 points). *"Oh God gets your prayers, but he just clicks delete without reading them..."* When you add HBr to a species with multiple double bonds such as **B**, you can get multiple products. You will explore this more in 008B, but here's a little taste.

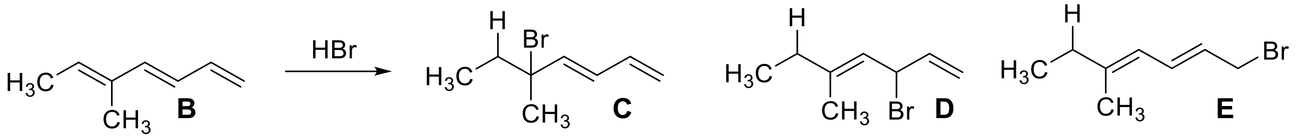

a) (6 points) Draw the immediate cationic product indicated by the arrow drawn below, and draw ***two more*** stable resonance structures, making sure you draw ***all necessary arrows***.

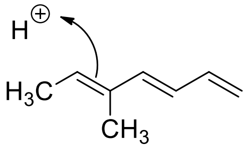

b) (2 points) Explain why the specific double bond in **B** shown in part a) reacts fastest.

c) (4 points) Based on your resonance structures from part a), draw the mechanism of formation of all 3 observed products **C**, **D** and **E**.

d) (2 points) Which of the resonance structures you drew in part a) is the most stable? Explain your answer.

e) (2 points) Which product, **C**, **D** or **E**, is the most stable, based on your knowledge of alkene stability? Explain your answer.

f) (2 points) Your answers to d) and e) should contradict each other. Think about that, then try and work out which product, **C**, **D** or **E**, will be formed in greatest yield. The point here is for reasoning, not picking the right answer!

Question 8 (20 points). *"All I want is what everyone wants: preferential treatment."*

Molecule **F** undergoes a substitution reaction to form **G**. Molecule **F** is enantiomerically pure, and has $[\alpha]_D$ = +20º. Enantiomerically pure **(-)-G** has a specific rotation $[\alpha]_D$ = -9º.

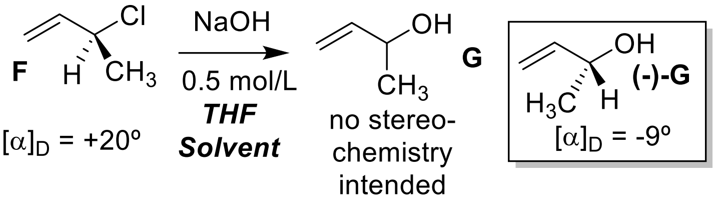

a) (3 points) There are two possible mechanisms for this process. Draw the ***transition state*** for an S_N2 reaction, and the ***cationic intermediate INCLUDING ALL STABLE RESONANCE STRUCTURES*** for the S_N1 process.

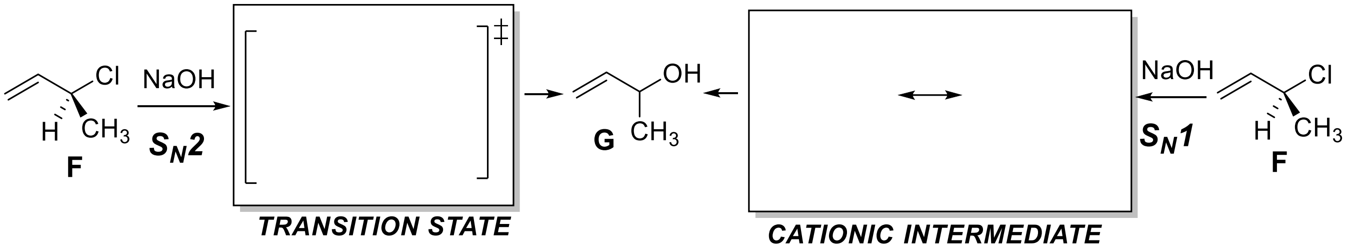

b) (2 points) When you perform the reaction under the conditions shown at the top of the page (i.e. [NaOH] = 0.5 mol/L, THF solvent), **G** is formed with a specific rotation $[\alpha]_D$ = -3º. ***Which mechanism(s) must be occurring to give that result?***

c) (3 points) When you perform the reaction with ***increased concentration***, i.e. [NaOH] = 1.0 mol/L, molecule **G** is formed with a specific rotation $[\alpha]_D$ = -7º. ***Explain why.***

d) (4 points) If you perform the reaction in water instead of THF, will the product have a LOWER or HIGHER $[\alpha]_D$? ***Explain your answer.***

e) (4 points) When you perform the reaction in water, you notice the appearance of a new product, **H.** Explain why this product is observed under these conditions. ***You will need to draw an arrow-pushing mechanism to receive full marks.***

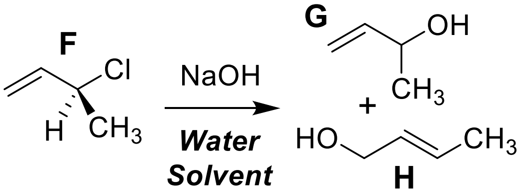

f) (4 points) When you perform the reaction of **F** with NaCN, a ***single enantiomer*** of product **I** is formed. Explain this observation, and explain why this is ***different*** to the reaction of **F** with NaOH.

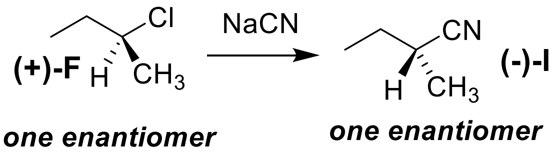

Question 9. (20 points). *"Young lady, in this house we obey the laws of thermodynamics!"*

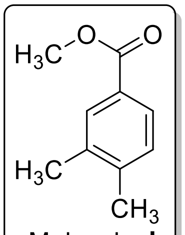

Molecule **J**

a) (4 points) Molecule **J** has a few chemically distinct hydrogen atoms: three of them are labeled H_a-H_c below, and each of them could be removed with strong base. One specific reaction is shown below, where molecule **J** reacts with the strong base $NaNH_2$. **Draw the *arrow-pushing mechanism*** for the conversion of **J** to the anion **J-**. For the reactants, label the ACID, and label the BASE.

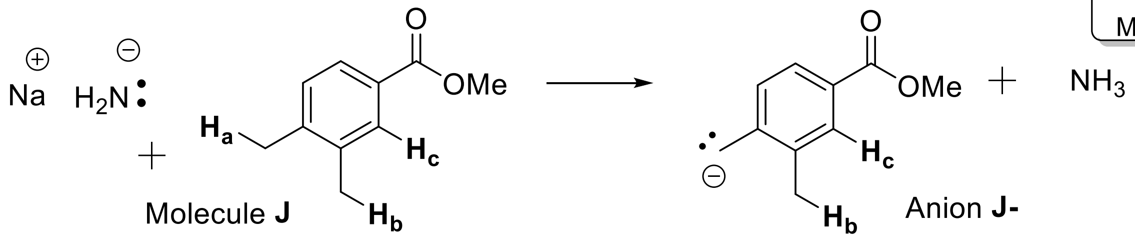

b) (8 points) Draw ***FOUR*** other *stable* resonance structures of anion **J-**. Include arrows to show electron movement.

c) (2 points) Explain why proton H_a is more acidic than the alkene proton H_c.

d) (4 points) Explain why proton H_a is more acidic than H_b. You will need to draw structures, including resonance structures, to answer this question fully.

e) (2 points) Write the correct hybridization next to each highlighted atom (i.e. the anionic carbon, and the oxygen in the ester group) in the structure below.

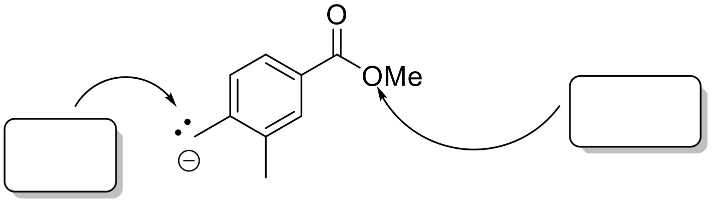

Question 10. (22 points). *"...if anyone asks you something you don't understand, just say protons."*

a) (10 points). Draw an arrow mechanism for the following transformation, which has MORE than one step!

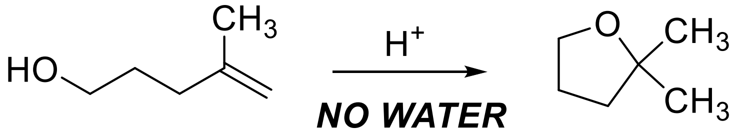

Mechanism:

b) (12 points). Draw an arrow mechanism for the following transformation, which has MORE than one step! Make sure you count the number of carbons…

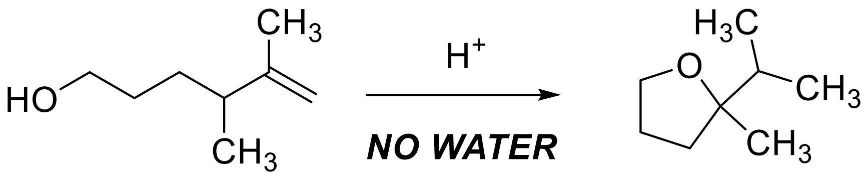

Mechanism:

BONUS Question (5 points). *"Operator! Give me the number for 911!"*

Chemical synthesis is the art of creating molecules from simple precursors by multiple sequential chemical reactions - you will do lots of this in 008B. Show how to create the following products from the starting materials below. You may use any reagents we have covered in class. Remember - each synthesis requires AT LEAST two reactions, and all of the syntheses are possible using reactions you have learned in this class.

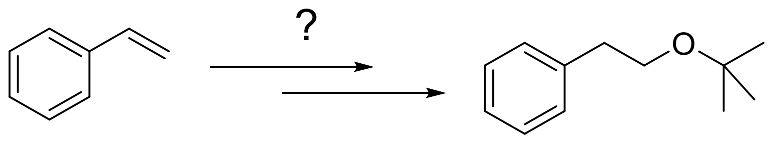

SCRATCH PAPER
