# Supplementary material for: Using Flipped Classroom Modules to Facilitate Higher Order Learning in Undergraduate Organic Chemistry
Source: J Chem Educ. 2024 Jan 25;101(2):490–500. doi: 10.1021/acs.jchemed.3c00907 (PMC10867829; doi:10.1021/acs.jchemed.3c00907)

Chem 008B Final Exam, Mar 21st, 2023

Name:____

Circle the FIRST LETTER of your LAST NAME:
A B C D E F G H I J K L M N O P Q R S T U V W X Y Z

STUDENT ID:___________________________

SEAT #:________________________________

LAB DAY / TIME: _______________________

Lab TA's Name: _________________________

READ AND SIGN

"I understand that any observed or suspected improper behavior on my part will be reported to Student Judicial Affairs."

Signature:________________________

Unsigned exams will not be graded

Time: 3.00 h
Write your name on all pieces of paper used for this exam. Write your answers on the exam sheet. There are 8 questions in this exam; you have THREE hours to do them. You may use your four sheets of notes, but NO OTHER pieces of paper.

Q1 ________	(21)	Q6 ________	(30)
Q2 ________	(21)	Q7 ________	(30)
Q3 ________	(20)	Q8 ________	(36)
Q4 ________	(22)		
Q5 ________	(20)	BONUS____	(5)

Total _______ (200)

DATA SHEET

Abbreviations:

br Broad peak

d doublet

t triplet

q quartet

qn quintet

sx sextet

sp septet

dd doublet of doublets

δ chemical shift (in ppm)

^{1}H Chemical shifts (ppm)

H designated in bold

Alkyl, RCH_3 (**H**)
0.8-1.0
Alkyl, RCH_2CH_3 (**H** of CH_2)
1.2-1.4
Alkyl, R_3CH (**H**)
1.4-1.7
Allylic, $R_2C{=}CRCH_3$ (**H**)
1.6-1.9
Ketone, R-COC**H**$_3$
2.1-2.6
Benzylic, Ar-C**H**$_3$
2.2-2.5
Acetylenic, RC≡C**H**
2.5-3.1
Alkyl chloride, RC**H**$_2$Cl
3.5-3.8
Alkyl bromide, RC**H**$_2$Br
3.3-3.6
Ether, ROC**H**$_2$R
3.3-3.9
Ester, RC**H**$_2$OC=OR
4.1-4.4
Alcohol, HO-C**H**$_2$-R
3.3-4.0
Vinylic, C=C-**H**
4.6-7.0
Aromatic, Ar**H**
6.5-9.0
Aldehyde, RCO**H**
9.5-10.0
Alcohol hydroxyl, -O**H**
0.5-6.0 (br)
Amino, R-N**H**$_2$
1.0-8.0 (br)
Phenolic, ArO**H**
4.5-7.7 (br)
Carboxylic, RCOO**H**
12-13 (br)

^{1}H Coupling Constants (Hz)

H-C-C-H (vicinal): 7 Hz

H-C=C-H (trans): 16 Hz

H-C=C-H (cis): 10 Hz

H-C-H (geminal, sp^3): 18 Hz

H-C-H (geminal, sp^2): 2 Hz

Aromatic *ortho*: 8 Hz

Aromatic *meta*: 2 Hz

^{13}C Chemical shifts (ppm)

C designated in bold

Alkyl, R**C**H$_3$
0-40
Alkyl, R**C**H$_2$R
10-50
Alkyl, R**C**HR$_2$
15-50
Alkyl halide, R**C**-X
10-65
Alcohol or ether,
C-OH, **C**-OR
50-90
Alkyne R-C≡**C**-R
60-90
Alkene, R$_2$C=**C**R$_2$
100-160
Aromatic **C**
100-170
Nitriles, -**C**N
120-130
Amides, -**C**ONR$_2$
150-180
Carboxylic acids, -**C**OOH
160-185
Esters, -**C**OOR
160-185
Aldehydes, -**CH**=O
185-200
Ketones, -R$_2$**C**=O
200-220

IR (cm^{-1})

Alkenes
=C-H 3020-3150 Medium
C=C 1640-1680 Medium
Alkynes
C≡C-H 3300 Strong
-C≡C- 2100-2260 Medium
Alcohols
O-H 3400-3650 Strong, Broad
Aromatics
3030 Weak; 1660-2000 Weak
1450-1600 Medium
Carbonyls
C=O 1670-1780 Strong
Carboxylic Acids
O-H 2500-3100 Strong, Broad
Nitriles
CN 2210-2260 Medium

Isotope Ratios

^{12}C : ^{13}C **98.89 % : 1.11%**

^{35}Cl : ^{37}Cl **75.8 % : 24.2 %**

^{79}Br : ^{81}Br **50.7 % : 49.3 %**

Nucleophiles/Bases

Excellent Nucleophiles (S_N2)	Ambiguous (Good Base and Nucleophile – Both S_N2 and E2)	Good Bases, Poor Nucleophiles (E2)	Weak Bases/ Nucleophiles (S_N1)
^-CN	^-OH	$(CH_3)_3CO^-$	H_2O
RS^-	^-OR	$^-NH_2$	ROH
I^-		H^-	
N_3^-		$^-NR_2$	
^-OAc			

Reaction Outcomes

	Poor Nu (e.g. H_2O, ROH	Good Nu, weak base (e.g. I^-, RS^-, ^-CN)	Good Nu, strong unhindered base (e.g. ^-OH, RO^-)	Strong, hindered base $(CH_3)_3CO^-$, R_2N^- AND H^-	Silver cation
methyl 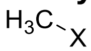	no rxn	S_N2	S_N2	S_N2	no rxn
primary 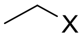	no rxn	S_N2	S_N2	E2	no rxn
secondary 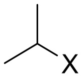	S_N1/E1	S_N2	E2	E2	S_N1/E1
tertiary 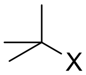	S_N1/E1	S_N1/E1	E2	E2	S_N1/E1
allyl/benzyl 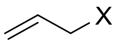 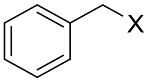	S_N1/E1	S_N2	S_N2	S_N2/E2	S_N1/E1

Question 1 (21 points). More Philomena Cunk quotes! These are great. "*Which was more culturally significant, the Renaissance, or Single Ladies by Beyoncé?"* Fill in the boxes.

a)

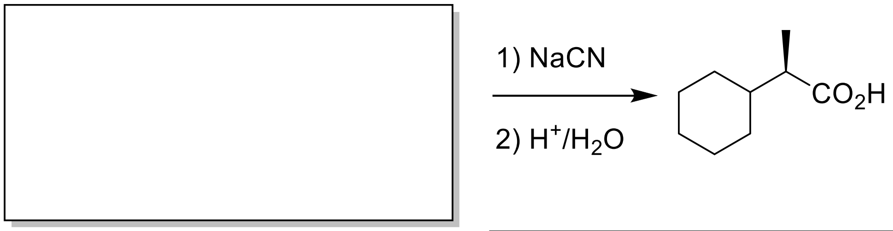

b)

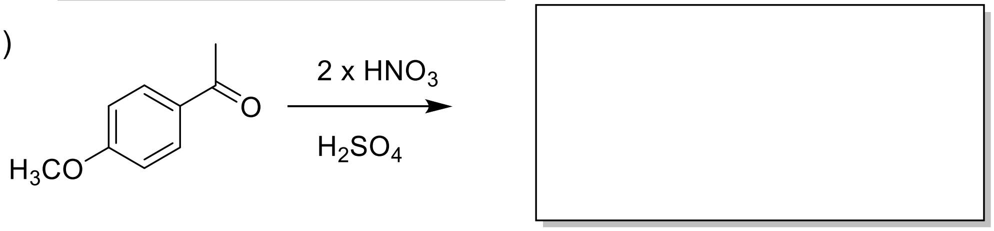

c)

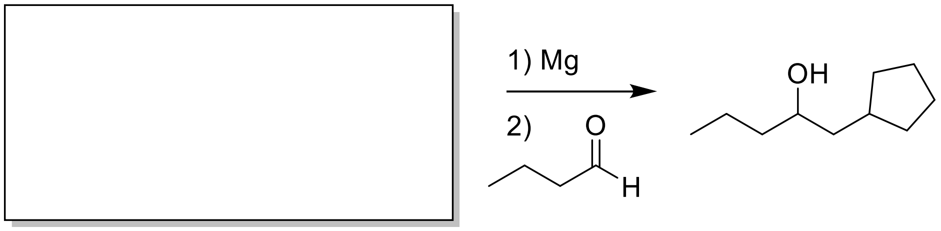

d)

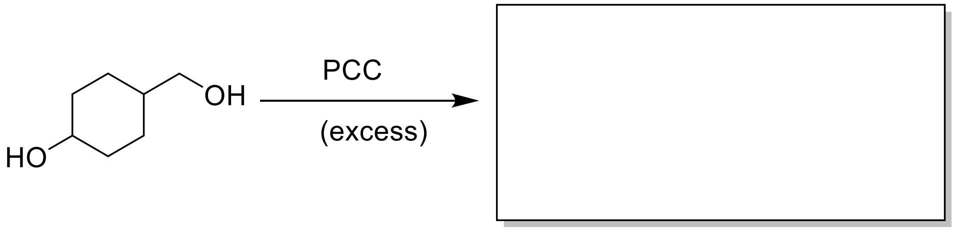

e)

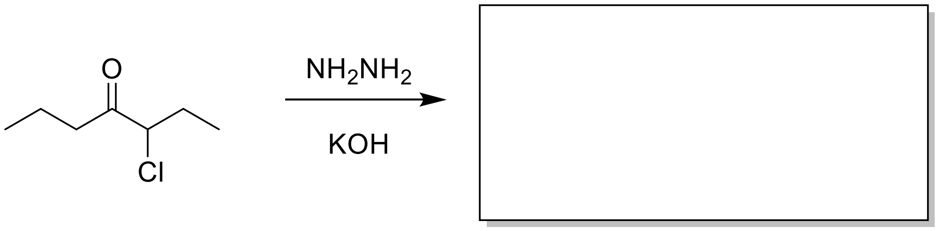

f)

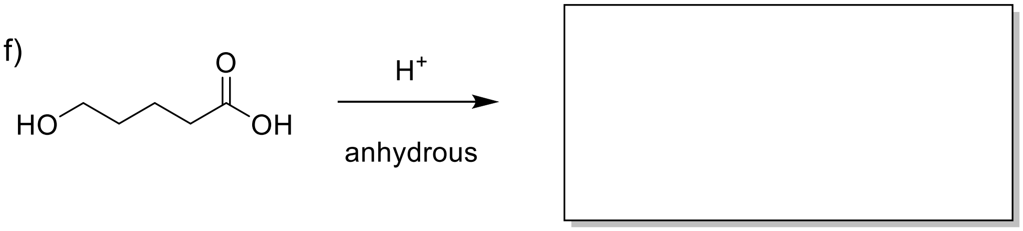

g)

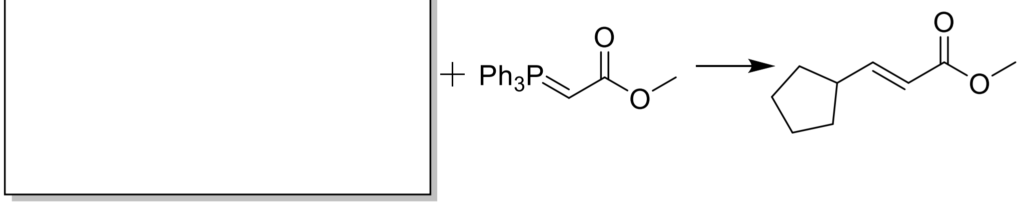

Question 2 (21 points). *"If you haven't heard of air, it's an invisible blend of gases so addictive, we suffer fatal withdrawal symptoms within minutes of our supply being cut off."* Fill in the boxes. ***Show all stereochemistry!***

a)

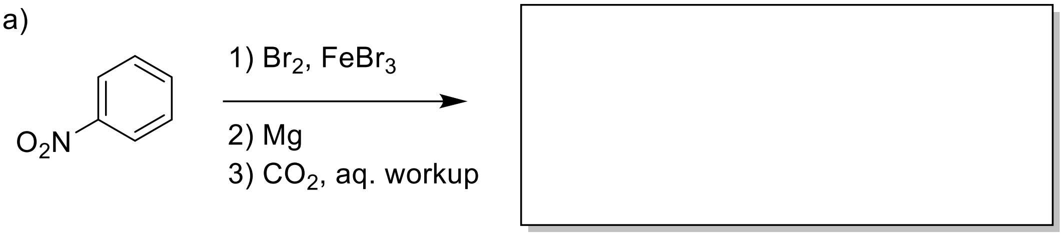

b)

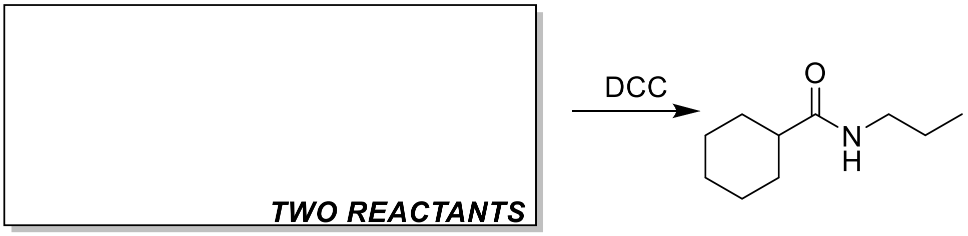

c)

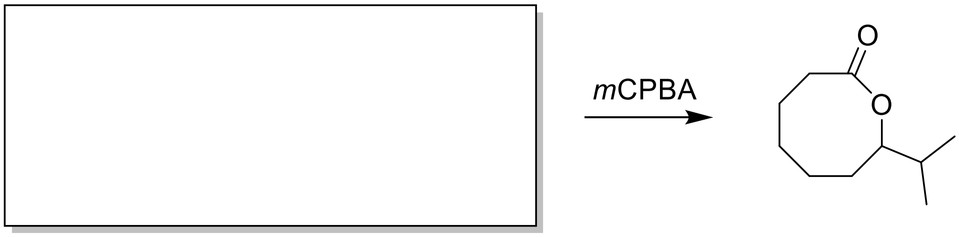

d)

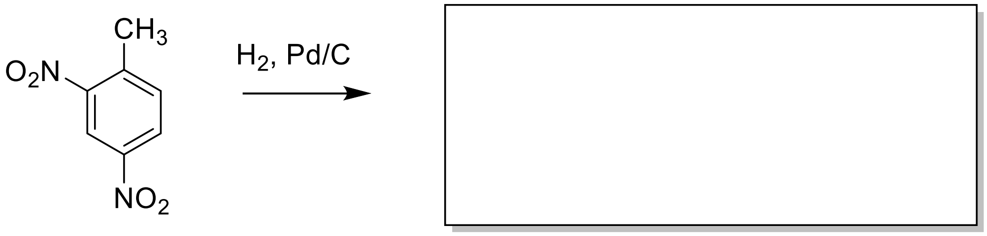

e)

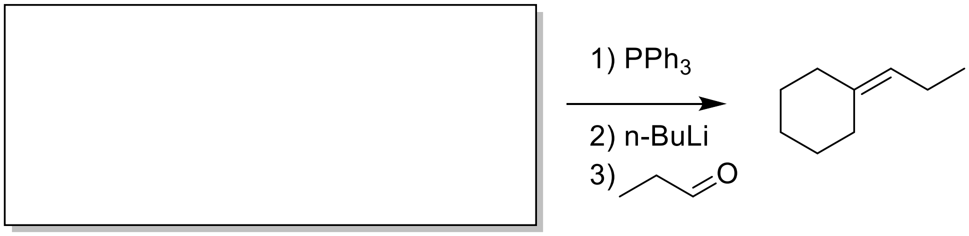

f)

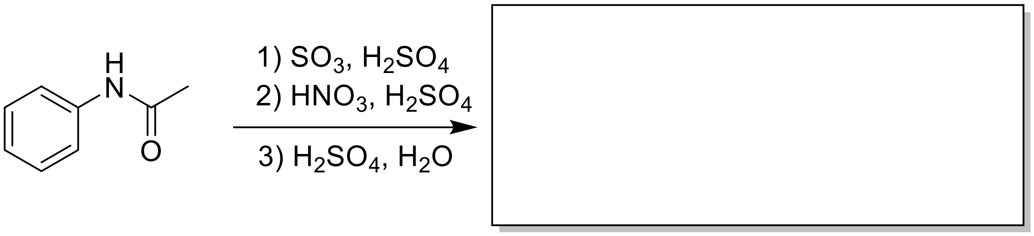

g)

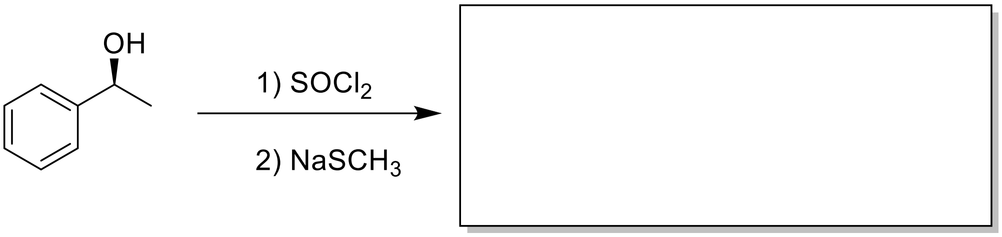

Question 3 (20 points). *"Americans back then weren't the humble, unassuming people they still aren't today."*

For each failed reaction proposed below, draw the ACTUAL product, and provide a one sentence explanation why the proposed reactions do not work.

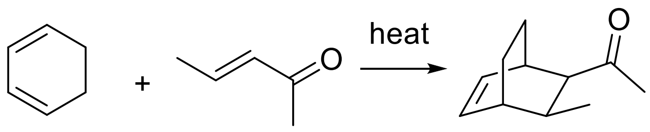

ACTUAL PRODUCT

Explanation:

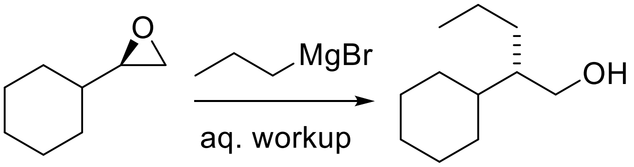

ACTUAL PRODUCT

Explanation:

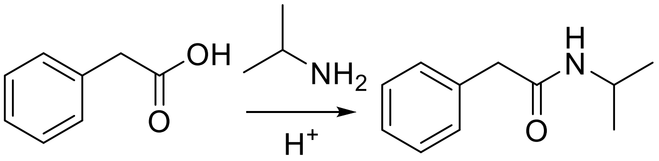

ACTUAL PRODUCT

Explanation:

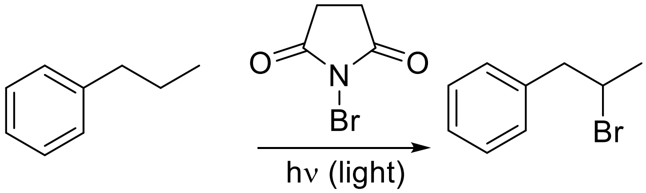

ACTUAL PRODUCT

Explanation:

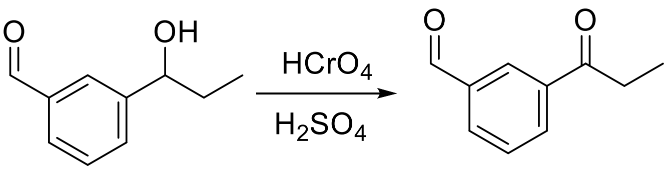

ACTUAL PRODUCT

Explanation:

Question 4 (22 points). *"Smartphones revolutionized the way humans interact, by providing a socially acceptable way to ignore everyone around us."*

Consider the four isomeric molecules **A-D**.

A 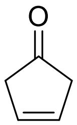

B 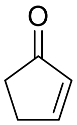

C 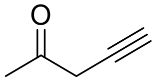

D 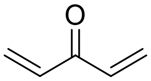

a) (3 points) Could you distinguish **B** and **C** by IR spectroscopy, assuming you do not have comparison spectra? Give a one sentence reason for your answer.

b) (4 points) How many peaks will be seen in the ^{13}C NMR spectrum of **A, B, C** and **D**?

A: **B:** **C:** **D:**

c) (4 points) How many peaks will be seen in the ^{1}H NMR spectrum of **A, B, C** and **D**? ***Do not consider coupling****,* only the number of different protons.

A: **B:** **C:** **D:**

d) (3 points) Could you distinguish **A** and **B** by UV spectroscopy, assuming you do not have comparison spectra? Give a one sentence reason for your answer.

e) (3 points) Which of the four molecules **A-D** will have the ***longest wavelength absorption*** in its UV spectrum? Give a one sentence reason for your answer.

f) (2 points) Now consider molecules **E** and **F**. Can you distinguish them by ^{1}H NMR spectroscopy? Give a one sentence reason for your answer.

E 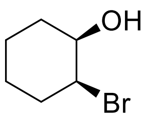

F 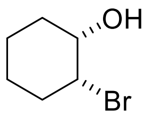

g) (3 points) Now consider molecules **G** and **H**. Can you distinguish them by ^{13}C NMR spectroscopy? Give a one sentence reason for your answer.

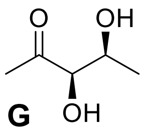

G
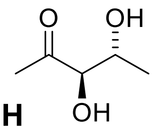

H

Question 5 (20 points). *"But Abraham Lincoln's story didn't have a happy ending. Five days after the North won, a terrible fate befell him. He was forced to go to the theater, to watch a play. He was put out of his misery by a kindly gunman, but cruelly, not until the third act."*

a) (4 points) Circle the molecule that reacts ***FASTEST*** with NaCN. Give a one sentence explanation for your answer.

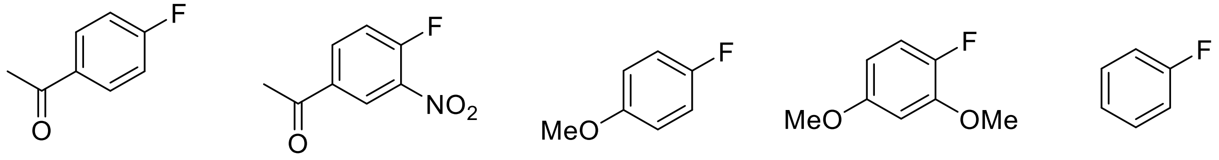

Explanation:

b) (4 points) Circle the molecule that reacts ***FASTEST*** with methylamine (CH_3NH_2). Give a one sentence explanation for your answer.

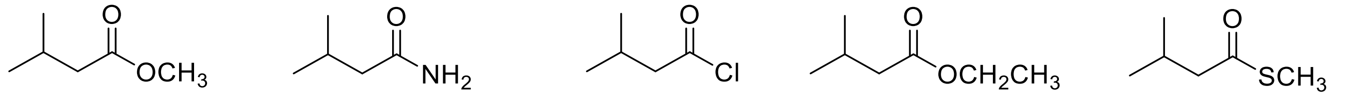

Explanation:

c) (4 points) Circle the molecule that is the ***MAJOR*** product of the reaction below. Give a one sentence explanation for your answer.

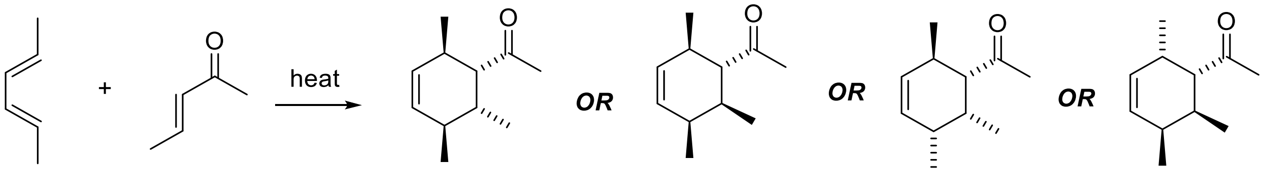

Explanation:

d) (4 points) Circle the molecule that reacts ***FASTEST*** with $NaBH_4$. Give a one sentence explanation for your answer.

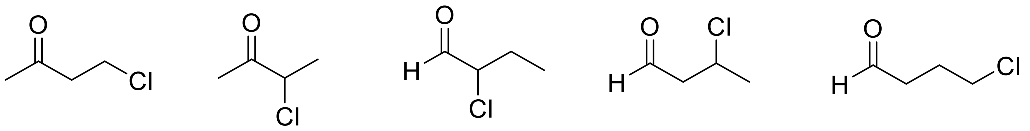

Explanation:

e) (4 points) Circle the molecule that will form the ***MOST STABLE CATION*** when reacted with Ag^+ ions (remember, silver ions promote S_N1 by forming insoluble AgBr salts, forcing cation formation). Give a one sentence explanation for your answer.

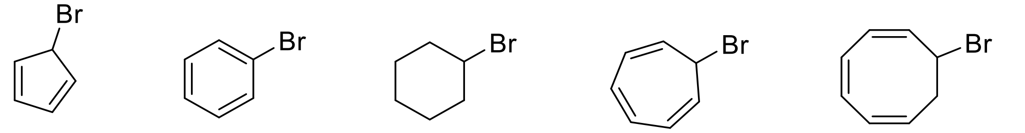

Explanation:

Question 6 (30 points). *"When the people in Boston got angry with the British, why did they just hold a prissy Tea Party instead of having a proper fight, like British men would?"*

a) (14 points) NMR data for molecule **J**, formula $C_5H_8O_3$, is given below. Your task is to work out the structure of molecule **J**.

IR: 3421 (br), 3055, 2958, 1732, 1636 cm^{-1}

UV: λ = 231 nm

^{13}C NMR: δ 166.5, 131.3, 128.2, 65.9, 60.3 ppm

^{1}H NMR spectrum:

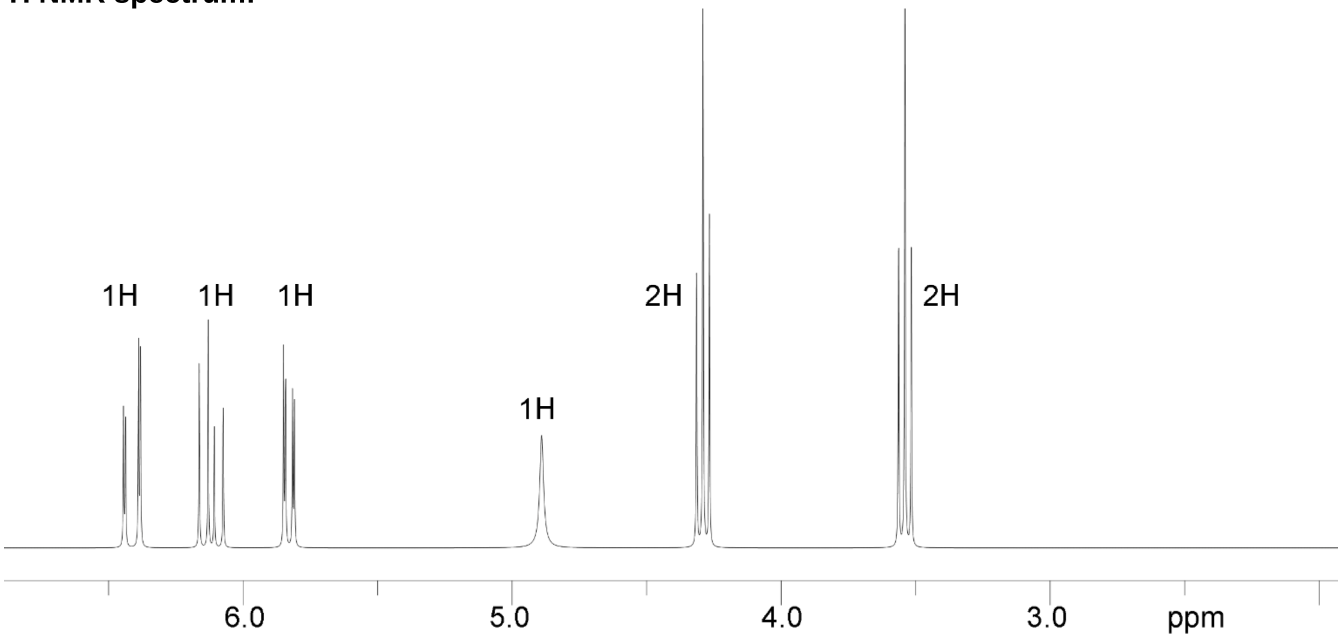

i) (1 points) How many degrees of unsaturation are there in the molecule?

ii) (3 points) From the IR, UV and ^{13}C NMR data, what functional groups are present? ***HINT:*** think about what the peak in the ***UV spectrum*** is telling you….

iii) (2 points) Describe the coupling patterns shown by the peaks at δ 6.4 ppm and 3.5 ppm.

iv) (8 points) What must be the structure of molecule **J**?

b) (16 points) Spectral data for molecule **K** is given below. Your task is to work out the structure of molecule **K**.

MS: 148 (100%), 149 (11.0%).

IR: 3011, 2861, 1705 cm^{-1}

^{13}C NMR: δ 206.2, 142.1, 132.2, 130.4, 128.3, 29.9, 18.9 ppm

^{1}H NMR spectrum:

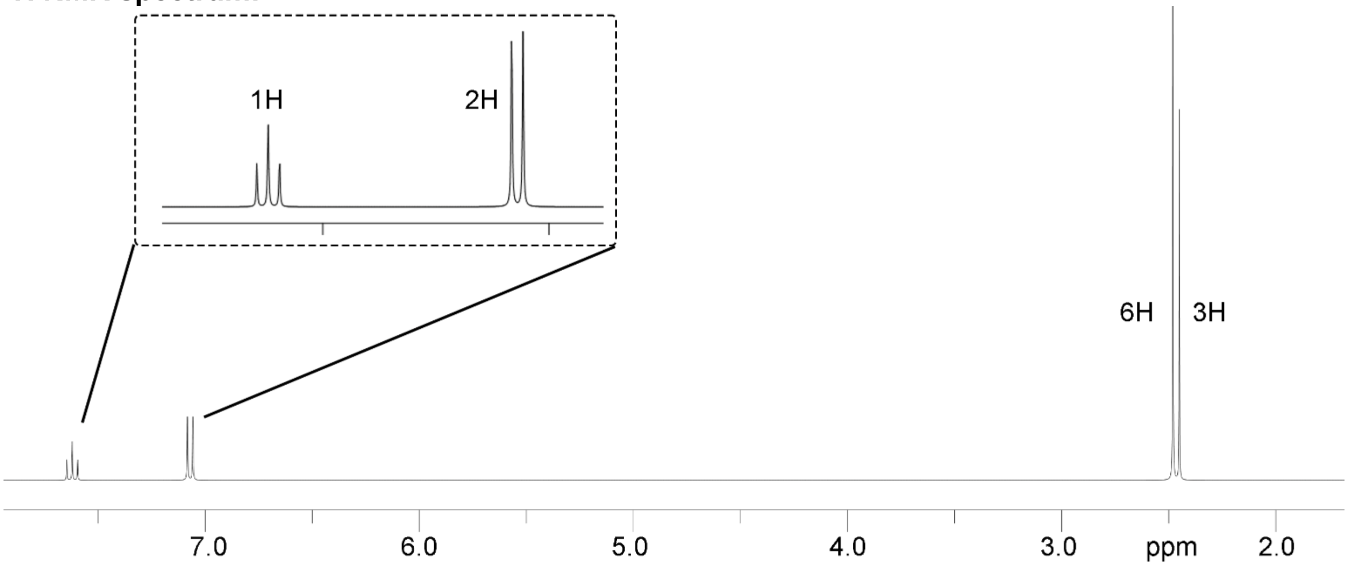

i) (1 points) First, MS data - what is the mass of the molecule?

ii) (2 points) From the MS data, how many carbons must be present in the molecule, and why?

iii) (2 points) Use the MS data, as well as the ^{1}H and ^{13}C NMR to determine the formula.

iv) (1 point) How many degrees of unsaturation are there?

v) (2 points) From the IR and ^{13}C NMR data, what functional groups are present?

vi) (8 points) What must be the structure of molecule **K**?

Question 7 (30 points). *"This straight, white supergroup known as the Founding Fathers drafted the most famous break-up text in history, the Declaration of Independence."*

Propose syntheses of the following molecules. You may use the indicated starting materials, as well as any inorganic reagents, triphenylphosphine, m-CPBA or organic reagents of FOUR carbons or fewer. Each synthesis requires MORE THAN ONE STEP!

a) (10 points)

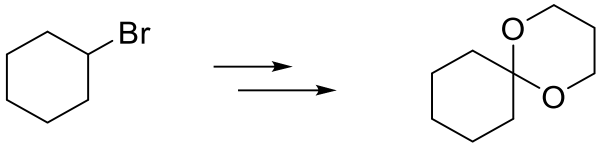

b) (10 points)

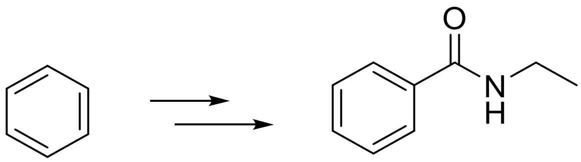

c) (10 points)

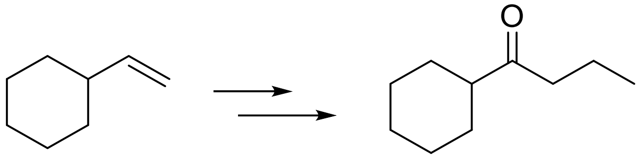

Question 8 (36 points). *"Descartes inspired an intellectual movement known as The Enlightenment, during which metrosexual elitists published essays which expanded humankind's horizons, in a manner that would go unmatched until the 1989 release of Belgian techno-anthem "Pump up the Jam"."*

a) (6 points) Draw the arrow-pushing mechanism for the following transformation.

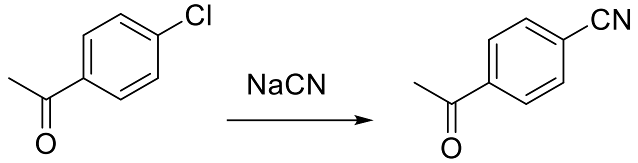

Mechanism:

b) (14 points). Draw the arrow-pushing mechanism for the following transformation.

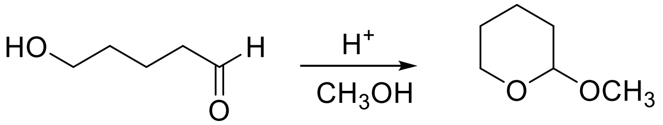

Mechanism:

c) (16 points). Dapsone is an anti-leprosy drug, and the molecule on the right is a more water-soluble, orally available variant. Draw the arrow-pushing mechanism for the synthesis of molecule **L**, a simplified version of the real reaction. This is a slight variation on reactions you've done in this class, but you know each step of the mechanism.

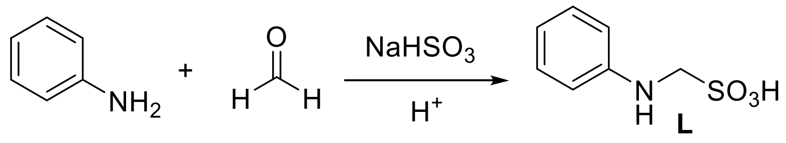

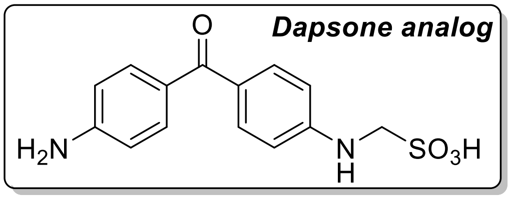

Mechanism:

BONUS (5 points). *"A world like this, where the masses toil for pennies while a tiny elite grow rich, seems so obviously unfair and unthinkable to us today, we can scarcely imagine what it must have been like."*

Here is a question that came up in my lab recently. We took an NMR spectrum of 2-pyridine-carboxaldehyde in water, and there were far more peaks than expected. Explain why the spectrum looks the way it does, and why this might happen to 2-pyridine-carboxaldehyde specifically. The more detail you go into, the more points you get!

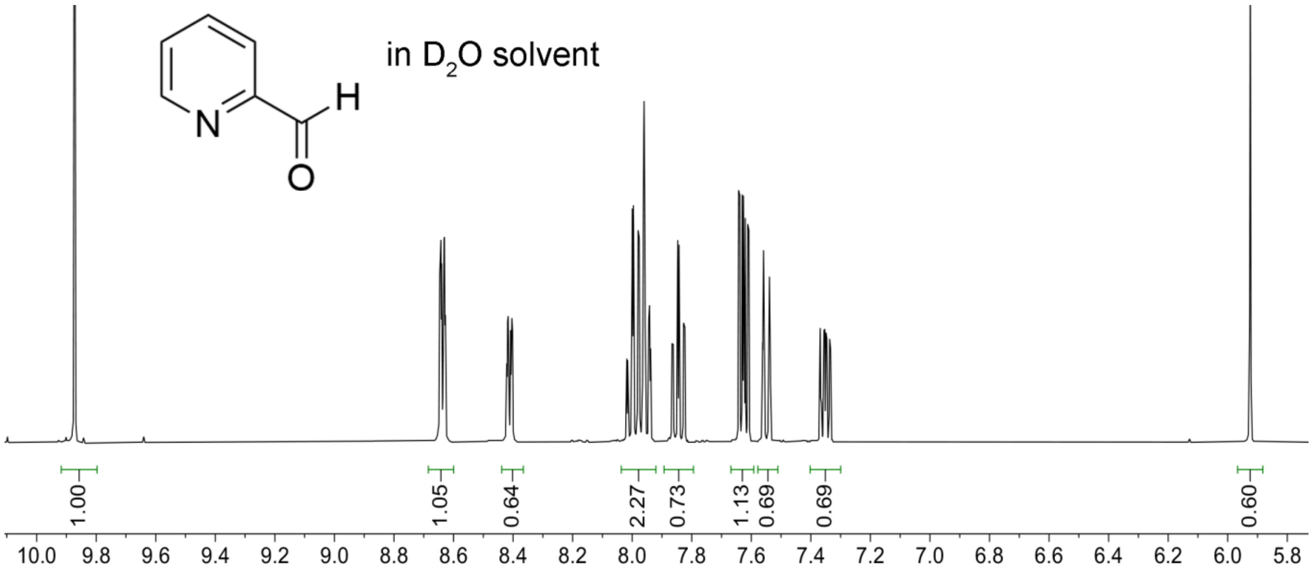

Supplement: Supplementary file 4 — ed3c00907_si_004.pdf [file ed3c00907_si_004.pdf]
